# Aerosol delivery, but not intramuscular injection, of adenovirus-vectored tuberculosis vaccine induces respiratory-mucosal immunity in humans

**DOI:** 10.1172/jci.insight.155655

**Published:** 2022-02-08

**Authors:** Mangalakumari Jeyanathan, Dominik K. Fritz, Sam Afkhami, Emilio Aguirre, Karen J. Howie, Anna Zganiacz, Anna Dvorkin-Gheva, Michael R. Thompson, Richard F. Silver, Ruth P. Cusack, Brian D. Lichty, Paul M. O’Byrne, Martin Kolb, Maria Fe C. Medina, Myrna B. Dolovich, Imran Satia, Gail M. Gauvreau, Zhou Xing, Fiona Smaill

**Affiliations:** 1 McMaster Immunology Research Centre,; 2 M.G. DeGroote Institute for Infectious Disease Research,; 3 Department of Medicine,; 4 Department of Pathology and Molecular Medicine, and; 5 Department of Chemical Engineering, McMaster University, Hamilton, Ontario, Canada.; 6 Department of Critical Care and Sleep Medicine, Case Western Reserve University School of Medicine, Cleveland, Ohio, USA.

**Keywords:** Infectious disease, Vaccines, Adaptive immunity, T cells, Tuberculosis

## Abstract

**Background:**

Adenovirus-vectored (Ad-vectored) vaccines are typically administered via i.m. injection to humans and are incapable of inducing respiratory mucosal immunity. However, aerosol delivery of Ad-vectored vaccines remains poorly characterized, and its ability to induce mucosal immunity in humans is unknown. This phase Ib trial evaluated the safety and immunogenicity of human serotype-5 Ad-vectored tuberculosis (TB) vaccine (AdHu5Ag85A) delivered to humans via inhaled aerosol or i.m. injection.

**Methods:**

Thirty-one healthy, previously BCG-vaccinated adults were enrolled. AdHu5Ag85A was administered by single-dose aerosol using Aeroneb Solo Nebulizer or by i.m. injection. The study consisted of the low-dose (LD) aerosol, high-dose (HD) aerosol, and i.m. groups. The adverse events were assessed at various times after vaccination. Immunogenicity data were collected from the peripheral blood and bronchoalveolar lavage samples at baseline, as well as at select time points after vaccination.

**Results:**

The nebulized aerosol droplets were < 5.39 μm in size. Both LD and HD of AdHu5Ag85A administered by aerosol inhalation and i.m. injection were safe and well tolerated. Both aerosol doses, particularly LD, but not i.m., vaccination markedly induced airway tissue–resident memory CD4^+^ and CD8^+^ T cells of polyfunctionality. While as expected, i.m. vaccination induced Ag85A-specific T cell responses in the blood, the LD aerosol vaccination also elicited such T cells in the blood. Furthermore, the LD aerosol vaccination induced persisting transcriptional changes in alveolar macrophages.

**Conclusion:**

Inhaled aerosol delivery of Ad-vectored vaccine is a safe and superior way to elicit respiratory mucosal immunity. This study warrants further development of aerosol vaccine strategies against respiratory pathogens, including TB and COVID-19.

**Trial registration:**

ClinicalTrial.gov, NCT02337270.

**Funding:**

The Canadian Institutes for Health Research (CIHR) and the Natural Sciences and Engineering Research Council of Canada funded this work.

## Graphical abstract

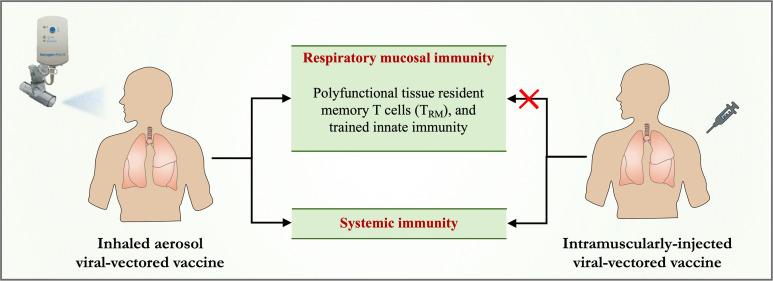

## Introduction

Pulmonary tuberculosis (TB) continues to be a major global health issue, accounting for 1.4 million deaths and 10 million new cases in 2019 (1). BCG, as the most administered human vaccine — which is given via the skin shortly after birth — has failed to effectively control TB in adults. In the last couple of decades, great strides have been made in developing new TB vaccine candidates (2). However, the vast majority of these vaccines were designed for the parenteral route of administration, which is known to induce poor respiratory mucosal immunity (3). Thus, a safe and effective boost vaccine strategy is urgently needed for much-improved protective immunity in the lung (2, 3).

Among the promising vaccine platforms is a recombinant replication-defective human serotype 5 adenovirus-vectored (AdHu5-vectored) TB vaccine expressing *M. tuberculosis* antigen 85A (AdHu5Ag85A). This vaccine has been extensively evaluated in a number of preclinical models, shown to be highly effective when administered via the respiratory tract, as opposed to its parenteral delivery (3, 4). Besides its superior effects in inducing lung tissue resident memory T cells (T_RM_) (3), AdHu5Ag85A delivered via the respiratory mucosa is able to elicit long-lasting memory airway macrophages and trained innate immunity (5, 6). It is widely believed that the most effective vaccine strategy ought to induce both innate memory and adaptive memory responses (5, 7). However, it remains unclear whether such highly compartmentalized distribution of immunity dictated by the route of Ad-vectored immunization is also true in humans. Although AdHu5Ag85A was evaluated successfully in healthy humans following i.m. injection (8, 9), its suitability for respiratory mucosal delivery and its safety and immunogenicity remain to be determined in healthy humans.

Recent studies have shown inhaled aerosol to be a safe and effective delivery method for a respiratory mucosal route of immunization in healthy humans with measles and MVA85A vaccines (10–13). However, these studies applied different technologies for aerosol delivery. Since aerosol characteristics and delivery efficiency may vary according to the type of vaccine, an aerosol delivery technology remains to be characterized and validated for administering Ad-vectored vaccine to the human airway. Given that a number of currently approved COVID-19 vaccines are also based on adenoviral vector, it is highly relevant to fully characterize an inhaled aerosol delivery technology for Ad-vectored vaccine and investigate its ability to induce respiratory mucosal immunity in preparation for its translation to respiratory mucosal COVID-19 vaccine strategies (14). Such next-generation COVID-19 vaccine strategies are urgently needed in the face of increasing breakthrough infections due to the variants of concern and waning vaccine-induced immunity (15).

In the present study, we have characterized the property of AdHu5Ag85A aerosol droplets generated by the Aeroneb Solo nebulizer. We evaluated the 2 aerosol doses and compared the safety and immunogenicity of the vaccine delivered via the respiratory mucosal route or i.m. route in previously BCG-vaccinated healthy adults. Our study is the first to our knowledge to safely deliver an Ad-vectored vaccine via inhaled aerosol to humans and to demonstrate its superiority in inducing respiratory mucosal immunity over i.m. injection.

## Results

**Figure 1:**
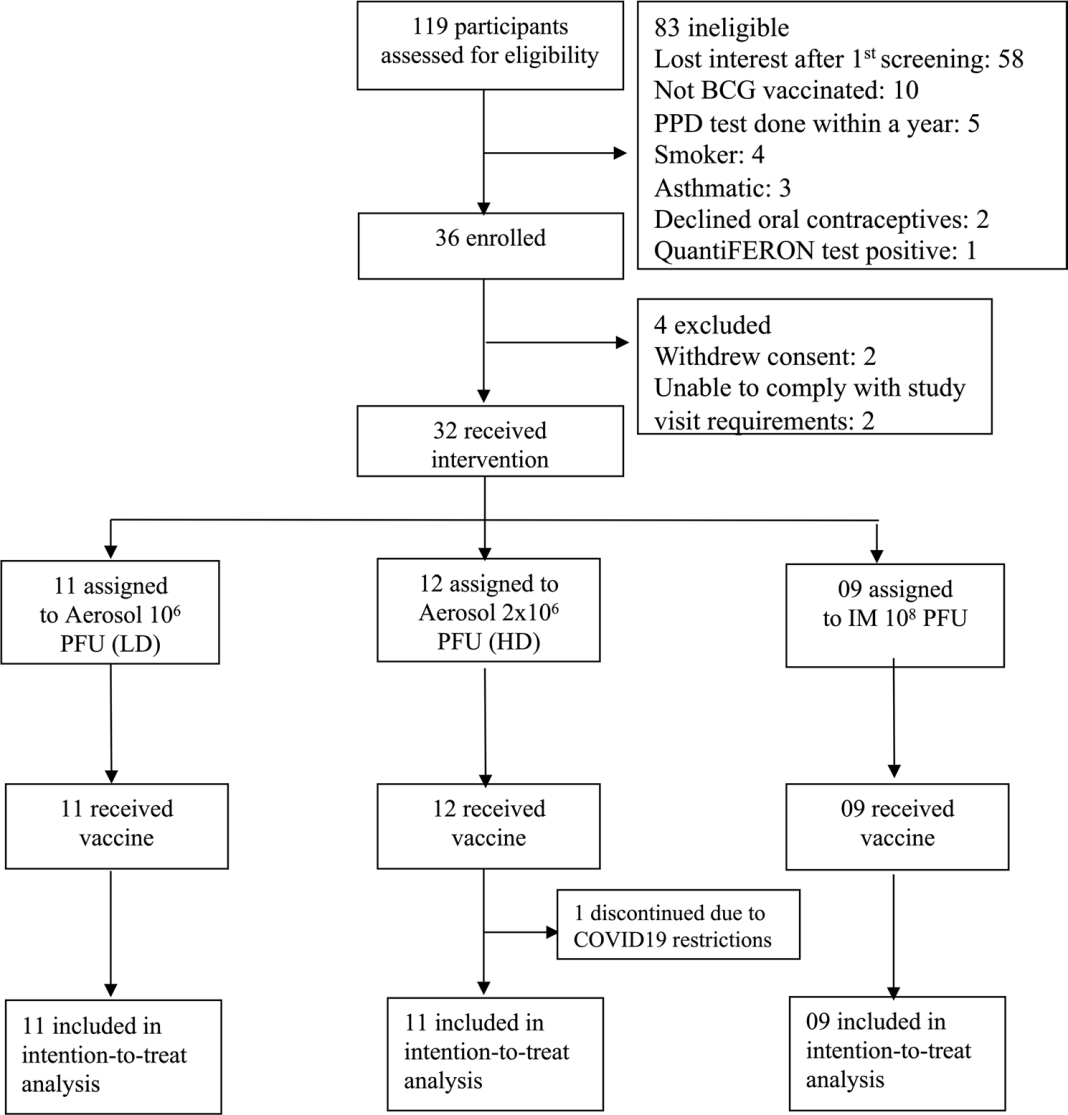
Trial profile. PPD, purified protein derivative; LD, low-dose aerosol; HD, high-dose aerosol; i.m.-intramuscular injection; PFU, plaque-forming unit.

During the period of March 2019 to February 2021, we enrolled 36 BCG-vaccinated healthy adults between 18 and 55 years of age at McMaster University Medical Centre. Four participants were excluded (2 withdrew consent and 2 were withdrawn before vaccination because they were unable to comply with the study visit requirements) and 1 did not complete any follow-up visits after vaccination because of COVID restrictions. Thirty-one participants completed the study: 11 in the low-dose (LD) aerosol group, 11 in the high-dose (HD) aerosol group, and 9 in the i.m. group (Figure 1). The demographic and baseline characteristics of the study participants were similar among study groups (Table 1).

### Characterization of inhaled aerosol delivery method and aerosol droplets using Aeroneb Solo device.

The Aeroneb Solo Micropump was selected to be part of the device set up for aerosol generation and delivery in our study (Supplemental Figure 1; supplemental material available online with this article; https://doi.org/10.1172/jci.insight.155655DS1). A fill volume (FV) of 0.5 mL in the nebulizer was determined to be optimal for vaccine delivery in saline. Subjects completed inhalation of this volume containing the vaccine via tidal breathing in approximately 2.5 minutes (Table 2). The emitted dose (ED) of vaccine available at the mouth was found to be approximately 50% of the loaded dose in the nebulizer (Table 2). The majority of aerosol droplets containing the vaccine were < 5.39 μm (85%), or between 2.08 and 5.39 μm in diameter, conducive to vaccine deposition in major airways. Thus, the amount of aerosol available at the mouth and subsequently deposited in the lung was 42.5% (16). The estimated rate of viable vaccine from aerosol droplets generated by the nebulizer was 17.4%. The dose loaded in the nebulizer for aerosol inhalation was, thus, corrected according to the estimated losses of vaccine within the device.

### Safety of inhaled aerosol and i.m.-injected AdHu5Ag85A vaccine.

Both LD (1 × 10^6^ PFU) and HD (2 × 10^6^ PFU) of AdHu5Ag85A administered by aerosol inhalation or the i.m. injection were safe and well tolerated. Respiratory adverse events were infrequent, mild, transient, and similar among groups (Table 3). I.m. injection was associated with a mild local injection site reaction in 2 participants. Systemic adverse events were also infrequent, mild, transient, and similar among groups (Table 3). One participant who received LD aerosol vaccine developed genital lesions consistent with primary HSV-1 infection the day following the week-2 bronchoscopy, and this condition resolved without complication with oral valacyclovir; one participant developed plantar fasciitis on day 13 following vaccination, which was attributed to mechanical strain and resolved with acetominophen. There were no grade 3 or 4 adverse events reported, nor any serious adverse events.

**Figure 2:**
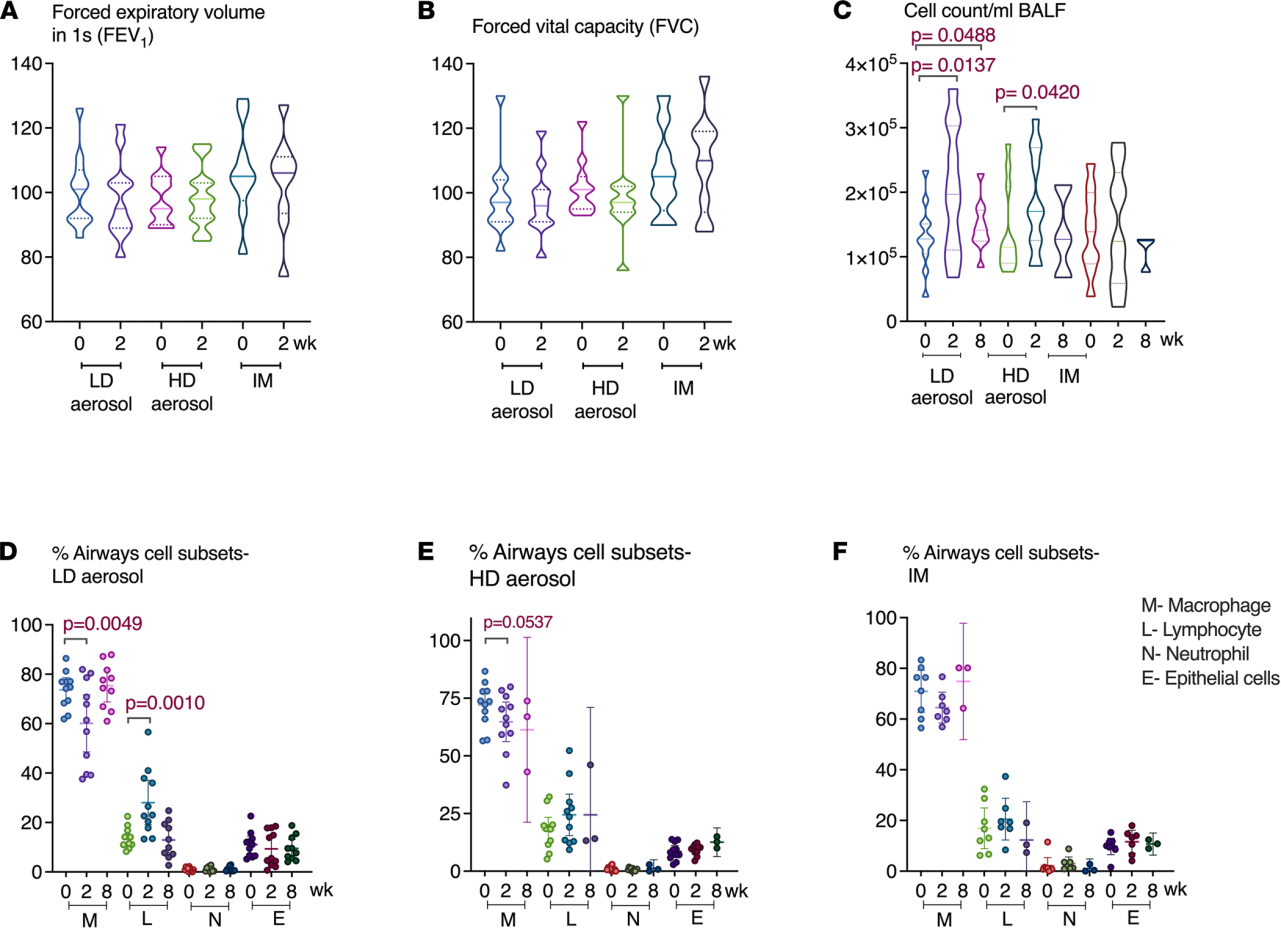
Respiratory function and bronchoalveolar cellular responses following aerosol or intramuscular vaccination. (**A** and **B**) Lung function was assessed as FEV_1_ and FVC at baseline and 2 weeks after LD aerosol (*n =* 11), HD aerosol (*n =* 11), or i.m. (*n =* 9) vaccination. (**C**–**F**) Frequencies of differential cells including macrophages, lymphocytes, neutrophils, and epithelial cells in BALF from LD aerosol, HD aerosol, and i.m. vaccine cohorts. Violin plots show the median and quartiles. Data in dot plots are expressed as the mean value (horizontal line) with 95% CI. Wilcoxon matched pairs signed-rank test was used to compare various time points with baseline values within the same vaccination group.

There were no clinically significant abnormalities of laboratory tests at weeks 2, 4, and 12 following vaccination. Follow-up respiratory functional determinations forced expiratory volume in the first second (FEV_1_)and forced vital capacity (FVC) were similar to baseline values in all participants across all 3 groups (Figure 2, A and B).

Bronchoscopy and bronchoalveolar lavage were generally well tolerated in all participants. As expected, in some participants, the procedures were associated with mild cough, sore throat, low-grade fever, headache, and a transient drop in FEV_1_. The appearance of the bronchial mucosa was judged as normal in all participants at each time point. Adequate bronchoalveolar lavage fluid (BALF) volumes were obtained following bronchoalveolar lavage, and on average, 10 to 20 million total cells were obtained.

### Aerosol AdHu5Ag85A vaccination induces robust and sustainable Th1 responses in the airway.

Bronchoalveolar lavage was obtained successfully at baseline and at 2 and 8 weeks after vaccination from all participants with a median return volume of 87.5 mL (IQR, 72.5–98) from a total of 160 mL saline instilled and a median total cell number of 0.14 million/mL BALF (IQR, 0.1–0.2). Cellularity in the airway significantly increased 2 weeks after both LD and HD aerosol vaccination, and in the LD aerosol group, cellularity remained significantly heightened up to 8 weeks after vaccination compared with baseline (Figure 2C). Both LD and HD aerosol vaccination led to a transient reduction in airway macrophages, but the lymphocyte counts significantly increased only in LD cohort (Figure 2, D and E). Importantly, both neutrophils and epithelial cells in the airway remained either absent or unaltered following aerosol vaccination (Figure 2, D and E), indicating no significant airway inflammation except vaccine-induced lymphocytic responses. In comparison, there were no marked changes in total cellularity and any leukocyte subsets in the airway after i.m. vaccination (Figure 2, C and F).

**Figure 3:**
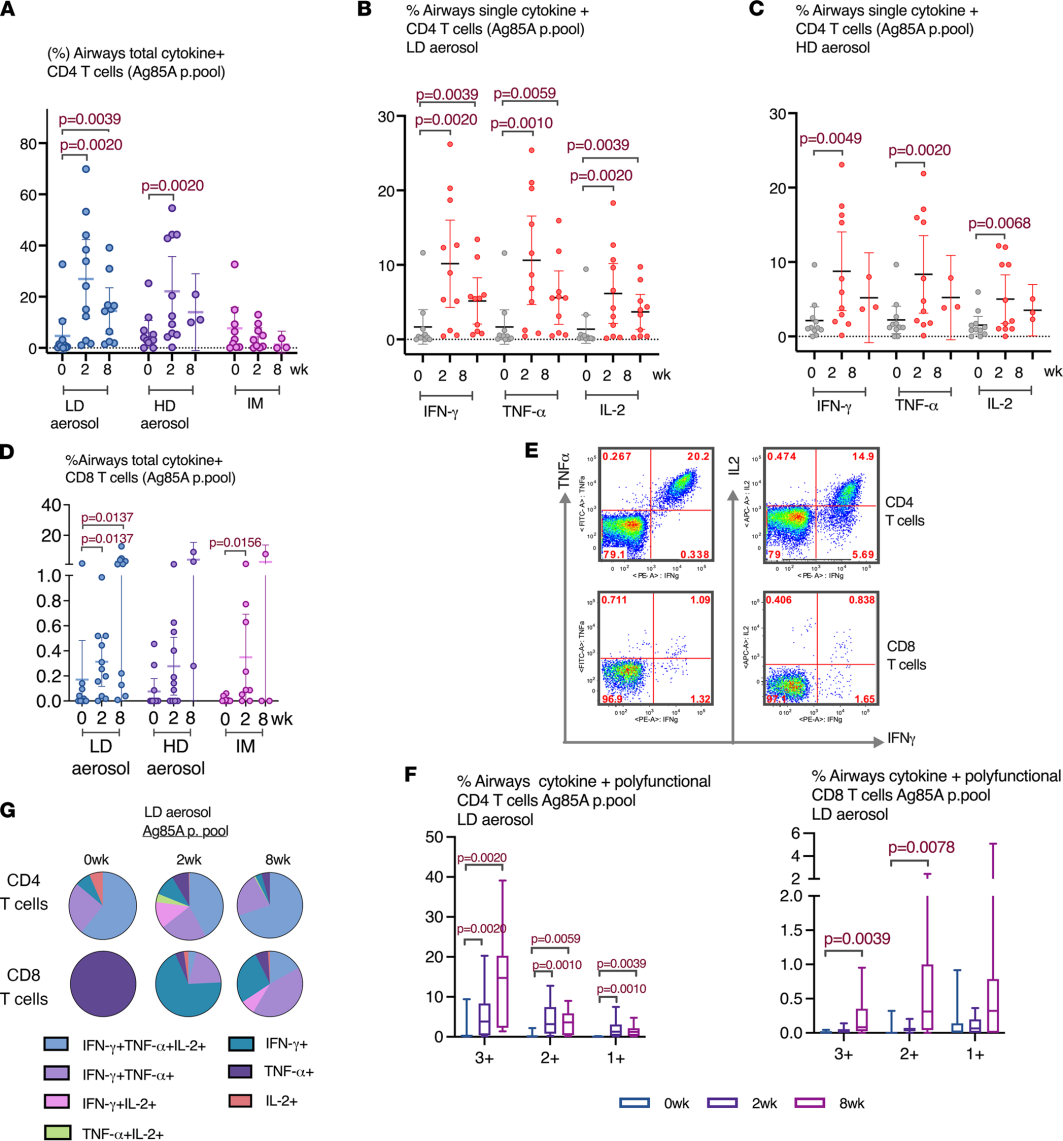
Induction of multifunctional T cells in the airways following aerosol or i.m. vaccination. (**A**) Frequencies of airway antigen–specific combined total-cytokine–producing CD4^+^ T cells at various time points in LD aerosol, HD aerosol, and i.m. cohorts. (**B**) Frequencies of airway single-cytokine–producing CD4^+^ T cells at various time points in LD aerosol cohort. (**C**) Frequencies of airway single-cytokine–producing CD4^+^ T cells at various time points in HD aerosol cohort. (**D**) Frequencies of airway antigen–specific combined total-cytokine–producing CD8^+^ T cells at various time points in LD aerosol, HD aerosol, and i.m. cohorts. (**E**) Representative dot plots of airways CD4^+^ and CD8^+^ T cells expressing IFN-γ, TNF-α, and IL-2 at wk2 from LD aerosol participants. (**F**) Frequencies of airways polyfunctional (triple/3+, double/2+, and single/1+ cytokine^+^) antigen-specific CD4^+^ and CD8^+^ T cells at various time points in LD aerosol group. (**G**) Median proportions displayed in pie chart of antigen-specific airways CD4^+^ and CD8^+^ T cells expressing a specific single or combination of 2 or 3 cytokines at various time points in LD aerosol group. Data in dot plots are expressed as the mean value (horizontal line) with 95% CI. Box plots show mean value (horizontal line) with 95% CI (whiskers), and boxes extend from the 25th to 75th percentiles. Wilcoxon matched pairs signed-rank test was used to compare various time points with baseline values within the same vaccination group.

Evaluation of Th1 responses in the airways (BALF) cells was performed by intracellular cytokine immunostaining and flow cytometry (the gating strategy shown in Supplemental Figure 2). It showed that both LD and HD aerosol AdHu5Ag85A markedly increased Ag85A peptide pool–specific (Ag85A p. pool–specific) or reactive, IFN-γ–, TNF-α–, and/or IL-2–producing CD4^+^ T cells in the airways at 2 weeks after vaccination compared with the respective baseline responses (Figure 3, A–C). On average, the total Ag-specific cytokine-producing CD4^+^ T cells represented approximately 25% of all CD4^+^ T cells at 2 weeks after LD or HD aerosol, and they remained significantly elevated up to 8 weeks in the airways of LD cohort (Figure 3, A and B). In contrast, i.m. AdHu5Ag85A vaccination failed to induce Ag-specific CD4^+^ T cells in the airways (Figure 3A). Compared with CD4^+^ T cells, although the levels of airway CD8^+^ T cell responses were much smaller, they were significantly increased at both 2 and 8 weeks, particularly following LD aerosol vaccination (Figure 3D). Of interest, there was also a small but increased number of CD8^+^ T cells at 2 weeks after i.m. vaccination.

Analysis of polyfunctionality of vaccine-activated CD4^+^ and CD8^+^ T cells reactive to Ag85A in the airways revealed that LD aerosol vaccination led to induction of a higher magnitude of CD4^+^ T cells that coexpressed IFN-γ, TNF-α, and IL-2 (3+) and any of 2 cytokines (2+) compared with those producing single cytokine (1+) (Figure 3, E and F). Importantly, polyfunctional CD4^+^ T cells remained significantly increased over the baseline up to 8 weeks. Similarly, LD aerosol vaccination also significantly increased the polyfunctional CD8^+^ T cells, particularly at 8 weeks, in the airways, though at a much lower overall magnitude compared with CD4^+^ T cells (Figure 3F). In comparison, HD aerosol vaccination led to significantly increased polyfunctional CD4^+^ but not CD8^+^ T cells reactive to Ag85A (Supplemental Figure 3, A and B).

Given that the LD aerosol AdHu5Ag85A was consistently highly immunogenic, we profiled the polyfunctional CD4^+^ and CD8^+^ T cells in the airways of the LD cohort in greater detail. While at 2 weeks, a greater proportion of Ag85A-reactive CD4^+^ T cells were polyfunctional (IFN-γ^+^TNF-α^+^IL-2^+^, IFN-γ^+^TNF-α^+^, or IFN-γ^+^IL-2^+^), with some of them also being single cytokine producers, at 8 weeks, the vast majority of them (>95%) became polyfunctional (Figure 3G). In comparison, most of the CD8^+^ T cells at 2 weeks were single-cytokine producers (TNF-α^+^), but at 8 weeks, the majority of them turned to be polyfunctional, mostly being IFN-γ^+^TNF-α^+^ (Figure 3G).

Since the trial participants were previously BCG vaccinated, we examined the overall T cell reactivity to stimulation with multimycobacterial antigens. We found considerable CD4^+^ T cells present in the airways to be reactive to a cocktail of mycobacterial antigens even prior to vaccination, and they remained unaltered after LD, HD, or i.m. vaccination (Supplemental Figure 3C). These BCG-specific CD4^+^ T cells in the airways of LD group were mostly polyfunctional (Supplemental Figure 3, D and E). Similarly, small numbers of preexisting BCG-specific CD8^+^ T cells in the airways were not altered by aerosol or i.m. vaccination (Supplemental Figure 3F).

The above data suggest that inhaled aerosol, but not i.m., AdHu5Ag85A vaccination can induce robust antigen-specific T cell responses within the respiratory tract. Furthermore, a LD (1 × 10^6^ PFU) aerosol vaccination is superior to a HD (2 × 10^6^ PFU) aerosol in inducing robust and sustainable respiratory mucosal immunity. The mucosal responses induced by AdHu5Ag85A vaccine are predominantly polyfunctional CD4^+^ T cells in nature, with some levels of polyfunctional CD8^+^ T cells. The preexisting CD4^+^ T cells of multimycobacterial antigen specificities in the airway of BCG-vaccinated trial participants were not significantly impacted by AdHu5Ag85A aerosol vaccination.

### Aerosol vaccination induces airway T_RM_ expressing the lung-homing molecule α4β1 integrin.

**Figure 4:**
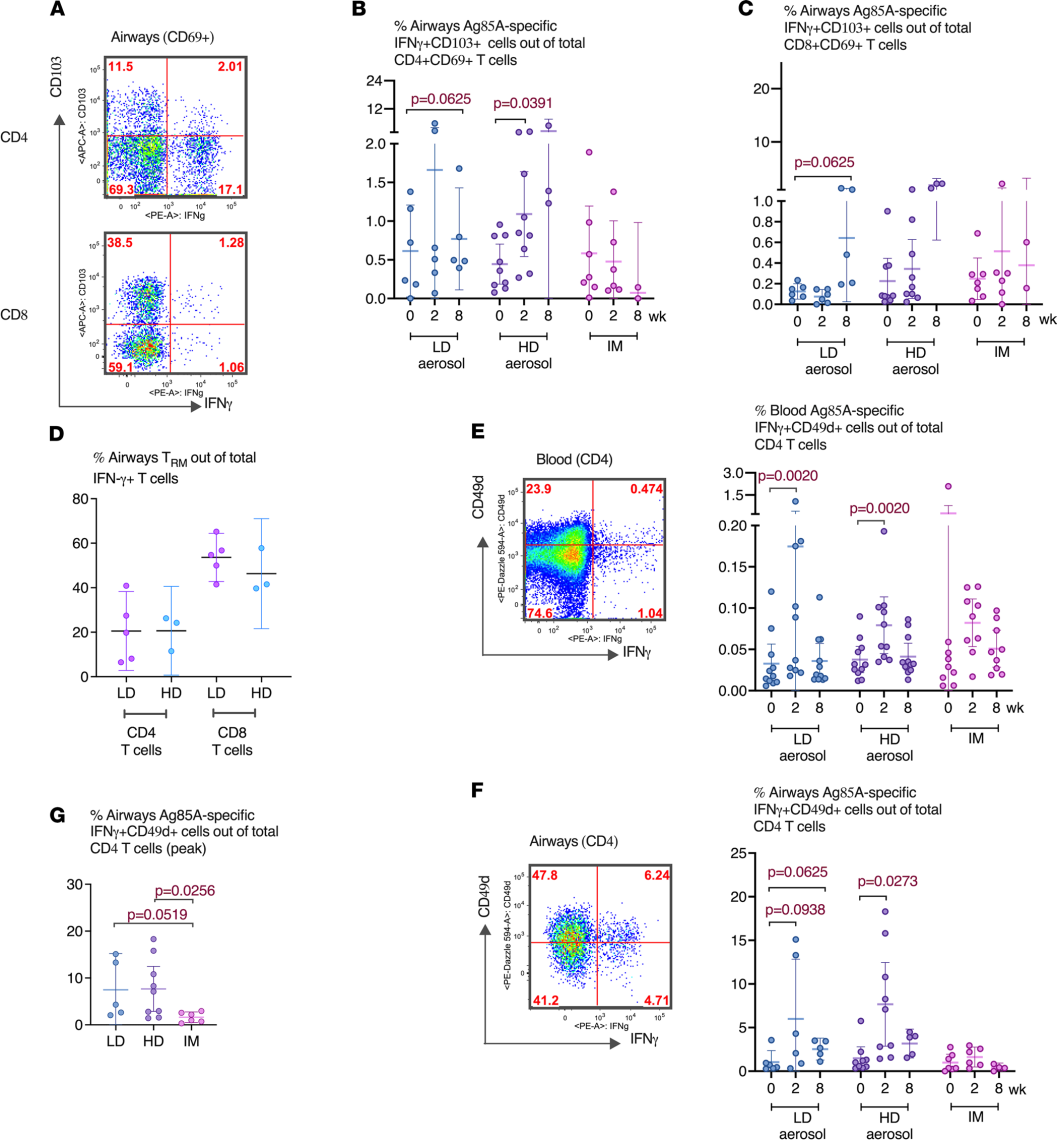
Induction of airway tissue T_RM_ following aerosol or i.m. vaccination. (**A**) Representative dot plots of airway antigen–specific CD4^+^ and CD8^+^ T_RM_ at wk2 in LD aerosol participants. (**B**) Frequencies of airway antigen–specific IFN-γ^+^CD4^+^ T_RM_ coexpressing CD69 and CD103 surface markers at various time points in LD aerosol, HD aerosol, and i.m. vaccine cohorts. (**C**) Frequencies of airway antigen–specific IFN-γ^+^CD8^+^ T_RM_ coexpressing CD69 and CD103 at various time points in LD aerosol, HD aerosol, and i.m. vaccine cohorts. (**D**) Comparison of frequencies of airway antigen–specific CD4^+^ and CD8^+^ T_RM_ coexpressing CD69 and CD103 at 8 weeks after LD and HD aerosol vaccination. (**E**) Representative dot plots of peripheral blood antigen–specific IFN-γ^+^CD4^+^ T cells expressing CD49d at wk2 from LD aerosol participants, and frequencies of circulating antigen-specific CD4^+^ T cells expressing CD49d at various time points in LD aerosol, HD aerosol, and i.m. vaccine cohorts. (**F**) Representative dot plots of airway antigen–specific IFN-γ^+^CD4^+^ T cells expressing CD49d at wk2 from LD aerosol participants, and frequencies of airway antigen–specific CD4^+^ T cells expressing CD49d at various time points in LD aerosol, HD aerosol, and i.m. vaccine cohorts. (**G**) Comparison of frequencies of airway antigen–specific IFN-γ^+^CD4^+^ T cells coexpressing CD49d at the peak time point in LD aerosol, HD aerosol, and i.m. vaccine cohorts. Data in dot plots are expressed as the mean value (horizontal line) with 95% CI. Wilcoxon matched pairs signed-rank test (**B**, **C**, **E**, and **F**) was used to compare various time points with baseline values within the same vaccination group. Mann-Whitney *U* test (**D** and **G**) was used when comparing between vaccination groups.

Lung tissue T_RM_ are critical to protective mucosal immunity (17). Hence, we next determined whether antigen-specific T cells induced by aerosol AdHu5Ag85A vaccination were of tissue-resident memory phenotype and compared them with those induced by i.m. vaccination. BALF cells obtained before and at select time points after vaccination were stimulated with Ag85A p. pool and immunostained for coexpression of 2 key T_RM_ surface markers CD69 and CD103 by antigen-specific IFN-γ–producing CD4^+^ or CD8^+^ T cells (Figure 4A). Marked increases in Ag85A-specific IFN-γ^+^ CD4^+^ and CD8^+^ T cells coexpressing CD69 and CD103 were seen only in the airway of LD and HD aerosol vaccine groups and not in i.m. group (Figure 4, B and C). Although T_RM_ increases at 8 weeks after aerosol vaccination, compared with the baseline, were only marginally statistically significant (95% CI) probably due to small sample sizes, remarkable proportions of Ag85A-specific CD4 T cells (~20%) and CD8^+^ T cells (~54%) present in the airways of aerosol vaccine groups were T_RM_ (Figure 4D). As expected, there was no detectable antigen-specific T_RM_ in the peripheral blood before and after vaccination.

We also studied T cell surface expression of α4β1 integrin (VLA-4; or CD49d for α4), known to be expressed on memory CD4^+^ T cells in human airways (18). Since CD49d may be involved in the homing of circulating T cells to the airway, we first examined CD49d expression on Ag85A-specific CD4^+^ T cells in the circulation. There were small but significantly increased frequencies of circulating CD49d-expressing IFN-γ^+^CD4^+^ T cells, particularly at 2 weeks following LD or HD aerosol vaccination (Figure 4E). In comparison, there were much greater frequencies of CD49d-expressing IFN-γ^+^CD4^+^ T cells (out of total CD4^+^ T cells) in the airways induced by aerosol vaccination (Figure 4F), compared with their frequencies in the circulation (Figure 4E) and in contrast with the lack of such T cells in the airways of i.m. group (Figure 4G). In fact, the majority of Ag85A-specific CD4^+^ T cells in the airways of LD and HD groups expressed CD49d (57% and 74%, respectively). The data indicate that aerosol AdHu5Ag85A vaccination, but not i.m. route of vaccination, is uniquely capable of inducing antigen-specific T cells in the airways endowed with respiratory mucosal homing and T_RM_ properties.

**Figure 5:**
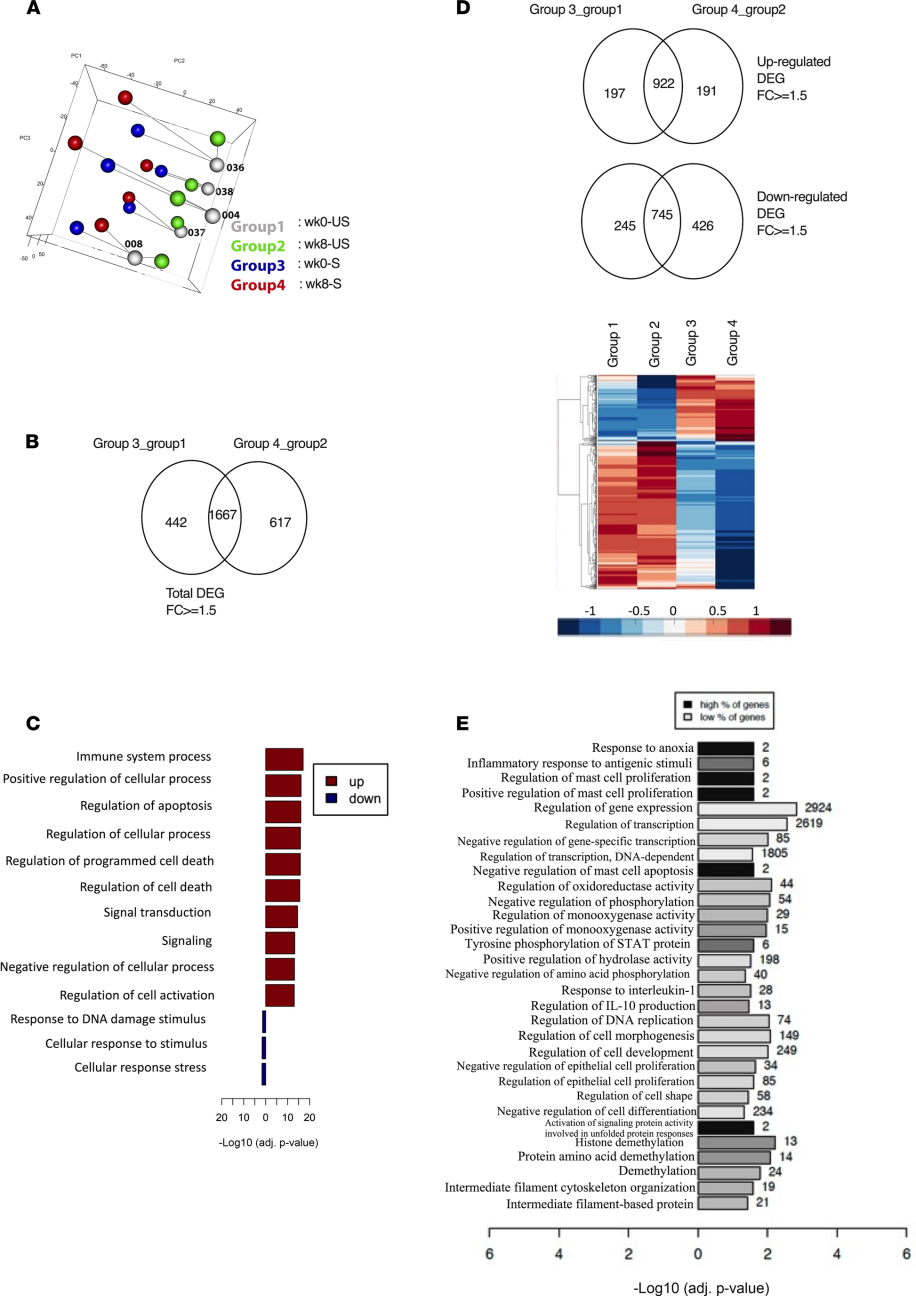
Transcriptomic analysis of alveolar macrophages (AM) following LD aerosol vaccination. (**A**) Principal component analysis (PCA) of gene expression in AM obtained before (wk0) and after (wk8) LD aerosol vaccination cultured with (S) or without (US) stimulation. (**B**) Venn diagram comparing all DEGs in pairwise comparison. (**C**) Significantly enriched functional categories of biological processes by GO associated with DEGs shared between the baseline (wk0) Group 3/1 and aerosol vaccine (wk8) Group 4/2. (**D**) Venn diagram comparing up- and downregulated DEGs in pairwise comparison. Heatmap shows DEG uniquely up- and downregulated, in stimulated aerosol (Group 4) AM. (**E**) Significantly enriched functional categories of biological processes by GO associated with uniquely upregulated DEGs in stimulated aerosol (Group 4) AM. Statistical differences in functional categories of biological processes was performed using BINGO plugin, which uses a hypergeometric test with Benjamini-Hochberg FDR correction.

Since, besides mucosal adaptive immunity, respiratory delivery of AdHu5Ag85A vaccine in experimental animals induced a trained phenotype in airway macrophages (6, 19), we examined whether aerosol vaccination could also alter the immune property of human alveolar macrophages (AM). To this end, we elected to examine the transcriptomics of BALF cells obtained from 5 participants before (week 0 [wk0]) and after (week 8 [wk8]) LD aerosol vaccination. Before RNA isolation, the cells, upon revival from frozen stock, were enriched for AM and cultured with or without stimulation with *M. tuberculosis* lysates and transcriptionally profiled by RNA-Seq analysis. Principal component analysis (PCA) revealed that unstimulated and stimulated AM populations were separated away from each other (Figure 5A). We then identified the differentially expressed genes (DEGs) by comparing wk0-stimulated (Group 3–stimulated) and wk8-stimulated (Group 4–stimulated) AM with respective unstimulated AM (wk0/Group 1) and wk8/Group 2). A total of 2726 genes was differentially expressed upon stimulation in pairwise analysis, of which 1667 genes (61%) were shared between the baseline (wk0) Group 3/Group 1 and aerosol vaccine (wk8) Group 4/Group 2 (Figure 5B). As expected, the shared genes were significantly enriched in biological processes associated with immune response and regulation of cell death (Figure 5C). Furthermore, by pairwise analysis, we identified 191 and 426 genes uniquely upregulated and downregulated, respectively, in stimulated aerosol (Group 4) AM (Figure 5D). The uniquely upregulated genes in stimulated wk8 aerosol AM showed enrichment in a number of biological processes including response to anoxia (OXTR, CTGF), inflammatory response to antigenic stimuli (IL-2RA, IL-1B, IL-20RB), tyrosine phosphorylation of STAT protein (IFN-γ, F2R, OSM), regulation of IL-10 production (CD83, IRF4, IL-20RB, IDO1), response to IL-1 (RIPK2, SRC, IRAK2, IL-1R1, XYLT1, RELA), and histone demethylation (KDM6B, KDM5B, KDM1A, KDM7A, JMJD6; Figure 5E). In comparison, the uniquely downregulated genes in wk8 aerosol AM did not appear significantly enriched for any biological processes. These data suggest that LD aerosol vaccination leads to persisting transcriptional changes in airway-resident AM poised for defense responses.

### Both aerosol and i.m. vaccination induce systemic Th1 responses.

**Figure 6:**
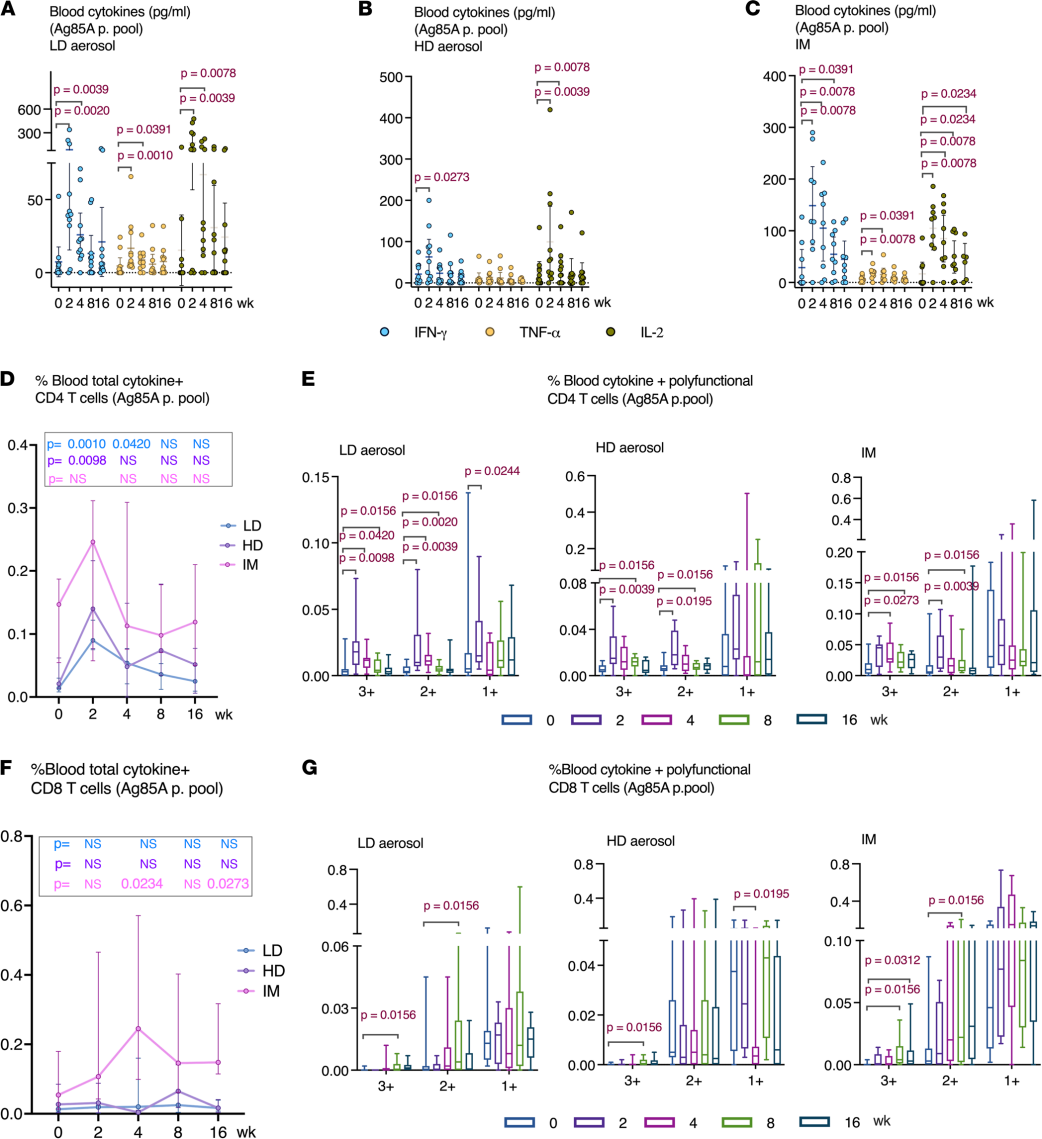
Induction of antigen-specific T cell responses in the peripheral blood following aerosol or intramuscular vaccination. (**A**–**C**) Antigen-specific cytokine production in whole blood culture at various time points after LD aerosol, HD aerosol, and i.m. vaccine groups. The measurements were subtracted from unstimulated control values. (**D**) Frequencies of peripheral blood antigen–specific combined total-cytokine-producing CD4^+^ T cells at various time points in LD aerosol, HD aerosol, and i.m. cohorts. (**E**) Frequencies of peripheral blood polyfunctional (triple/3+, double/2+, and single/1+ cytokine^+^) antigen-specific CD4^+^ T cells at various time points in LD aerosol, HD aerosol, and i.m. vaccine groups. (**F**) Frequencies of peripheral blood antigen–specific combined total-cytokine–producing CD8^+^ T cells at various time points in LD aerosol, HD aerosol, and i.m. groups. (**G**) Frequencies of peripheral blood polyfunctional (triple/3+, double/2+ and single/1+ cytokine^+^) antigen-specific CD8^+^ T cells at various time points in LD aerosol, HD aerosol, and i.m. groups. Data in dot plots are expressed as the mean value (horizontal line) with 95% CI. Box plots show mean value (horizontal line) with 95% CI (whiskers), and boxes extend from the 25th to 75th percentiles. Line graphs show median with IQR. Wilcoxon matched pairs signed-rank test (**A**, **B**, **E**, and **G**) was used to compare various time points with baseline values within the same vaccination group. Mann-Whitney *U* test (**D** and **F**) was used when comparing vaccination groups.

Assessment of overall antigen-specific reactivity of T cells in the circulation before and after vaccination by using whole blood samples incubated with Ag85A peptides indicated that both aerosol, particularly LD aerosol, and i.m. AdHu5Ag85A vaccination induced significant systemic immune responses, as shown by raised IFN-γ, TNF-α, and IL-2 levels in plasma (Figure 6, A–C). AUC analysis, which reflects the overall magnitude of responses, did not differ between LD aerosol and i.m. groups in cytokine production in response to Ag85A p. pool stimulation (IFN-γ, *P =* 0.0910; TNF-α, *P =* 0.6207; IL-2, *P =* 0.8703). However, i.m. vaccine–induced systemic T cell responses appeared to remain significantly increased over a longer duration (Figure 6C). In comparison, the HD aerosol group had significantly lower IFN-γ production than i.m. group (AUC compared with i.m., *P =* 0.0117) whereas they did not differ from each other in the production of TNF-α and IL-2 (Figure 6, B and C).

Further examination of relative activation of CD4^+^ and CD8^+^ T cells by aerosol and i.m. vaccinations using intracellular cytokine staining (ICS) revealed that, compared with the respective baseline, aerosol vaccination activated the circulating Ag85A-specific CD4^+^ T cells to significant levels, while i.m. vaccination moderately increased such responses (Figure 6D). However, the overall magnitude of responses did not differ significantly between aerosol and i.m. groups (AUC: LD aerosol, *P =* 0.1961; HD aerosol, *P =* 0.3545 compared with i.m.). Both LD/HD aerosol and i.m. vaccination also significantly increased Ag85A-specific polyfunctional CD4^+^ T cells coexpressing 3 (3+) or any 2 (2+) cytokines in the circulation (Figure 6E). Consistent with the airway Ag85A-specific CD4^+^ T cell responses (Figure 3, A–C) in both LD and HD aerosol groups, circulating polyfunctional CD4^+^ T cells also generally peaked at 2 weeks after vaccination and remained significantly increased up to 8 weeks (Figure 6E). In comparison, 3+ polyfunctional CD4^+^ T cells in the i.m. group significantly increased at 4 weeks and remained increased up to 8 weeks (Figure 6E). The overall magnitude of circulating 3+ polyfunctional CD4^+^ T cells in the i.m. group was, however, significantly higher than those in aerosol groups (AUC: LD aerosol, *P =* 0.0144; HD aerosol, *P =* 0.0393 compared with i.m.). Circulating 2+ polyfunctional CD4^+^ T cells did not differ significantly between these groups (AUC not significantly different). Consistent with our previous observation (8), besides its activating effects on circulating CD4^+^ T cells, i.m. vaccination also significantly increased Ag85A-specific CD8^+^ T cells up to 16 weeks (Figure 6F). By comparison, aerosol vaccination minimally induced such CD8^+^ T cells in the circulation (Figure 6F). Compared with circulating CD4^+^ T cells (Figure 6E), similar to the overall kinetics of total-cytokine^+^ CD8^+^ T cells (Figure 6F), circulating Ag85A-specific polyfunctional CD8^+^ T cells peaked behind the peak CD4^+^ T cell responses in all vaccine groups (Figure 6G).

The kinetics of polyfunctional profiles of circulating CD4^+^ T cells were further examined in greater detail with a focus on the LD aerosol vaccine group and its comparison with the i.m. group. There existed considerable differences in the polyfunctional profile of circulating Ag85A-specific CD4^+^ T cells between LD aerosol and i.m. groups (Supplemental Figure 4A). In the LD aerosol group, the proportion of IFN-γ^+^TNF-α^+^IL-2^+^ progressively shrank, and at 16 weeks, approximately 75% of the population were TNF-α^+^IL-2^+^ and IFN-γ^+^TNF-α^+^ together with single TNF-α^+^ CD4^+^ T cells. In comparison, in the i.m. group, the proportion of IFN-γ^+^TNF-α^+^IL-2^+^ progressively expanded, constituting approximately 75% of the population at 16 weeks (Supplemental Figure 4A).

Upon examination of circulating BCG-specific CD4^+^ T cells (reactive to *M. tuberculosis*CF^+^ rAg85A stimulation), we found that they were not strikingly increased in aerosol and i.m. vaccine groups, although the trend was higher in i.m. group (Supplemental Figure 4, B and C), and AUC values did not differ significantly between aerosol and i.m. groups (LD aerosol, *P =* 0.0870; HD aerosol, *P =* 0.2666, compared with i.m.). However, LD and HD aerosol vaccination had a significant enhancing effect on the polyfunctionality of preexisting circulating BCG-specific CD4^+^ T cells (Supplemental Figure 4C). Similar to BCG-specific circulating CD4^+^ T cells (Supplemental Figure 4B), BCG-specific circulating CD8^+^ T cells were not significantly increased by either aerosol or i.m. vaccination (Supplemental Figure 4D).

These data indicate that, besides markedly induced mucosal T cell immunity (Figures 3 and 4), respiratory mucosal vaccination via inhaled aerosol, particularly LD aerosol, can also induce systemic polyfunctional CD4^+^ T cell responses, similar to i.m. route of vaccination in previously BCG-vaccinated humans.

### Preexisting and vaccine-induced anti-AdHu5 Ab in the circulation and airways.

The high prevalence of circulating preexisting antibodies (Ab) against AdHu5 in human populations may negatively impact the potency of AdHu5-vectored vaccines following i.m. administration (20). However, little is known about its effect on the potency of AdHu5-vectored vaccine delivered via the respiratory mucosa. To address this question, we first examined the levels of AdHu5-specific total IgG in the circulation and airways (BALF) before and after vaccination (wk0 versus wk4 in circulation; wk0 versus wk8 in BALF). In keeping with our previous findings (8), there were significant levels of preexisting circulating AdHu5-specific total IgG in most of the trial participants (1 × 10^4^ to 1 × 10^5^), and the levels were comparable between the groups (using Kruskal-Wallis test *P =* 0.2048; Table 4). These titres significantly increased after HD aerosol or i.m. AdHu5Ag85A vaccination but not after LD aerosol vaccination. In comparison, preexisting levels of anti-AdHu5 total IgG in the airways were 1 to 1.5 log less than the levels in the circulation and were comparable between groups (using Kruskal-Wallis test, *P =* 0.2048). Of interest, LD and HD aerosol, as well as i.m. vaccination, did not alter the preexisting anti-AdHu5 total IgG levels in the airways (Table 4), but the data from HD aerosol and i.m. groups should be interpreted with caution due to the small sample size at 8 weeks.

Because the total anti-AdHu5 Ab titres may not always correlate with AdHu5-neutralizing capacity in the circulation (8), we further assessed the AdHu5-neutralizing Ab (nAb) titres before and after vaccination in the circulation and airways by using a bioassay. The preexisting AdHu5 nAb titres in the circulation were comparable between groups (using Kruskal-Wallis test, *P =* 0.3588) with 27%, 54%, and 66% of participants in LD, HD, and i.m. groups having > 1 × 10^2^ AdHu5 nAb titres, respectively. Of interest, while i.m. vaccination with AdHu5Ag85A significantly increased the circulating AdHu5 nAb titers by an average of 1.5 logs, LD or HD aerosol vaccination had no such effect (Table 4). On the other hand, similar to total anti-AdHu5 IgG levels, preexisting AdHu5 nAb titers in the airways were ~1 log less than those in the circulation (Table 4). Of importance, 63%, 36%, and 33% of participants in LD, HD., and i.m. groups, respectively, had no detectable baseline AdHu5 nAb titers in their airways, which remained unaltered following vaccination (Table 4). We further found a significant positive correlation between AdHu5 nAb and total AdHu5 IgG titres both in the circulation and airways (Supplemental Figure 5, A and B).

Given that many of the trial participants had moderate to significant levels of AdHu5 nAb titers in the circulation and ~50% of them also had a small but detectable level of preexisting AdHu5 nAb titres in the airways, we next examined whether such nAbs present in the airways and blood may have negatively impacted the immunopotency of LD aerosol and i.m. vaccination, respectively. To this end, the percentage of airways or blood with total-cytokine^+^ Ag85A-specific CD4^+^ T cells at the peak response time (2 weeks post-vaccination) for individual participants was plotted against corresponding preexisting AdHu5 nAb titres, and Spearman rank correlation test was performed. There was no significant correlation between preexisting airways AdHu5 nAb titers and the magnitude of vaccine-induced CD4^+^ (Supplemental Figure 5C) and CD8^+^ (Supplemental Figure 5D) T cell responses in the airways following LD aerosol vaccination. Of note, one participant who hardly responded to aerosol vaccine did have the highest neutralization titers in the cohort (Supplemental Figure 5, C and D). On the other hand, consistent with our previous observation (8), there was no significant correlation between preexisting circulating AdHu5 nAb titers and the magnitude of antigen-specific CD4^+^ (Supplemental Figure 5E) and CD8^+^ (Supplemental Figure 5F) T cell responses in the blood following i.m. vaccination. The above data suggest that, while there is high prevalence of preexisting circulating anti-AdHu5 nAb in humans enrolled in our study, most trial participants have either undetectable or very low levels of preexisting anti-AdHu5 nAb titers in the airways. I.m. AdHu5Ag85A vaccination increases AdHu5 nAb titers in the circulation, whereas aerosol vaccination does not do so either in the airways or in the circulation. Although the presence of AdHu5 nAb in the airways does not seem to have a significant impact on aerosol vaccine immunogenicity, the data should be interpreted with caution due to the small sample size and very few BALF samples with significant AdHu5 nAb titers.

## Discussion

This represents the first clinical study to have fully characterized the method to deliver aerosolized Ad-vectored vaccine to human lungs and to demonstrate its superiority in inducing respiratory mucosal immunity over the i.m. injection.

Both low and high aerosol doses of AdHu5Ag85A were safe and well tolerated. Respiratory adverse events were rare, mild, transient, and similar among groups, and the respiratory functions determined by FEV_1_ and FVC were within normal limits. Systemic adverse events were also similarly rare, mild, and transient. There were no grade 3 or 4 adverse events reported, nor any serious adverse events. There were no clinically significant abnormalities of routine laboratory tests at weeks 2, 4, and 12 following either aerosol or i.m. vaccination.

Our finding that respiratory mucosal vaccination via inhaled aerosol delivery— but not the parenteral i.m. vaccination— is capable of induction of robust respiratory mucosal immunity in human lungs is consistent with the well-established knowledge from animal models of TB and other respiratory infections (3, 21–23). While both the low and high aerosol doses were found to be safe, our findings support the LD aerosol, rather than the HD, to be optimal, as it led to more consistently increased T cell responses in the airway. Although the reasons the LD was more consistently immunogenic than the HD still remain unclear, we hypothesize that it is due to the unique respiratory mucosal immune environment and immune overactivation by the HD aerosol. Of note, this low aerosol dose (1 × 10^6^ PFU) was 100 times smaller than the i.m. dose (1 × 10^8^ PFU) in our study. The remarkable immunogenicity of a rather small dose of a AdHu5-vectored vaccine at human respiratory mucosa was observed, despite the common knowledge that the majority of humans have varying degrees of preexisting Ab against AdHu5 (20, 24). The superiority of AdHu5-vectored vaccine delivered via aerosol, as opposed to i.m. injection, in inducing respiratory mucosal immunity suggests the respiratory tract to be an immune privileged site in terms of anti-AdHu5 nAb. Indeed, we find that approximately 50% of our trial participants have no detectable preexisting nAb titers, and the rest of them have rather low preexisting nAb titers in the airways, contrasting to readily measurable preexisting nAb titers in the blood of most of the trial participants. Such compartmentalized prevalence of anti-AdHu5 Ab in the circulation, but not in the airways of humans, is also reported in a previous study (25). Consideration of the relative prevalence of anti–human Ad nAb is important to the design of human Ad–based vaccine strategies (14, 20, 26). In this regard, although — probably due to a relatively small sample size — we did not find a significant negative correlation between AdHu5 nAb titers and T cell responses in the peripheral blood following i.m. injection of AdHu5Ag85A vaccine, a strong negative correlation has been seen in larger clinical trials with AdHu5-based vaccines (27, 28). Also, for the first time to our knowledge, we find that aerosol delivery of an AdHu5-vectored vaccine did not enhance AdHu5 nAb in the airways, whereas the i.m. route of vaccination significantly enhanced nAb titers in the blood. This represents an additional advantage of inhaled aerosol delivery of Ad-vectored vaccine, suggesting the feasibility of repeated aerosol deliveries of such vaccines for undiminished immunopotency. Furthermore, an increased risk of HIV acquisition was observed paradoxically among clinical trial participants following i.m. injection of an AdHu5-vectored HIV vaccine (29). Although it remains to be investigated, we speculate that aerosol delivery of AdHu5-based vaccine is unlikely to lead to the same outcome. Thus, our findings together suggest that, besides its unique strength and advantage in inducing much-desired respiratory mucosal immunity and repeatability, the inhaled aerosol Ad–based vaccine strategy is free of pain and needles and requires only a small dose to be effective, helping with global vaccine distribution when vaccine supplies may be limited.

Pulmonary TB has remained a major global health issue. Despite great strides made in the past couple of decades to develop improved TB vaccine strategies, BCG — a century-old TB vaccine — continues to be used worldwide (30). To effectively control the global TB epidemic, new, unconventional vaccine approaches, such as respiratory mucosal vaccine strategies, are likely required (2, 3, 5, 22). Respiratory mucosal immunization strategies with appropriately chosen vaccine platforms are adept at inducing the all-round protective immunity consisting of humoral immunity, tissue T_RM_, and trained innate immunity (3). The scientists at Jenner Institute of Oxford University (Oxford, United Kingdom) pioneered an inhaled aerosol method to deliver an MVA-vectored TB vaccine expressing Ag85A to the human respiratory tract and found much-improved T cell responses in the airways over that by intradermal injection (10, 12). Of note, a different aerosol device was used, and the aerosol particles and delivery efficiency were not characterized in these studies. Furthermore, since in these studies, bronchoscopy was performed only once to assess the local mucosal immune responses at 1 week after vaccination, it is unclear whether aerosol vaccine–activated effector T cells may persist, becoming long-term memory T cells in the airways. In comparison, in our current study, not only were the aerosol droplets and delivery efficiency fully characterized, but bronchoscopy was performed before vaccination and twice at 2 and 8 weeks after vaccination. Since the airways of previously BCG-vaccinated and PPD-positive humans — as shown by us in the current study and by others (31), respectively — may harbor mycobacterial antigen–specific T cells at baseline, we believe that bronchoscopy carried out before and after vaccination is needed to help differentiate the authenticity of vaccine-induced T cell responses. While both AdHu5Ag85A and MVA85A vaccines represent the first TB vaccine candidates that have now been successfully tested as inhaled aerosol in human trials, these 2 viral-vectored vaccines express only 1 immunodominant TB antigen (Ag85A). It is likely that vaccines expressing more than 1 TB antigen may provide even greater protective mucosal immunity. In this regard, aerosolized BCG vaccine has demonstrated improved efficacy in nonhuman primates (23). Nevertheless, our current study and those from others (10, 12) have offered an important clinical proof of concept and technological foundation for the further development of respiratory mucosal TB vaccine strategies.

Based on immune analysis of the cells harvested via 8-week postaerosol bronchoscopy, our study offers the critical evidence that an inhaled aerosol, but not an i.m. injected, viral-vectored vaccine can induce persisting tissue T_RM_ within human respiratory tract. Such T cells have been shown to play a critical protective role in respiratory mucosal immunity against pulmonary TB (17, 32). Since tissue T_RM_ are not present in the circulation, the immune analysis of the peripheral blood samples implemented in almost all of the clinical vaccine trials is unable to inform the mucosal immunity related to such T cells (32). Using RNA-Seq, our study also reveals the lasting transcriptional changes in airway macrophages, indicative of trained innate immunity, following aerosol vaccination. We have recently reported that, in preclinical models, respiratory mucosal delivery of AdHu5-vectored TB vaccine induces lasting memory airway macrophages capable of trained innate immunity against *M. tuberculosis* and unrelated bacterial pathogens (6, 19). Incorporating the ability of a vaccine vector to induce tissue-resident trained innate immunity alongside tissue-resident adaptive immunity into the design of TB vaccine strategies may provide the best possible protection (3, 5). Besides markedly induced mucosal immunity, we further found that inhaled aerosol, particularly LD aerosol, vaccination also induced systemic polyfunctional T cell responses as did i.m. vaccination, in support of previous findings (10, 12). These findings suggest that aerosol TB vaccination can provide both local mucosal and systemic protection. Of note, our AdHu5-vectored TB vaccine induced predominantly a CD4^+^ T cell response, more so in the airway, with a smaller effect on CD8^+^ T cells. A similarly biased response of CD4^+^ Th cells was also recently observed in the peripheral blood of humans following AdHu5-COVID-19 vaccination (28).

The well-characterized inhaled aerosol vaccine technology and its superiority to induce respiratory mucosal immunity demonstrated in our current study also offers a foundation and the important proof of concept for developing the next-generation COVID-19 vaccine strategies. Indeed, the effective global control of COVID-19 via the roll-out of the first-generation vaccines has met with threats from emerging variants of concern, dwindling vaccine-induced immunity, and increasing breakthrough infections (15). Of note, several approved first-generation COVID-19 vaccines are Ad-vectored, but they are all administered via i.m. injection (14). Although a recent study has shown that it is safe and well tolerated to deliver, via inhaled aerosol, an AdHu5-vectored COVID-19 vaccine into human respiratory tract (28), there are a number of critical drawbacks associated with this study. This study did not evaluate the local respiratory mucosal immune responses following aerosol and i.m. vaccination, and it is unclear how much aerosolized biologically active vaccine was deposited into human respiratory tract. Furthermore, in this study, 2 repeated high aerosol doses (up to 2 × 10^10^ vp/dose) were compared with an i.m. prime aerosol boost regimen, leaving the question open whether 2 repeated aerosol doses are scientifically justified or whether it is counterproductive. As we have shown in our current study, the respiratory tract is an immunologically highly conducive site for Ad-vectored immunization, and a rather small single aerosol dose (~3 × 10^7^ vp) is able to induce high frequencies of antigen-specific tissue T_RM_ in the airways. We further show that a relatively small increase in aerosol dose did not translate to greater T cell responses; instead, it caused inconsistent immune responses probably due to immune overactivation.

There are a few limitations or considerations associated with our current study. The size of our trial was limited to 31 participants in total. For both safety and immunogenicity, as well as the effect of preexisting anti-AdHu5 Ab, our new aerosol TB vaccine strategy needs to be evaluated further in larger clinical cohorts and in humans with latent TB. Secondly, while the aerosol and i.m. doses were not the same, with the former being considerably smaller, the safe and effective vaccine doses for these 2 routes of delivery are expected to be different from both safety and immunologic perspectives. The lack of mucosal immunity by i.m. vaccination in humans is consistently observed in our current study and by others (10, 12), and it is unlikely that an i.m. dose 50–100 times smaller would have made a difference. Thirdly, since our study represents a phase I trial, it did not have a placebo arm or empty Ad vector control. Although without an empty Ad vector included, it is difficult to tease apart the role of Ad backbone from that by TB antigen in inducing the phenotype of trained airway macrophages, we have recently shown in preclinical models that TB antigen expressed by the vaccine plays a negligible role (6).

In conclusion, our current study has provided technological details for safely and efficiently delivering aerosolized Ad-vector vaccine into the human respiratory tract and has demonstrated its superiority to induce respiratory mucosal immunity over parenteral i.m. injection. Our study offers the important clinical proof of concept and technical platform for the further development of effective vaccine strategies against not only TB, but also other respiratory infections including COVID-19.

## Methods

### Study design and participants.

This was an open-labeled phase I trial investigating a single recombinant genetic TB vaccine AdHu5Ag85A in healthy subjects with a history of BCG vaccination. The vaccine was administered as a single dose either by inhaled aerosol or by i.m. injection. Study participants were recruited by advertising, approved by the McMaster University Research Ethic Board (REB). BCG (+) status was determined based on patient/parent recall, vaccination records, or birth country where BCG vaccine is routinely administered (Figure 1). All enrolled participants were healthy and had normal baseline hematology, spirometry and diffusing capacity of the lungs for carbon monoxide (DLCO), biochemistry, chest x-ray, and negative serological testing for HIV Ab. Latent *M. tuberculosis* infection was excluded by a negative IFN-γ release assay. Current smokers and ex-smokers who had quit within the last year, people with a history of inhaled recreational drugs, respiratory disease (e.g., asthma or chronic obstructive pulmonary disease [COPD], and pregnant or lactating women were excluded. Following vaccination, participants were asked to record their temperature twice a day at set times for 5 days and kept a diary to record any symptoms they experienced for 14 days. Safety and medical evaluation was performed at baseline and at 48–72 hours, as well as at 2, 4, 8, 12, 16, and 24 weeks after vaccine administration. Adverse events were assessed according to the CTCAE Expanded Common Toxicity Criteria (https://ctep.cancer.gov/protocoldevelopment/electronic_applications/ctc.htm). Immunological evaluation was performed with bronchoalveolar lavage and blood samples. Bronchoscopy was carried out within 1–6 days before planned vaccination and at both 2 and 8 weeks after vaccination. Blood samples were collected at baseline and at 2, 4, 8, and 12 weeks after vaccination. All participants provided written informed consent.

The first cohort of 8 BCG^+^ subjects received 1 × 10^6^ pfu (vp/pfu ratio: 32) AdHu5Ag85 vaccine administered by aerosol (LD). The second cohort consisted of 18 participants randomly allocated to receive either 2 × 10^6^ pfu (HD) by aerosol (*n =* 9) or single i.m. administration of 1 × 10^8^ pfu (i.m.) AdHu5Ag85 vaccine (*n =* 9). For the second cohort, a randomization list was generated containing sequential codes linked to route of study vaccine assignment, either i.m. injection or aerosol (Figure 1). Following a review of the data after the enrolment of the second cohort, the protocol was amended; an additional 3 participants were enrolled in the HD aerosol group, and an additional 3 participants were enrolled in the LD group. The study was not blinded. Each participant served as their own control (before and after vaccination), and there was no placebo group. For safety reasons, 2 participants were first vaccinated for each aerosol dose and were followed for 2 weeks after vaccine administration before immunizing the rest of the participants in the dose group. Reports detailing adverse events and serious adverse events for 4 weeks after dose were reviewed by the safety monitoring committee before administration of the higher dose. All participants were followed for a total of 24 weeks after vaccine administration. Other trial details are included in the trial protocol provided in the supplemental material..

### Aerosol delivery device characterization.

The Aeroneb Solo Micropump was selected as the delivery device (Supplemental Figure 1). The aerosol performance of the Aeroneb Solo Vibrating Mesh Nebulizer with AdHu5Ag85A was characterized using standard procedures and performance metrics (16). FV, delivery time, ED of vaccine aerosol available at the mouth collected on standard filters, aerosol droplet size characteristics using the NGI Cascade Impactor operated at 15 L/min were measured. Salbutamol sulphate served as the tracer for saline droplets containing vaccine particles. Regional deposition of vaccine droplets in the lung was estimated from the particle size metrics of the carrier (salbutamol) aerosol. An indication of the available vaccine dose at the mouth was predicted from ED. The amount of aerosol-containing vaccine estimated to be deposited into the lung was calculated using ED in combination with particle size statistics. Viability of the aerosolized vaccine was determined by plaque-forming assay.

### Vaccine and manufacturing.

Clinical-grade AdHu5Ag85A vaccine was provided by the Robert E. Fitzhenry Vector Laboratory, McMaster Immunology Research Centre, McMaster University. AdHu5Ag85A was produced according to current Good Manufacturing Practices (cGMP) in the Vector Laboratory and has been fully certified. For each participant allocated to receive vaccine by aerosol, a single dose of AdHu5Ag85A diluted in 0.5 mL saline was aerosolized using the Aeroneb Solo and inhaled via mouthpiece using tidal breathing. For participants allocated to receive i.m. route, AdHu5Ag85A diluted in 0.5 mL saline was injected as described previously (8).

### Bronchoalveolar lavage procedure and cell processing.

Bronchoscopy was performed using a flexible bronchoscope, with the procedure performed in the research facility at the Health Science Centre, McMaster University by a trained respiratory physician. Following light sedation (using midazolam and fentanyl) and local anaesthesia to the upper and lower respiratory tracts, the bronchoscope was advanced until wedged in the right middle bronchus and approximately 40 mL of sterile saline instilled and then aspirated back using gentle manual suction in a 50 mL syringe. This was sequentially repeated an additional 3 times with a total of 160 mL of saline lavage. Oxygen saturations were monitored throughout the whole procedure. After the bronchoscopy, vital signs (SaO_2_, heart rate, blood pressure) were measured immediately, 15 minutes, 30 minutes, 45 minutes, 60 minutes, and 2 hours throughout recovery. Spirometry was repeated to ensure FEV_1_ > 70% predicted and within 15% of prebronchoscopy values before discharge. Each aspirate was kept separate on ice and processed within 1 hour after collection at McMaster Immunology Research Center. The first aspirate was discarded after obtaining the cell count. Second, third, and fourth BALFs were saved separately and stored at –80°C for future analysis. Cells were then pooled and counted.

Excess BALF cells were frozen at a concentration of 5 × 10^6^ to 9 × 10^6^ cells/mL in 12.5% HSA/DMSO. Vials were placed into Mr. Frosty (Thermo Fisher Scientific) and stored at –80^0^C overnight and transferred to liquid N_2_ until needed. Before using BAL cells in assays, cryovials were brought to the lab in liquid N_2_, thawed in a 37°C water bath, and transferred into a polypropylene tube. cRPMI media was added to the cells in a stepwise manner as follows. Added 50 μL warmed cRPMI and incubated for 1 minute at room temperature followed by 100, 200, 400 μL warmed cRPMI with 1 minute incubation at room temperature after adding each volume. Centrifugation was done at 370*g* with low brake for 10 minutes at room temperature. Cells were washed 3 times and revived for 6 hours before use in assays. Viability of cells ranged from 70% to 89%.

### QFT assay.

IFN-γ release assay was performed using QuantiFERON TB Gold in tube or QuantiFERON TB Gold Plus (Qiagen) according to manufacturer instructions to determine latent TB status (8).

### ELISA for anti–AdHu5 total IgG and bioassay for AdHu5 nAbs.

Collected serum and concentrated BALF samples (20-fold concentrated from 10 mL of combined aspirates 2 and 3 using Centriprep 3 kDa Cut-off centrifugal filter unit; Millipore-Sigma) were measured for anti–AdHu5 IgG Ab as previously described (8). AdHu5 nAb levels in serum and concentrated BALF were assessed as a function of GFP-expressing AdHu5 infection using A549 cells as previously described (33). For both total IgG and nAb titers, serum samples at a 1:10 and BALF at a 1:5 dilution were used.

### Mucosal and systemic Ag-specific responses assessed by Luminex and ICS.

We counted the total cells in the BALF and calculated the number of cells per mL of BALF. Differential cell count was performed on the cells from BALF using cytospin. We quantified the Ag85A- and *M. tuberculosis* antigen–specific T cell responses in the peripheral blood and in the airways represented by the BALF using Luminex and/or ICS assay as previously described (8). Antigens used for stimulation included Ag85A p. pool and a cocktail of mycobacterial antigens consisting of *M. tuberculosis* culture filtrate proteins (*M. tuberculosis*CF) and recombinant Ag85A protein (rAg85A) at concentrations previously described (8). Unstimulated and PHA-stimulated cells were set up in parallel as background and positive controls. For whole blood culture and the Luminex, 1 mL of heparinized whole blood was added into each well of a 24-well plate. Each was stimulated for 18–24 hours with one of the antigens mentioned above. Collected plasma was stored at −70°C. Cytokines were determined for human IFN-γ, IL-2, and TNF-α using Luminex Multiplex Kit from MilliporeSigma. Fresh PBMC and BALF cells were stimulated with antigens, and wells were processed according to manufacturer’s instruction (BD Biosciences).

For determination of CD4^+^ and CD8^+^ T cell responses to vaccine using the whole blood culture (10), 1 mL of blood was stimulated with each of the antigens mentioned above in the presence of 1 μg/mL αCD28 (clone CD28.2, 5566620, BD Biosciences), 1 μg/mL αCD49d (clone 9F10, 5566634, BD Biosciences). Samples were incubated at 37°C in 5% CO_2_ for 6 hours with Ag85A p. pool or PHA stimulation or for 12 hours with *Mtb*CF-rAg85A stimulation. Brefeldin-A was added for the last 5 hours (Ag85A p. pool or PHA) or the last 6 hours (*Mtb*CF-rAg85A or unstimulated whole blood). At the end of incubation, RBCs were lysed, and samples were frozen in liquid nitrogen until FACS analysis. To determine the CD4^+^ and CD8^+^ T cell responses in the airways, 0.5 × 10^6^ to 1 × 10^6^ fresh BALF cells were plated and stimulated as for the whole blood. At the end of incubation, cells were immediately stained for ICS and FACS analysis.

Surface immunostaining and ICS was done as previously described (8). Briefly, frozen cells from whole blood were thawed and permeabilized before staining with a cocktail of fluorochrome-conjugated monoclonal Ab; CD3 (FITC) (clone OKT3, 317306, BioLegend), CD4 (PB) (clone RPA-T4, 300502, BioLegend), CD8 (PECy7) (clone HIT8a, 300914, BioLegend), IFN-γ (PE) (clone B27 [RUO], 559327, BD Biosciences), IL-2 (APC) (clone 5344.11, 341116, BD Biosciences), and TNF-α (PerCP-Cy5.5) (clone Mab 11, 560579, BD Biosciences). BALF cells were surface-immunostained for viability (Molecular Probes LIVE/DEAD fixable stain [Aqua], Invitrogen), followed by CD4 (AF700) (clone RPA-T4, BD Biosciences), CD14 (V450) (clone MϕP9, BD Biosciences), and CD19 (V450) (clone HIB19, BD Biosciences). Cells were then permeabilized and stained for intracellular cytokines IFN-γ (PE) (clone B27 (RUO), IL-2 (APC) (clone 5344.11), and TNF-α (FITC) (clone Mab 11) together with surface markers CD3 (PerCP-Cy5.5) (clone SKT, BD Biosciences) and CD8 (PECy7) (clone HIT8a). Ag85A p. pool–stimulated whole blood and BALF cells were also stained for surface markers CD103 (APC) (clone Ber-ACT8, 350216, BioLegend), CD69 (PerCP-Cy5.5) (clone FN50, 310926, BioLegend), and CD49d (PE-dazzle) (clone 9F10, 304326, BioLegend) to evaluate the surface expression of tissue T_RM_–associated molecules or T cell lung trafficking–associated molecules. Cells were analyzed with LSRII flow cytometer and assessed with Flowjo version 9.9.6 (Tree Star Inc.).

### Transcriptomic analysis of airway macrophages.

We evaluated the induction of trained innate immunity in airway macrophages in the LD dose group. After reviving the frozen BALF cells for 6 hours, 1 million viable cells were seeded on a 12-well plate and incubated for 2 hours at 37°C, 5% CO_2_. At that point, airway macrophages were enriched by removal of nonadherent cells via extensive washing with prewarmed (37°C) PBS and then cultured with or without *M. tuberculosis* lysates stimulant for 12 hours. Total RNA was extracted using RNeasy Plus Mini Kit from Qiagen, which includes gDNA eliminator columns, following the manufacturer’s protocol. Quality of RNA was verified, and subsequent RNA-Seq was carried out by Farncombe Metagenomic Facility at McMaster University. RNA integrity was checked using the Agilent bioanalyzer. mRNA was converted to cDNA after enrichment. cDNA libraries were sequenced using an Illumina HiSeq machine (1 × 50 bp sequence reads). Data were analyzed as previously described (6). Before the analysis of DEGs, reads were filtered by quality and counted. Genes, showing low levels of expression were removed using EdgeR package in R. Statistical analysis was performed with 11,848 genes. The Limma package in R was used to identify DEGs in stimulated macrophages, and DEGs were then compared with their respective unstimulated controls. Stringent criteria, including log_2_ of fold-change ≥ 1.5 or ≤ 1.5, and corrected *P* <0.05 were applied to filter DEGs. Ontology analysis for the biological processes component was performed using BINGO plugin (https://apps.cytoscape.org/apps/bingo), and bar charts were created using top 10 biological processes shared between the comparisons (reflecting adjusted *P* values averaged between the pairwise comparisons) or using all biological processes unique to the comparison of interest. The data files for RNA sequences and analysis have been deposited in the NCBI Gene Expression Omnibus under accession no. GSE190850.

### Outcomes.

The primary outcome of this trial was safety of a single administration of vaccine delivered to the respiratory tract by aerosol or i.m. injection. The frequency and severity of vaccine-related local and systemic adverse events were collected from participants from a self-completed diary for 14 days after vaccination (fever, chills, cough, wheezing, sneezing, shortness of breath, chest pain, headache, fatigue or malaise, conjunctivitis, rhinitis, epistaxis, injection site reaction, syncope, or light-headedness) and at scheduled follow-up visits.

Routine laboratory biochemical and hematological tests (CBC, sodium, potassium, creatinine, aspartate aminotransferase [AST], bilirubin) were measured at 2, 4, and 12 weeks after vaccination, and lung function (FEV_1_ and FVC) was measured at 2, 4, 8, and 12 weeks after vaccination. The secondary outcome was a comparison of immunogenicity among the different routes and aerosol dose groups.

### Statistics.

Adverse events were reported descriptively. Immunogenicity data were analyzed using Prism (version 9.2.0). Data are expressed as the mean value (horizontal line) with 95% CI. Box plots show mean value (horizontal line) with 95% CI (whiskers), and boxes extend from the 25th to 75th percentiles. Violin plots show the median and quartiles. Pie chart shows median proportions of polyfunctional Ag-specific T cells. Wilcoxon matched pairs signed-rank test was used when comparing change of T cell or cytokine responses at various time points from the baseline values within the same vaccination group (LD aerosol, HD aerosol, and i.m. groups). Mann-Whitney *U* test was used for comparison of the difference in T cell responses between vaccination groups. For correlation analysis, the Spearman rank coefficient test was used. Two-tailed *P* values of less than 0.05 were considered significant.

### Study approval.

This phase I trial was approved by the Health Canada and Hamilton Integrated Research Ethics Board. This trial is registered with ClinicalTrial.gov (NCT02337270). All participants provided written informed consent.

## Author contributions

FS, ZX, GMG, and MJ designed the trial. MBD, MFCM, MRT, ZX, and FS characterized the aerosol device and aerosol droplets. BDL and MFCM took charge of vaccine GMP manufacturing. MJ, DKF, SA, AZ, and ADG performed immunological assays and data analysis. EA, KJH, RFS, RPC, PMO, MK, IS, GMG, and FS contributed to participant recruitment, clinical data management, safety assessment, bronchoscopy, or other clinical assistance. ZX and FS secured the funding. MJ, ZX, and FS wrote the manuscript.

## Supplementary Material

Supplemental data

ICMJE disclosure forms

## Figures and Tables

**Table 1:**
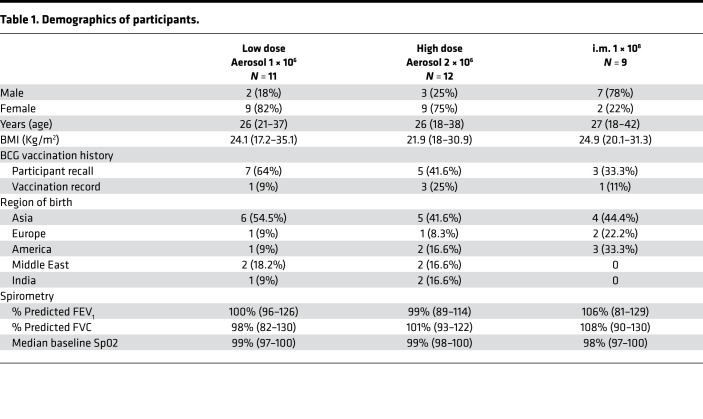
Demographics of participants.

**Table 2:**
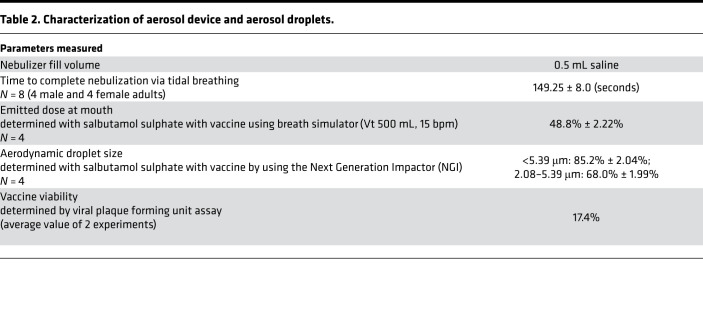
Characterization of aerosol device and aerosol droplets.

**Table 3:**
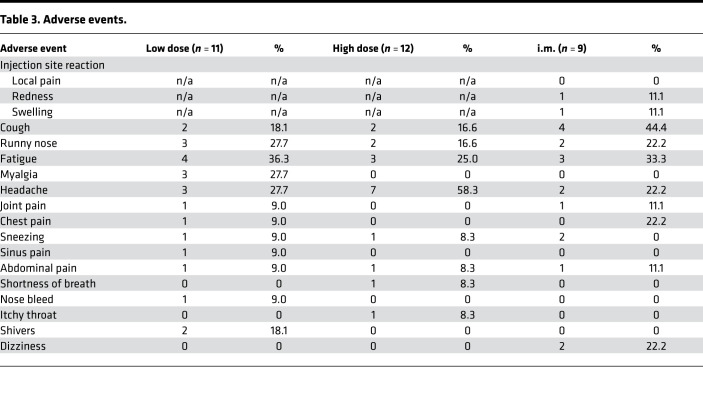
Adverse events.

**Table 4:**
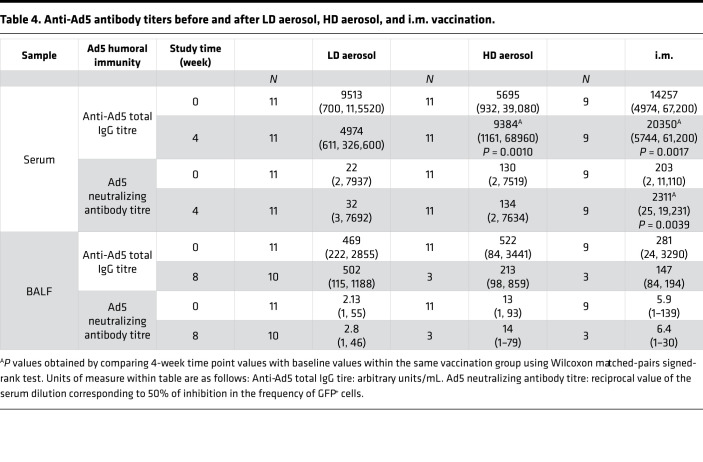
Anti-Ad5 antibody titers before and after LD aerosol, HD aerosol, and i.m. vaccination.

## Version 1 01/06/2022

In-Press Preview

## Version 2 02/08/2022

Electronic publication
