# Supplementary material for: Aerosol delivery, but not intramuscular injection, of adenovirus-vectored tuberculosis vaccine induces respiratory-mucosal immunity in humans
Source: JCI Insight. 2022 Feb 8;7(3):e155655. doi: 10.1172/jci.insight.155655 (PMC8855837; doi:10.1172/jci.insight.155655)
Supplement: Supplemental data [file jciinsight-7-155655-s227.pdf]

Aerosol delivery, but not intramuscular injection, of adenovirus-vectored tuberculosis vaccine induces respiratory-mucosal immunity in humans

Mangalakumari Jeyanathan,[1,2,3] Dominik K. Fritz,[1,2,3] Sam Afkhami,[1,2,3] Emilio Aguirre,[4] Karen J. Howie,[3] Anna Zganiacz,[1,2,3] Anna Dvorkin-Gheva,[1,3] Michael R. Thompson,[5] Richard F. Silver,[6] Ruth P. Cusack,[3] Brian D. Lichty,[1,3] Paul M. O'Byrne,[3] Martin Kolb,[3] Maria Fe C. Medina,[1,3] Myrna B. Dolovich,[3] Imran Satia,[3] Gail M. Gauvreau,[3] Zhou Xing,[1,2,3] & Fiona Smaill[2,4]

[1]McMaster Immunology Research Centre, [2]M. G. DeGroote Institute for Infectious Disease Research, [3]Department of Medicine, [4]Department of Pathology & Molecular Medicine, and [5]Department of Chemical Engineering, McMaster University, Hamilton, Ontario, Canada; [6]Department of Critical Care and Sleep Medicine, Case Western Reserve University School of Medicine, Cleveland, Ohio, USA

Supplemental Figures & Information

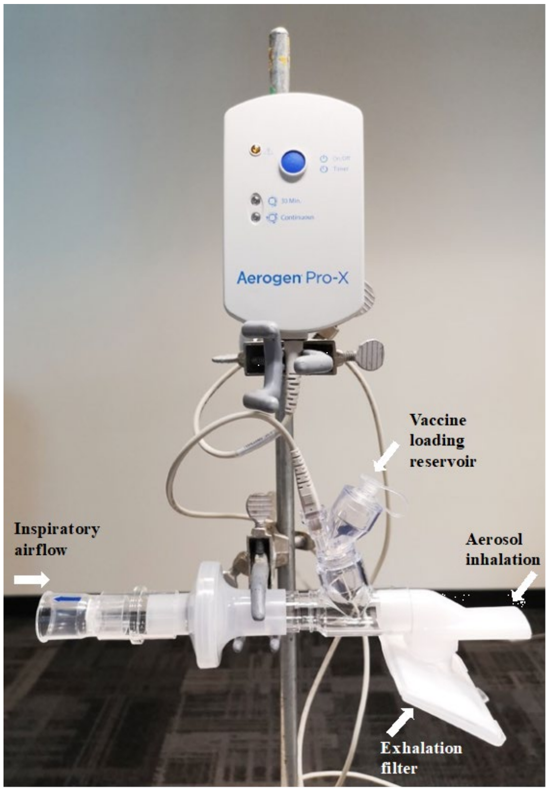

Supplemental Figure 1 Inhaled aerosol delivery device set-up. The Aeroneb® Solo Micropump was used as part of the delivery device set-up.

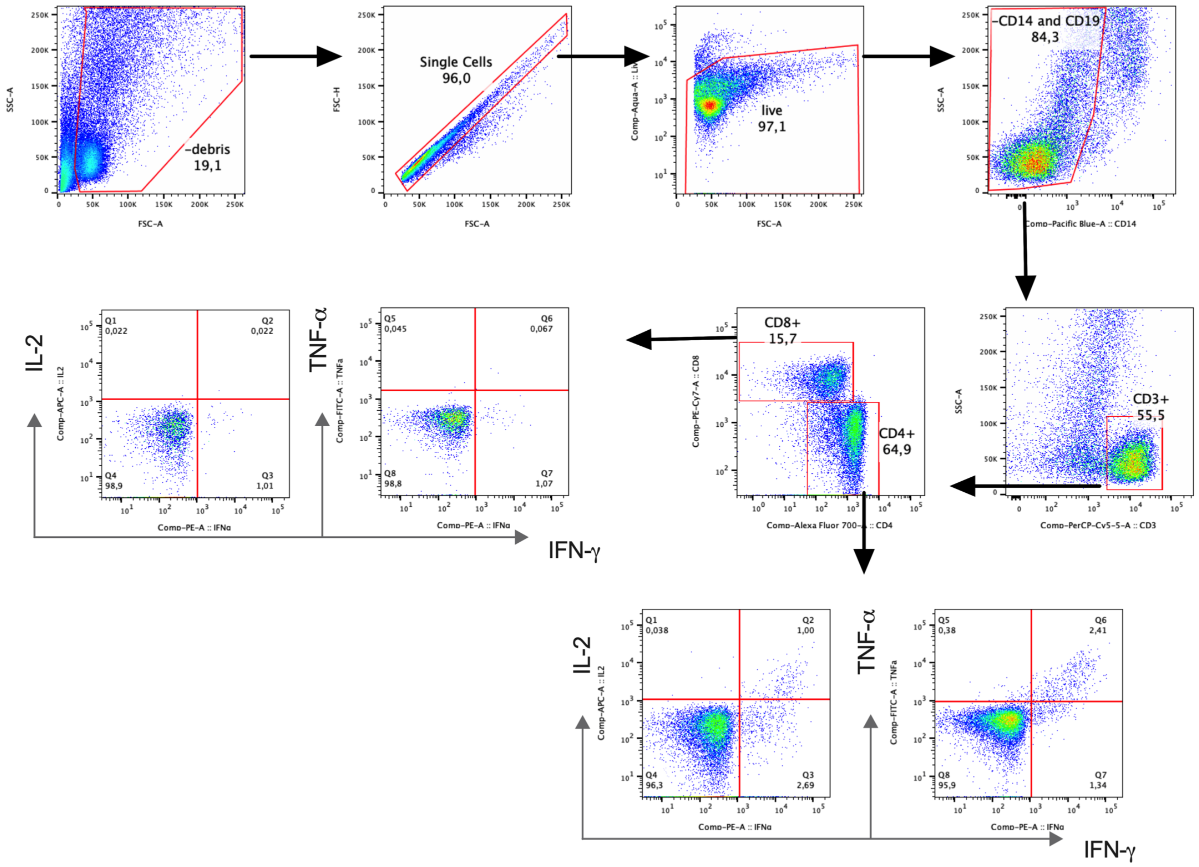

Supplemental Figure 2 Gating strategy used for the assessment of cytokine production by airway CD4 and CD8 T cells. Fresh BALF cells cultured with or without stimulation were immunostained for intracellular production of IFN-g, TNF-a and IL-2. Debris was removed and single cells were then gated to remove dead cells. CD4 and CD8 T cells were gated out of total CD3+ cells after removing myelomonocytic lineage (CD14+) and B cell lineages. Multiple cytokine-producing CD4 and CD8 T cells were identified using Boolean gating. Dotplots shown are representative of BAL cells stimulated with Ag85A single peptide pool at 2 wk post-aerosol vaccination.

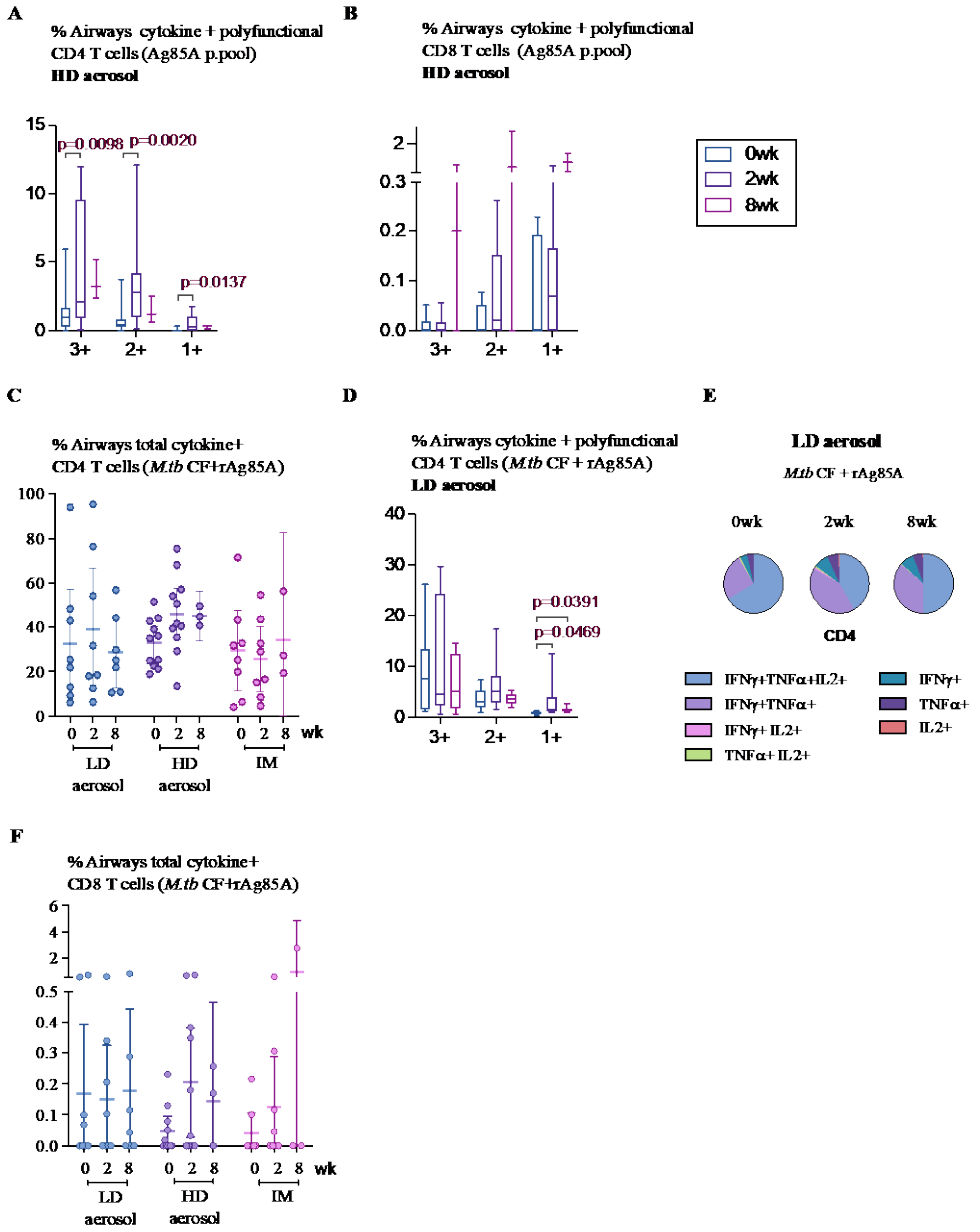

Supplemental Figure 3 Induction of multifunctional T cells specific for Ag85A or a cocktail of mycobacterial antigens in the airways following aerosol or intramuscular vaccination. **A**), **B**) Frequencies of airways Ag85A-specific polyfunctional (triple/3+, double/2+ and single/1 cytokine-positive) CD4 and CD8 T cells at various timepoints in HD aerosol group. **C**) Frequencies of airways combined total cytokine-producing CD4 T cells specific for *M.tb* CF/rAg85A at various timepoints in LD aerosol, HD aerosol and IM cohorts. **D**) Frequencies of airways polyfunctional (triple/3+, double/2+ and single/1 cytokine-positive) CD4 T cells specific for *M.tb* CF/rAg85A at various timepoints in LD aerosol group. **E**) Median proportions displayed in pie chart of *M.tb* CF/rAg85A-specific airways CD4 T cells expressing a specific single or combination of two or three cytokines at various timepoints in LD aerosol group. **F**) Frequencies of airways combined total cytokine-producing CD8 T cells specific for *M.tb* CF/rAg85A at various timepoints in LD aerosol, HD aerosol and IM cohorts.

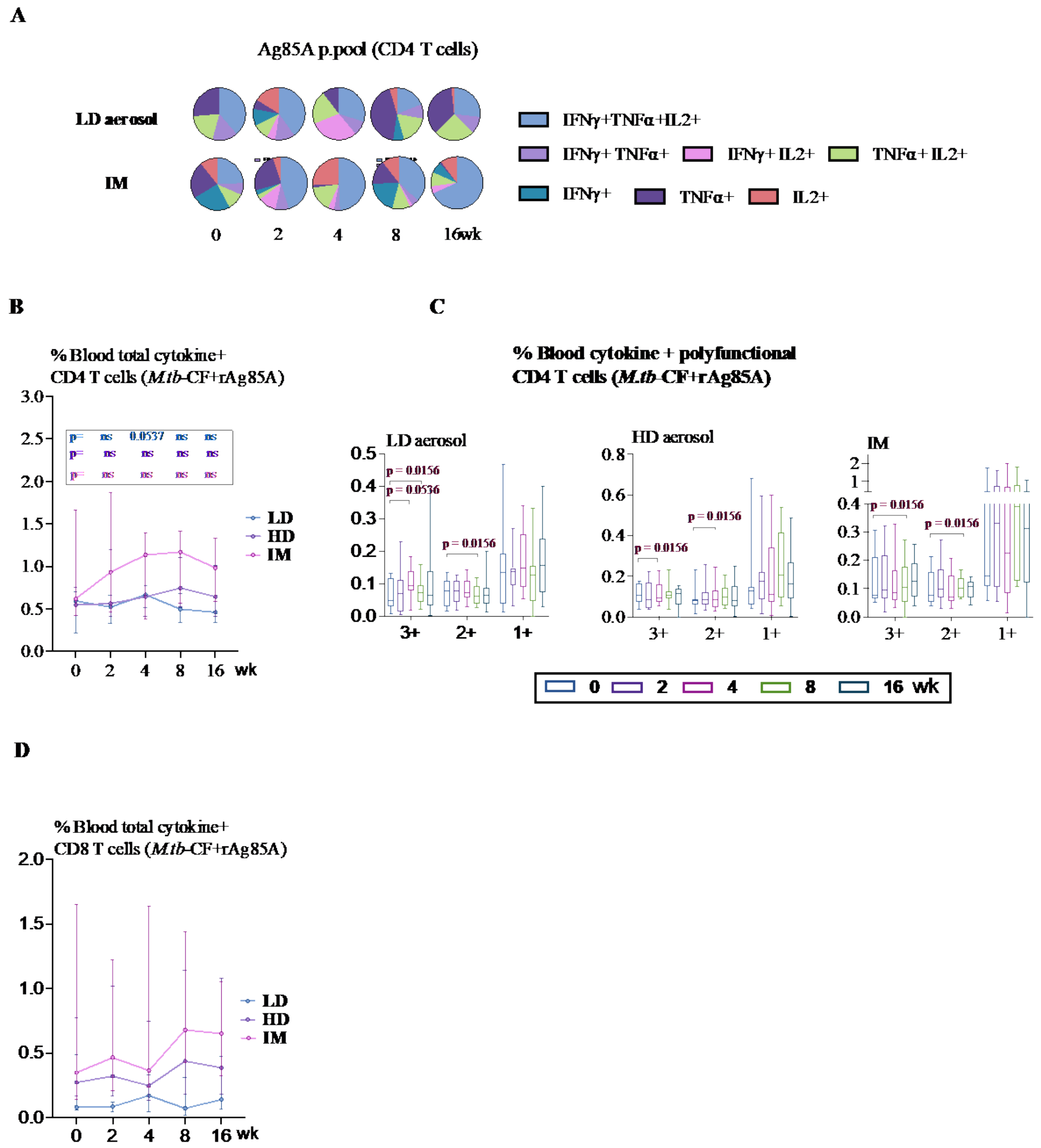

Supplemental Figure 4 Induction of multifunctional T cells specific for Ag85A or a cocktail of mycobacterial antigens in the peripheral blood following aerosol or intramuscular vaccination. **A**) Median proportions displayed in pie chart of peripheral blood Ag85A-specific CD4 T cells expressing a specific single or combination of two or three cytokines at various timepoints in LD aerosol and IM vaccine groups. **B**) Frequencies of peripheral blood *M.tb* CF/rAg85A-specific combined total cytokine-producing CD4 T cells at various timepoints in LD aerosol, HD aerosol and IM cohorts. **C**) Frequencies of peripheral blood *M.tb* CF/rAg85A-specific polyfunctional (triple/3+, double/2+ and single/1 cytokine-positive) CD4 T cells at various timepoints in LD aerosol, HD aerosol and IM vaccine groups. **D**) Frequencies of peripheral blood *M.tb* CF/rAg85A-specific polyfunctional (triple/3+, double/2+ and single/1 cytokine-positive) CD8 T cells at various timepoints in LD aerosol, HD aerosol and IM vaccine groups.

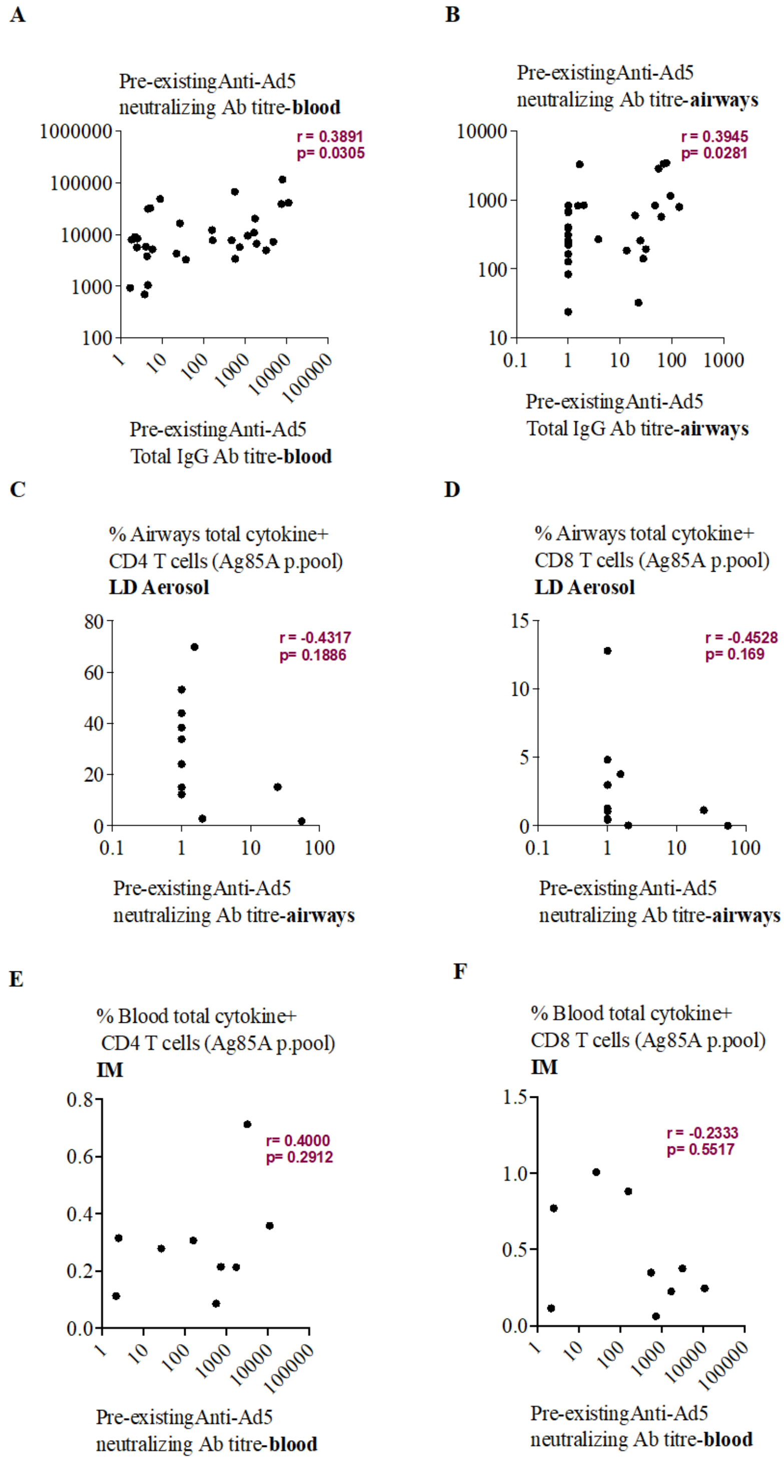

Supplemental Figure 5 Impact of pre-existing anti-AdHu5 antibodies on the immunogenicity induced by LD aerosol and IM AdHu5Ag85A vaccination. **A**) Correlation plot of pre-existing anti-Ad5 neutralizing antibodies versus pre-existing anti-Ad5 total IgG antibody titers in the blood of all trial participants. **B**) Correlation plot of pre-existing anti-Ad5 neutralizing antibodies versus

pre-existing anti-Ad5 total IgG antibody titers in the airways of all trial participants. **C**) Correlation plot of pre-existing anti-Ad5 neutralizing antibodies versus peak airways antigen-specific total cytokine+ CD4 T cell responses in LD aerosol vaccine group. **D**) Correlation plot of pre-existing anti-Ad5 neutralizing antibodies versus peak airways antigen-specific total cytokine+ CD8 T cell responses in LD aerosol vaccine group. **E**) Correlation plot of pre-existing anti-Ad5 neutralizing antibodies versus peak peripheral blood antigen-specific total cytokine+ CD4 T cell responses in IM vaccine group. **F**) Correlation plot of pre-existing anti-Ad5 neutralizing antibodies versus peak peripheral blood antigen-specific total cytokine+ CD8 T cell responses in IM vaccine group.

CLINICAL TRIAL PROTOCOL # M002

Control #163439, File 9427-M2105-82C

PHASE I, OPEN-LABEL CLINICAL TRIAL
TO EVALUATE THE SAFETY AND IMMUNOGENICITY
OF AN ADENOVIRUS-BASED TUBERCULOSIS VACCINE
ADMINISTERED BY AEROSOL

Institute for Infectious Diseases Research
McMaster Immunology Research Centre
Department of Pathology and Molecular Medicine

Type of Study: Phase I

Date of Protocol: 22 August 2014
Version: 1.3
Date of amendment:12 April 2019
Date of amendment: 27 January 2020
Date of amendment: 20 November 2020

PROTOCOL/AMENDMENT APPROVAL PAGE(S)

Authored by:

Dr Fiona Smaill
Printed Name

Signature
905-521-2100 Ext 74190
Telephone Number
smaill@mcmaster.ca
E-mail Address

Date (dd/mmm/yyyy)

Co-Authored by:

Dr Zhou Xing
Printed Name

Signature
905-525-9140 Ext 22354
Telephone Number
xingz@mcmaster.ca
E-mail Address

Date (dd/mmm/yyyy)

Co-Authored by:

Dr Martin Kolb
Printed Name

Signature

Telephone Number

E-mail Address

Date (dd/mmm/yyyy)

Co-Authored by:

Dr Myrna Dolovich
Printed Name

Signature

Telephone Number

E-mail Address

Date (dd/mmm/yyyy)

Co-Authored by:

Dr Maria fe Medina
Printed Name

Signature
905-525-9140 Ext 22946

Co-Authored by:

Dr Paul O'Byrne
Printed Name

Signature

Telephone Number<br>mmedina@mcmaster.ca	Telephone Number
E-mail Address	E-mail Address
Date (dd/mmm/yyyy)	Date (dd/mmm/yyyy)

Co-Authored by:

Dr Marek Smieja	Dr. Gail Gauvreau
Printed Name	Printed Name
Signature	Signature
Telephone Number	Telephone Number
E-mail Address	E-mail Address
Date (dd/mmm/yyyy)	Date (dd/mmm/yyyy)

Approved by:

The undersigned attest to the following:
1. Reviewed the protocol / amendment
2. Agrees to its contents

Dr Fiona Smaill	Dr. Zhou Xing
Printed Name	Printed Name
Signature	Signature
905-521-2100 Ext 74190	905-525-9140 Ext 22354
Telephone Number	Telephone Number
smaill@mcmaster.ca	xingz@mcmaster.ca
E-mail Address	E-mail Address
Date (dd/mmm/yyyy)	Date (dd/mmm/yyyy)

IMPORTANT CONTACTS AT MCMASTER UNIVERSITY MEDICAL CENTRE.

Person responsible for this study:	Name	Telephone extension	E-Mail Address
Principal Investigator	Dr Fiona Smaill	905-521-2100 x 74190	smaill@mcmaster.ca
Co-investigator	Dr Zhou Xing	905-525-9140 x 22354	xingz@mcmaster.ca
Co-investigator	Dr Martin Kolb	905-522-1155 x 33683	kolbm@mcmaster.ca
Research co-ordinator	Emilio Aguirre	1-289-659-4901	aguirre@mcmaster.ca

HISTORY OF AMENDMENTS TO PROTOCOL

Amendment 3

The following table outlines the changes made to the protocol and the affected sections

Change and Rationale for change	Original protocol	Section(s) changed
Version and date updated to:<br>Version 1.3<br>Date of amendment: 20 November 2020	Version 1.2<br>Date of amendment: 27 January 2020	Cover page
Updates to study synopsis as per updates to protocol		Study synopsis
Changed to:<br>*Additional participants will be enrolled … until at least 6 participants have received the higher dose of vaccine by aerosol and completed the 2 and 8 week bronchoscopies.*<br><br>*Enrolment into the IM cohort will be discontinued once at least 9 participants have been randomized to the IM route, received vaccine and completed at a minimum the week 2 bronchoscopy (see Amendment 3 and Appendix 9)*<br><br>*Following completion of enrolment in the higher dose aerosol cohort, an additional three participants (with additional participants enrolled to adjust for participants who do not complete the week 2 and 8 bronchoscopies) will be enrolled into the low dose aerosol cohort (see Amendment 3 and Appendix 9).*<br><br>Rationale: The restrictions to our research program because of the COVID-19 pandemic has significantly disrupted participant enrolment and completion of the protocol-defined bronchoscopies. We therefore plan to modify the enrolment plan to ensure adequate statistical power for comparisons as well as timely completion of the study.<br>In the participants who received IM vaccine (n=9) we have consistently seen a lack of antigen-specific airway cells in the BAL and we are confident that we have studied an adequate number of participants to conclude that the IM route is unable to induce airway T cell responses. We plan to discontinue further enrolment into the IM cohort. Given our observations of the difference in airway peak T cell response rates seen between the low and high dose cohorts thus far, we plan to enroll an additional 3 participants into the low dose group to increase the statistical power of the comparison between the two aerosol doses.	Additional participants will be enrolled … until at least 8 participants have received the higher dose of vaccine by aerosol and completed the 2 and 8 week bronchoscopies.	5.1 and Appendix 9

Changed to:<br>*Between 48 and 72 hours after the administration of the vaccine … will be contacted for follow-up, seen in the clinic if necessary and undergo a physical examination and any new symptoms identified and as necessary managed clinically.*<br><br>Rationale:<br>To date in our participants there have been no concerning adverse events in participants following vaccination. With restrictions due to COVID-19, where appropriate, follow-up visits will be done by telephone and participants only seen if there is a clinical need.	Between 48 and 72 hours after the administration of the vaccine … subjects will be seen for follow-up, and undergo a physical examination and any new symptoms identified and as necessary managed clinically.	

Amendment 2

The following table outlines the changes made to the protocol and the affected sections.

Change and Rationale for change	Original protocol	Section(s) changed
Version and date updated to:<br>Version 1.2<br>Date of amendment: 27 January 2020	Version 1.1<br>Date of amendment: 12 April 2019	Cover page
Updates to study synopsis as per updates to protocol		Study synopsis
Changed to:<br>*Up to 36 healthy subjects.*<br>*…additional participants will be enrolled to adjust for participants randomized to receive the higher dose vaccine by aerosol, but who do not complete the week 8 bronchoscopy.*<br><br>Changed to:<br>*36 subjects will be enrolled as follows:*<br>*Aerosol doses (n=22) or IM administration (n=14)*<br><br>Added:<br>*Additional participants will be enrolled into the second cohort to adjust for participants who do not complete the week 8 bronchoscopy, until at least 8 participants have received the higher dose of vaccine by aerosol and completed the 2 and 8 week bronchoscopies.*<br><br>Rationale: The sample size has been increased to 36 to allow for the recruitment of additional participants if needed; enrolment will continue into the higher dose aerosol group until at least 8 participants have received the higher dose of vaccine by aerosol and completed the week 2 and 8 bronchoscopies. This will ensure we have	28 healthy subjects.<br><br>28 subjects will be enrolled as follows:<br>Aerosol doses (n=18) or IM administration (n=10)	4.1<br>5.1<br>5.6

enough data points (at 2 and 8 weeks) to allow for meaningful comparisons between the lower and higher aerosol dose.		
Changed to:<br>*Bronchoscopy will be repeated at 2 and 8 weeks after immunization.*<br><br>Deleted:<br>*While is expected that two weeks is the time point when maximal immune responses are expected, the current data indicate delayed CD4+ T cell responses in some participants and CD8+ T cell responses lagged behind CD4+ T cell responses. Participants will be asked to have a repeat bronchoscopy at 12 weeks so we can assess the sustainability of vaccine-induced T cell responses in the airway following aerosol delivery and compare it with that following IM delivery.*<br><br>Added:<br>*Fig 3, Appendix 8*<br><br>Rationale:<br>The timing of the third bronchoscopy has been changed from 12 weeks to 8 weeks. Based on robust immunogenic T cell responses obtained at weeks 0, 2, and 8 following the low dose aerosol, we had postulated that a doubled dose would be stronger at inducing Ag-specific CD4 T cell responses at week 2 and thus, a sustained memory CD4 T cell responses in the airway (bronchoalveolar lavage -BAL) at week 12. However, the observations made from the first participants receiving the higher dose have indicated the opposite, showing that the high-dose aerosol did not induce a stronger Ag-specific T cell response; rather it appeared to have induced a lower response at week 2, leading to undetectable memory T cell responses at week 12 (see Fig 3, Appendix 8). Based on these initial findings from the high dose cohort, we have reasons to believe that the high dose may have caused T cell activation-associated cell death as a result of over-expressed Mtb antigen Ag85A.<br>The timepoint for the 3rd BAL will be changed from the current week 12 back to week 8, to be consistent with all of the timepoints in the low dose aerosol cohort. All other specifics will remain unchanged for aerosol and intramuscular groups and we will continue to collect safety bloods and blood for immunological testing at 12 weeks.<br>The same timepoints for bronchoscopies among the low and high dose aerosol, and intramuscular route will allow us to adequately compare the	*Bronchoscopy:* Bronchoscopy will be repeated at 2 and 12 weeks after immunization. While is expected that two weeks is the time point when maximal immune responses are expected, the current data indicate delayed CD4+ T cell responses in some participants and CD8+ T cell responses lagged behind CD4+ T cell responses. Participants will be asked to have a repeat bronchoscopy at 12 weeks so we can assess the sustainability of vaccine-induced T cell responses in the airway following aerosol delivery and compare it with that following IM delivery. …<br>*Examination of immune responses:* Mononuclear cells will be isolated from the bronchial lavage fluid at baseline, 2 and 12 weeks post-vaccination	Section 5.5<br>Appendix 2 Study Flow Chart<br>Appendix 8

safety and vaccine-induced immune responses across aerosol doses and routes of immunization. There is no change in dose; there have been no safety issues with the higher dose of 2x10^{6}cfu.		
Deleted<br>*Participants will be asked to consent for the Week 12 bronchoscopy and the genetic testing at their week 8 visit.*<br>Deleted:<br>*Subjects will be asked to consent separately for the week 12 bronchoscopy.*<br><br>Participants will be asked to consent for the baseline, 2 and 8 week bronchoscopies as well as the separate genetic consent at their screening visit. At each study visit, continuing consent for the study investigations is obtained and documented.	Participants will be asked to consent for the Week 12 bronchoscopy and the genetic testing at their week 8 visit.<br>Subjects will be asked to consent separately for the week 12 bronchoscopy.	Section 5.5

Amendment 1

The following table outlines the changes made to the protocol and the affected sections.

Change and Rationale for change	Original protocol	Section(s) changed
Version and date updated	Version 1.0<br>22 August 2014	Cover page
Telephone extn of Dr Smaill updated	Extn 76332	Extn 74190 (pages 2,4,5)
Addition of name and contact details of research coordinator		Page 5
Updates to study synopsis as per updates to protocol		Study synopsis
Updated Background, Rationale for Study, Experience with Adenovirus Vaccines to Date, and Respiratory Delivery of Vaccines based on updated references and new research findings. Results of our study using the humanized mouse model have been included and further support the rationale for aerosolized route of delivery of vaccine. The rationale for the study has been expanded to include details of the immune suppression in the lung that occurs with M.tb infection based on new research findings. Data to support that Ad-based vaccine induces both innate immune memory and T cell immunity in		Sections 1.1, 1.2, 1.3, and 1.4

Change and Rationale for change	Original protocol	Section(s) changed
the lung provided. The results of our initial phase I study of the IM administration of Ad5Ag85A have been updated to include that T-cells capable of recognizing multiple Ag85A epitopes were generated. Data is included confirming that in the non-human primate model Ad5Ag85A by aerosol improved survival rates.		
The section on induced sputum (IS) has been removed. From the data obtained from the first cohort of participants, we were not able to demonstrate that induced sputum was an adequate specimen to measure the immune response after aerosol administration of vaccine. Some participants were unable to produce enough IS even for minimal immune analysis and the quantity and quality of IS samples and viability of the cells obtained varied widely between visits of the same participant.<br>See appendix 8 for preliminary results.	1.4.2 Induced sputum<br>Induced sputum is a non-invasive method to collect cells from the respiratory tract, has been well characterized by our research group and is the preferred method for measuring inflammatory responses in allergic asthma subjects. In this study we will compare samples collected by induced sputum and bronchoalveolar lavage to better characterize the dynamics of the immune response in the respiratory tract after aerosol administration of vaccine. If we demonstrate induced sputum is an adequate specimen to measure the immune response after aerosol administration of vaccine, this non-invasive method of collecting samples can be incorporated into new studies to evaluate the respiratory route of vaccination.	Deletion of Section 1.4.2
The primary objective has been changed from the comparison of escalating doses of Ad5Ag85A (10 and 100 times the initial dose) to the comparison of one of two aerosol doses of Ad5Ag85A. The second dosing cohort of 10^7 pfu has been deleted and the third dosing cohort of 10^8 pfu has been changed to $2x10^6$ pfu.<br>Based on results from the lowest dose cohort, we are confident of the safety of the vaccine and have demonstrated very robust immune responses at the lowest dose of 10^6 pfu (see Appendix 8). We are now of the opinion that escalating the dose to 10^8 pfu may cause undesired adverse events and immune cell exhaustion. Doubling the dose to $2x10^6$ pfu will allow us to further investigate vaccine tolerability, safety and immunogenicity. Deleting the second dosing cohort will save time and resources. The intramuscular dose 10^8 pfu remains unchanged from our previous study. The objective of this current study is to provide data on the aerosol route of delivery and endorse the aerosol route over the IM route (i.e. a much smaller dose is not only safe but induces robustly desired immune memory at the site of TB exposure in the lung).	2.1 Primary Objectives<br>To evaluate the safety of a single administration of escalating doses of a recombinant replication-deficient human adenoviral (Ad5) TB vaccine (Ad5Ag85A) delivered to the respiratory tract by aerosol in healthy human subjects with a history of BCG vaccination.<br>2.2 Secondary Objectives<br>1. To compare the safety and immunogenicity of inhaled Ad5Ag85A administered by aerosol at the maximum tolerated dose with a single dose of 10^8 pfu administered by intramuscular injection in healthy subjects.<br>2. To evaluate the effect of pre-existing anti-adenoviral antibodies on the safety and immunogenicity of Ad5Ag85A.	Section 2.1

Change and Rationale for change	Original protocol	Section(s) changed
The section on induced sputum has been removed (see above)	To compare the antigen-specific immune responses obtained by induced sputum and by bronchoalveolar lavage.	Section 2.2
Delete Gamma interferon release assay (QFT) at 16 weeks. The development of a positive QFT is used in vaccine clinical trials as a surrogate for new TB infection. We are confident that vaccination with Ad5Ag85A is NOT associated with the development of a positive QFT. There were no conversions seen in the original study with the IM administration of Ad5Ag85A and none to date in the 6 participants in this study who have completed the week 16 visit. Given that the QFT assay measures immune responses to different antigens than are in the vaccine it is not anticipated that there would be conversion of the QFT test.	To evaluate whether vaccination with Ad5Ag85A is associated with the development of a positive gamma interferon release assay for TB.	Section 2.2
See above rationale for changing dosing cohorts.	3. Study description<br>This is an open-labeled phase 1 dose escalating single institution trial comparing a single dose of recombinant genetic TB vaccine Ad5Ag85A administered by aerosol with intramuscular injection of vaccine in healthy subjects with a history of BCG vaccination.	Section 3
The total number of participants has been reduced to 28.<br>The total number of aerosol doses has been changed from 26 to 18.<br>The original second aerosol cohort dose of 10^7 has been deleted and the aerosol dosing for the third cohort changed from 10^8 to $2x10^6$ pfu.<br>The cohorts have been renamed: the first cohort remains unchanged, the new second cohort compares the aerosol dose of $2x10^6$ with the original IM dose of 10^8 pfu.<br>The description of the SMC's review after the first two participants have received the lower dose by aerosol and their review of all AE's and SAE's for 4 weeks post vaccine after completion of the lower dose cohort, has been reworded to reflect the change in study design.<br>The justification for the dosing regimen, route of administration and study design has been updated.<br>References to higher doses have been deleted.<br>The rationale for the selection of the dose of $2x10^6$ for the second cohort, based on the preliminary data demonstrating excellent safety and robust immunogenicity, is provided.	36 subjects will be enrolled as follows: Aerosol dose escalation (n=26) or IM administration (n=10)<br>The first cohort of 8 BCG positive subjects will receive 10^6 pfu Ad5Ag85 vaccine administered using the Aeroneb® Solo Vibrating Mesh Nebulizer. Assuming acceptable safety outcomes, the second cohort of 8 BCG positive subjects will receive 10^7 pfu by aerosol. Assuming acceptable safety outcomes, the third cohort will consist of 20 subjects randomly allocated to receive 10^8 pfu by aerosol (n=10) or by single IM administration (n=10).<br>With each aerosol dose, we will first vaccinate 2 subjects. Assuming no grade 3 or 4 adverse events or any serious adverse events at least possibly associated with vaccine in subjects who have been followed for 2 weeks after administration of vaccine, we will proceed to immunize the rest of the subjects in the dose group. Reports detailing all AEs and SAEs for 4 weeks post-dose will be reviewed by the Safety Monitoring Committee (SMC) following each dose cohort. We will not move to the next dose group until the conditions are met as described in 6.4.1.	Section 5.1

Change and Rationale for change	Original protocol	Section(s) changed
	For the third cohort, a randomization list will be generated containing sequential codes linked to route of study vaccine assignment, either IM injection or aerosol. The codes will be assigned to subjects in the order in which they are enrolled. Once the subject has completed the screening examination and is eligible for vaccination, the research co-ordinator will access a password protected computer site for group allocation.<br>If the SMC recommends discontinuation of further aerosol vaccinations or no escalation to the next dose of aerosol vaccine, enrolled subjects who have received aerosol vaccine will continue to be followed, according to schedule. If recommended by the SMC, 10 additional subjects will be enrolled in the IM group and administered vaccine at a dose of 10^8 pfu. Subjects will be followed for a total of 24 weeks after vaccine administration.<br>5.1.1 Justification for dose escalation, route of administration and study design.<br>Because of the limited data from animal models and the difficulty extrapolating the pre-clinical results to human respiratory vaccine delivery due to differences in anatomy, respiratory immunologic tissues and responses to infecting organisms in humans and animals, a cautious dose escalating approach in human subjects will be employed in this study.<br>The maximum dose has been selected based on safety and immunogenicity data from our phase 1 study of intramuscular administration of Ad5Ag85A and data from other studies of Ad5 vaccines. In our completed phase 1 study of Ad5Ag85A, 10^8 pfu administered i.m was shown to be safe and well-tolerated in BCG+ humans. There were no vaccine-related serious adverse events. The most common adverse effect was mild to moderate local reaction at the injection site and mild systemic reactions e.g. headache and malaise, which resolved in 24 hours. This dose was robustly immunogenic in BCG+ healthy volunteers. The minimum dose chosen is two logs lower than the dose used in our Phase 1 study and a log lower than the dose of MVAAg85A administered to BCG vaccinated subjects using an Omron nebulizer in the recently completed aerosol study.<br>Data from other studies have shown that with doses of 1 x 10^{10} – 10^{11} viral particles, side effects such as fever and a transient drop in the	

Change and Rationale for change	Original protocol	Section(s) changed
	white cell count have been reported. A dose of 10^8 pfu administered i.m has been used safely in the study of Ad5rHer-2 in breast cancer and the Ad5 HIV vaccine trials have safely evaluated 10^9, 10^{10} and 10^{11} viral particles (within the range of 1 to $5x10^7$ and 10^9 pfu) A study of an experimental Ebola virus vaccine used doses ranging from $2x10^9$ to $2x10^{11}$ virus particles. Doses of 10^8, 10^9 and 10^{10} viral particles administered intranasally were safely used in a study of an adenovirus-vectored (AdHu5) intranasal pandemic influenza vaccine.<br>A dose of 1 or $2x10^9$ pfu of our Ad5Ag85A vaccine was administered intramuscularly, intradermally and endobronchially to cattle previously immunized with BCG and was found to be safe, immunogenic and immune protective.<br>In our recently completed Phase 1 IM study, immunological responses were significantly higher in the BCG positive group, with no evidence of increased toxicity. In this new study we will compare the safety and immunogenicity of vaccine at the highest dose by route of administration (IM or aerosol) in BCG positive individuals. For the third cohort (10^8 pfu), subjects will be randomly allocated to IM injection or the aerosol route to avoid selection bias.	
The exclusion criteria for cigarette smokers have been expanded to include current e-cigarette smoking, given the accumulating data that e-cigarettes may potentially be associated with lung damage	Current cigarette smokers and ex-smokers who have quit less than a year ago, as reported by the subject.	Section 4.2
Given that there is evidence of impairment of pulmonary immune in chronic marijuana users as well as a lack of good evidence of what may be a "safe" level of use, we will retain marijuana use in the last year as an exclusion, and add that subjects may be enrolled as long as they do not smoke marijuana for the duration of the study.	Subjects who give a history of last smoking marijuana more than a year ago may be enrolled	Section 4.2
Administrative clarification. Participants who are found ineligible because of initial unavailability for study visits and investigations may be rescreened once.	New	Section 4.2.2
The commercial interferon gamma release assay for TB has been changed by the manufacturer from Quantiferon® TB test to Quantiferon® TB GoldPlus test. This may slightly increase the sensitivity to detect latent TB in participants who are being screened for eligibility.	Quantiferon® TB test	

Change and Rationale for change	Original protocol	Section(s) changed
The screening period may be increased to 56 days. For women participants who agree to start hormonal contraceptives to meet the eligibility criteria for inclusion in the study, the screening period from will be up to 56 days, giving these women time to have completed one full menstrual cycle with hormonal contraception. Given that we are enrolling healthy participants, their health status and screening investigations to determine eligibility are unlikely to have changed over this period of time but ongoing eligibility is assessed at each study visit. Scheduling of participants for their Baseline visit may also necessitate a slightly longer screening period, given administrative and participant considerations, e.g. vacation, weather delays, laboratory availability.	28 days (4 weeks)	Section 5.2
As induced sputum will no longer be collected, all references to immunological testing on induced sputum have been deleted.<br>Appendix 7 has been deleted and the subsequent Appendices renumbered.<br>The bronchoscopy procedure has been described in greater detail: a total of 160 ml of sterile saline are instilled in 4x40ml separate aliquots and aspirated back, providing a total volume of 80–100 ml.	*Induced sputum:* Subjects will have an induced sputum sample collected at least one day before BAL. Briefly, subjects will inhale a hypertonic saline solution to stimulate coughing and the sputum expectorated will be processed to remove mucus and debris and the cells recovered used for antigen stimulation and measurement of immune responses (See Appendix 7).	Section 5.3
The additional optional bronchoscopy has been changed from week 8 post vaccination to week 12 and a week 12 visit added. Including the optional 12 week BAL will allow us to more reliably examine the kinetic changes of the immune responses. In our first cohort, we performed BAL at baseline, week 2 and week 8. While week 2 is usually the peak time of vaccine-induced T cell responses, a sustained response between weeks 2 and 8 was seen in some participants, and in all other participants, a reduced but readily detectable response was still seen at week 8. In order to better understand the memory T cell responses in the lung following aerosol vaccination, we propose to perform the optional BAL at 12 weeks. Given our understanding of the dynamics of memory T cell responses we are confident that, with the aerosol vaccine dose doubled, performing the third BAL at 12 weeks will be adequate to provide us with information on the persistence of memory T cell responses.<br><br>All participants to date have consented to the additional optional week 8 bronchoscopy and	*Bronchoscopy:* Bronchoscopy will be repeated at 2 and 8 weeks after immunization. It is expected that two weeks is the time point when maximal immune responses are expected but participants will be asked to have a repeat bronchoscopy at 8 weeks so we can assess the sustainability of vaccine-induced T cell responses in the airway following aerosol delivery and compare it with that following IM delivery. An interval of two to six weeks between bronchoscopies is the safe interval at which we would have our volunteers undergo the repeat procedure. Subjects will be asked to consent separately for the week 8 bronchoscopy.	Section 5.5

Change and Rationale for change	Original protocol	Section(s) changed
there have been no safety concerns with the additional bronchoscopy. Participants will be asked to consent for the week 12 bronchoscopy at the week 8 visit.<br>Elsewhere in this section, testing on BAL samples obtained at the week 12 bronchoscopy rather than at week 8 have been updated.<br>The immunological investigations, spirometry and safety bloods previously drawn at week 16 will be performed at the week 12 visit.<br>The week 16 visit is changed to a safety monitoring visit only; no further investigations are performed at that visit unless clinically indicated.		
Based on our preliminary research findings of evidence of alveolar macrophage training by Ad5Ag85A aerosol vaccination, we plan to examine gene expression with mononuclear cells from the bronchial lavage, at baseline and 12 weeks post vaccination in a subset of participants who have consented separately for this testing. No additional samples will be collected. Participants will be asked to consent for the genetic testing at the week 8 visit.	New	Section 5.5
The section on vaccine dosing and administration has been updated from escalating aerosol doses of 10^6, 10^7 and 10^8 to two doses of $1x10^6$ and $2x10^6$. Rationale provided above.	Doses ranging from 10^6 plaque forming units to 10^8 of Ad5Ag85A will be evaluated.	Section 6.1
Clarification provided for safety monitoring of the second cohort. We will not proceed to complete enrolment of this cohort until the first two subjects who received vaccine by aerosol have completed 2 weeks follow-up without dose-limiting toxicities.	We have previously demonstrated that the IM administration of 10^8 pfu of Ad5Ag85A in BCG-negative and positive subjects is safe. We predict that aerosol-delivered Ad5Ag85A will be well-tolerated by BCG vaccinated healthy human volunteers, but we will monitor closely for any adverse events and escalate the dose carefully. With each aerosol dose, we will first vaccinate 2 subjects. Assuming no dose-limiting toxicities in subjects who have been followed for 2 weeks after administration of vaccine we will proceed to immunize the rest of the subjects in the dose group.<br>There are no known risks associated with induced sputum collection, although light-headedness from coughing may occur.	Section 6.2
Appendix 2, the study flow chart has been updated to reflect the changes made to the protocol		Appendix 2
Appendix 8 has been added describing preliminary safety and immunogenicity findings	New	

STUDY SYNOPSIS

Study Title	**Phase I clinical trial to evaluate the safety and immunogenicity of Ad5Ag85A administered by aerosol**
Objectives	**Primary Objectives:**<br>To evaluate the safety and immunogenicity of a single administration of one of two doses of a recombinant replication-deficient human type 5 adenovirus-based (Ad5) TB vaccine (Ad5Ag85A) delivered to the respiratory tract by aerosol in healthy human subjects with a history of BCG vaccination<br>**Secondary Objectives:**<br>1. To compare the safety and immunogenicity of inhaled Ad5Ag85A administered by aerosol with a single dose of 10^8 pfu administered by intramuscular injection in healthy subjects with a history of BCG vaccination.<br>2. To evaluate the effect of pre-existing anti-Ad5 antibodies on the safety and immunogenicity of Ad5Ag85Avaccine.
Study Description	This is an open-labeled phase 1 single institution trial investigating one of two doses of a recombinant genetic TB vaccine Ad5Ag85A in healthy subjects with a history of BCG vaccination administered by aerosol using the Aeroneb® Solo Vibrating Mesh Nebulizer and by IM injection
Study Population	***Inclusion Criteria***<br>Subjects who meet all of the following criteria are eligible for enrolment:<br>1. Healthy human subjects who are between 18 and 55 years of age with a history of BCG vaccination.<br>2. HIV antibody negative<br>3. Able to understand and comply with protocol requirements and instructions; able to attend scheduled study visits and complete required investigations.<br>4. For women, negative pregnancy test and practicing two acceptable forms of contraception for the duration of the study (barrier contraceptive, birth control pill, surgically sterile, post-menopausal 2 years, abstinence)<br>5. For men, using barrier contraception for the duration of the study<br>***Exclusion Criteria***<br>Subjects who meet any of the following criteria are not eligible for admission to the study:<br>1. Pregnant or lactating women<br>2. Subjects who have any acute or chronic illnesses including active tuberculosis, any relevant findings on physical examination or are receiving any drug treatment in the opinion of the investigator likely to affect the immune system including current use of inhaled or nasal steroids.<br>3. Subjects with a history of any bleeding disorder or receiving any drug treatment that in the opinion of the investigator may increase the risk of bleeding<br>4. Subjects with a history of respiratory disease, e.g. asthma, chronic bronchitis, COPD<br>5. Current smokers, current e-cigarette smokers and ex-smokers who have quit within the last year, as reported by the subject<br>6. Subjects with clinically significant abnormality of baseline spirometry tests<br>7. Any health-related condition for which study bronchoscopy is contraindicated<br>8. Subjects who have a history of active or latent TB infection or whose PBMCs are responsive to ESAT6/CFP10 stimulation using a commercial interferon gamma release assay for TB [consistent with latent TB infection].

	9. Subjects whose baseline laboratory values are outside of the normal range unless the abnormality is considered not to be of clinical relevance by the Investigator. A single repeat test is allowed during the screening period.<br>10. Subjects whose use of alcohol or drugs would, in the opinion of the investigator, interfere with adherence to the study protocol.<br>11. Subjects who are using, or have a history of using, inhaled cocaine, metamphetamine or other inhaled or smoked recreational drugs. Subjects who give a history of last smoking marijuana more than a year ago may be enrolled, as long as they do not smoke marijuana for the duration of the study.<br>12. Failure to provide written consent.<br>13. Known allergy to vaccine components<br>14. Previous vaccination with Ad5Ag85A or any other experimental TB vaccine<br>15. Known exposure to active TB within past 6 months or subjects whose occupation puts them at increased risk of TB exposure (based on Hamilton Health Science/St Joseph Healthcare list of high risk personnel)<br>16. Any abnormality on chest x-ray suggestive of active or remote tuberculosis infection or evidence on chest-x-ray of clinically significant respiratory disease.<br>17. PPD skin test within last 12 months<br><br>Indications for a delay in vaccination/enrolment:<br>1. Subjects with symptoms of an upper respiratory tract infection within 4 weeks<br>2. Subjects who have received any blood product or immunoglobulin infusion within the past 3 months<br>3. Receipt of any vaccine within past 30 days or planned vaccination within 21 days after receiving the experimental TB vaccine. Intranasal administration of FluMist® is contraindicated for the duration of the study
Study Procedures	Ad5Ag85 Safety and Immunogenicity Study; Up to 36 subjects with a history of BCG vaccination will be enrolled<br><br>Participants will undergo screening investigations, including clinical history, physical examination, blood tests, chest x-ray and pulmonary function tests to determine eligibility. At baseline, and at intervals following administration of vaccine, participants will be followed for adverse events and undergo BAL, induced sputum and blood examination for the assessment of immunological responses.<br><br>**Aerosol dose escalation (n=25) or IM administration (n=14) (assuming acceptable safety outcomes)**<br>1. 8 plus 3 subjects 10^6 pfu by aerosol<br>2. Up to 28 subjects randomized to receive $2x10^6$ pfu by aerosol (n=14) or 10^8 pfu by IM administration (n=14). The actual number in each group will depend on how many participants complete all the planned study visits.
Study Endpoints	***Primary Safety Endpoint:***<br>Signs and symptoms; laboratory toxicity<br><br>***Secondary Endpoint:***<br>Immunogenicity will be compared among the groups by determining the level and quality of antigen-specific T cells and antibodies by cytokine and antibody ELISA, Elispot assay and intracellular cytokine staining (ICS) in blood, induced sputum and bronchoalveolar lavage fluid

LIST OF ABBREVIATIONS

Ad	Adenovirus
Ad5	Human type 5 adenovirus
Ad5Ag85A	Replication deficient adenoviral vector expressing Ag85A
AE	Adverse Event
BAL	Bronchoalveolar lavage
BCG	Bacille Calmette Guerin
BP	Blood Pressure
CIOMS	Council for International Organizations of Medical Sciences
COPD	Chronic obstructive pulmonary disease
CFR	Code of Federal Regulations
CMV	Cytomegalovirus
CRF	Case Report Form
DLCO	Diffusing capacity of the Lung for Carbon Monoxide
DNA	Deoxyribonucleic Acid
FEV_1	Forced expiratory volume in 1 second
FVC	Forced Vital Capacity
GCP	Good Clinical Practice
GMP	Good Manufacturing Practice
HIV	Human immunodeficiency virus
HPFB	Health Products and Food Branch
ICF	Informed Consent Form
ICH	International Conference on Harmonization
IEC	Independent Ethics Committee
IM	intramuscular
IRB	Institutional Review Board
ITT	Intent to Treat
kg	Kilogram
MedDRA	Medical Dictionary for Regulatory Activities
mg	Milligram
M. tuberculosis	Mycobacterium tuberculosis
MVAAg85A	Modified vaccinia virus Ankara expressing M. tb antigen Ag85A
OTC	Over the Counter
PBMCs	Peripheral Blood Mononuclear Cells
Pfu	Plaque forming units
PPD	Purified protein derivative
RCA	Replication Competent Adenovirus
REB	Research Ethics Board
RNA	Ribonucleic acid
SAE	Serious Adverse Event
SD	Standard Deviation
SMC	Safety Monitoring Committee
TB	Tuberculosis
TEAE	Treatment Emergent Adverse Event
TPD	Therapeutic Products Directorate
TU	Tuberculin unit
U.S./USA	United States of America
VRC	Vaccine Research Center
WHO	World Health Organization

TABLE OF CONTENTS

PROTOCOL/AMENDMENT APPROVAL PAGE(S) **2**
IMPORTANT CONTACTS AT MCMASTER UNIVERSITY MEDICAL CENTRE. **5**
HISTORY OF AMENDMENTS TO PROTOCOL **6**
STUDY SYNOPSIS **16**
Inclusion Criteria *16*
Exclusion Criteria *16*
LIST OF ABBREVIATIONS **18**
1. **INTRODUCTION** **22**
1.1 BACKGROUND 22
1.2 RATIONALE FOR THE STUDY 23
1.2.1 New TB vaccine candidates and immunization regimens *24*
1.3 EXPERIENCE WITH ADENOVIRUS VACCINES TO DATE 27
1.3.1 Human trials evaluating recombinant adenoviral HIV vaccines *27*
1.3.2 Other human trials using recombinant adenovirus vectors *27*
1.3.3. Our preclinical studies using recombinant replication-deficient adenoviral TB vaccine in rodents, guinea pigs, goats and cattle *28*
1.3.4 Our clinical experience in using recombinant replication-deficient adenoviral TB vaccine in healthy human volunteers *29*
1.3.5 Our clinical experience in using recombinant replication-deficient adenoviral vaccine in cancer patients *30*
1.3.6. Potential side effects associated with the use of adenoviral vector for the purpose of vaccination *30*
1.3.7 Consideration of pre-existing anti-Ad5 immunity that may limit the use of Ad5 vectors *31*
1.4 RESPIRATORY DELIVERY OF VACCINES. 32
1.4.1 Choice of nebulizer for the aerosol delivery of vaccine and evaluation of Aeroneb® Solo for delivering vaccine *32*
2 **OBJECTIVES** **33**
2.1 PRIMARY OBJECTIVE 33
2.2 SECONDARY OBJECTIVES 33
3. **STUDY DESCRIPTION** **33**
3.1 STUDY SITE 34
4. **STUDY POPULATION** **34**
4.1 NUMBER OF PATIENTS 34
4.2 SELECTION CRITERIA 34
Inclusion Criteria *34*
Exclusion Criteria *34*
4.2.1 Indications for a delay in enrolment/vaccination *35*
5. **STUDY PROCEDURES** **36**
5.1 STUDY OUTLINE 36
5.1.1 Justification for dosing regimen, route of administration and study design *37*
5.2 SUBJECT SELECTION 37
5.3 IMMUNOLOGIC EVALUATIONS PRIOR TO VACCINATION 38
5.4 VACCINE ADMINISTRATION 39

5.5 FOLLOW UP PROCEDURES 39
5.6 SUBJECT DISCONTINUATION 40

6. INVESTIGATIONAL PRODUCT 41
6.1 VACCINE DOSING AND ADMINISTRATION 41
6.1.1 Packaging and labeling 41
6.1.2 Storage, Dispensing and Reconciliation of Study Drug 42
6.1.3 Dose modification 42
6.2 SAFETY AND PRECAUTIONS 42
6.3 SAFETY MONTORING COMMITTEE 43
6.4 SAFETY MONITORING PLAN 43
6.4.1 Dose limiting toxicities 44
6.5 ADDITIONAL BACKGROUND INFORMATION OF ADENOVIRAL TB VACCINE 44
6.5.1 Name and Chemical Information 44
6.5.2 Chemical Structure 44
6.5.3 Mechanisms of Action 44
6.5.4 Experimental anti-TB Activity 44
6.5.5 Toxicology/Safety Issues 44
6.5.6 Pharmacokinetic Studies 46

7. ADVERSE EVENTS AND SERIOUS ADVERSE EVENTS 46
7.1 ADVERSE EVENT (AE) DEFINITION 46
7.2 ADVERSE EVENT REPORTING PERIOD 46
7.3 ADVERSE EVENT DOCUMENTATION 47
7.4 ADVERSE REACTION TO THERAPY DEFINITION 47
7.5 SERIOUS ADVERSE EVENT (SAE) DEFINITION 48
7.6 REPORTING OF SERIOUS ADVERSE EVENTS 48
7.7 SERIOUS ADVERSE EVENT FOLLOW-UP 49
7.8 SERIOUS ADVERSE EVENT DOCUMENTATION 49
7.9 EXPECTED ADVERSE EVENTS 49

8. DATA MANAGEMENT AND STATISTICS 50
8.1 MONITORING 50

9. ETHICAL, REGULATORY AND ADMINISTRATIVE ISSUES 51
9.1 RESEARCH ETHICS BOARD 51
9.2 INFORMED CONSENT 51
9.3 CASE REPORT FORMS (CRFS) 52
9.4 ESTIMATED DURATION OF THE STUDY 53
9.5 RETENTION OF SUBJECT RECORDS AND STUDY FILES 53
9.6 CONFIDENTIALITY 53
9.7 NOTIFICATION OF PRIMARY CARE PHYSICIAN 53
9.8 DISCLOSURE AND PUBLICATIONS 53
9.9 PROTOCOL AMENDMENTS 53

INVESTIGATOR AGREEMENT 54
10. REFERENCES 55
11. APPENDICES 63
APPENDIX 1 - CLINICAL LABORATORY VALUES 64
APPENDIX 2 - STUDY FLOWCHART 65
APPENDIX 3 - LIST OF IMMUNOSUPPRESSIVE MEDICATIONS 66
APPENDIX 4 - DECLARATION OF HELSINKI 67

APPENDIX 5 - CRITERIA FOR TOXICITY AND ADVERSE EVENT REPORTING........................... 68
APPENDIX 6 - BRONCHOSCOPY STANDARD OPERATING PROCEDURE 69
APPENDIX 7 - *IN VITRO* EVALUATION OF THE AERONEB® SOLO MICROPUMP AEROSOL DELIVERY SYSTEM FOR DELIVERING AD5AG85A TB VACCINE TO HUMAN SUBJECTS ...... **ERROR! BOOKMARK NOT DEFINED.**
APPENDIX 8 - PRELIMINARY SAFETY, IMMUNOGENICITY AND RESULTS OF INDUCED SPUTUM COLLECTION ....**ERROR! BOOKMARK NOT DEFINED.**

1. INTRODUCTION

1.1 BACKGROUND

TB in the world. TB has haunted mankind for thousands of years and yet still remains one of the leading causes of death by a single infectious agent. In 2016, there were an estimated 10.4 million new cases of TB and 1.8 million people died from TB [1]. One third of the world's population has been infected by *Mycobacterium tuberculosis* and have latent TB and 5-10% will develop active TB disease sometime in their lives, unless co-infected with HIV [1, 2].

TB and HIV co-infection in the world. HIV-infected persons are especially susceptible to TB, and are 21 to 34 times more likely to develop active TB disease [3]. At least one third of the estimated 34 million people living with HIV infection are infected with *M.tuberculosis* and in 2012 about 320,000 people died of HIV-associated TB [1]. In 2012 there were an estimated 1.1 million new cases of HIV-positive TB cases, 75% of which were living in Africa and almost 25% of deaths among people with HIV are due to TB.

TB in North America. Although the incidence of TB in the US and Canada is much lower than in developing countries, TB is still an important health problem. There are an estimated 11 million Americans with latent *M. tuberculosis* infection and a total of 9,105 TB cases (a rate of 2.8 cases per 100,000 persons) were reported in the United States in 2017 [4]. Overall the percentage of persons with TB who were HIV-positive is 6%, increasing to 10% for persons aged 25-44.

In Canada in 2016, there were 1,737 cases of active TB reported, for an incidence rate of 4.8 per 100,000, but there were significant differences reported by region [5]. In particular there were a disproportionately high number of cases reported in the Inuit population where the incidence rate was 35 times the national average at 170.1 per 100,000 and nearly 300 times the rate of Canadian born non-aboriginal people. In 2016, 70% of all reported TB cases were among foreign-born individuals, 19% among Canadian-born Aboriginal people and 10% of cases were among Canadian-born non-Aboriginal people. In Manitoba, Saskatchewan and the North (which includes Northwest Territories, Nunavut and Yukon), the majority of cases were among Canadian-born Aboriginal peoples.

In Canada, the incidence of latent TB infection is higher in high risk or disadvantaged populations (HIV positive persons, immigrants from TB and HIV endemic countries, the homeless, drug users, aboriginal peoples, health care workers and the elderly) [5]. A study by Menzies et al in 1998 found that up to 38% of health care workers in four Canadian cities were TB skin test-positive [6]. Brassard reported in 2006 that 21% of new immigrant children in select elementary and secondary schools in Montreal were positive [7].

TB outbreaks still occur from time to time in Canada, particularly in northern aboriginal communities and homeless shelters. Anti-TB therapeutic regimens are lengthy (6 months to a year) and entail the use of at least three drugs, and current treatment recommendations for latent TB are isoniazid for 6-12 months [8]. Drug access, cost and poor adherence are among the

reasons for increasing drug-resistant TB cases. 8.9% of all TB isolates tested in 2016 in Canada were resistant to at least one of the first-line TB drugs; 17 cases were a multi-drug resistant strain [9]. Although there has been little change in the overall pattern of TB drug resistance in Canada between 2006 and 2016, there is growing worldwide concern regarding resistance and the emergence of extensively drug-resistant TB (XDR-TB) [1, 9].

Vaccination against TB. Currently, the only TB vaccine in use is BCG (Bacille Calmette Guerin), an attenuated strain of *Mycobacterium bovis,* developed more than 90 years ago and administered globally for over 50 years [10]. However, the global epidemic of TB attests to the ineffectiveness of this vaccine. BCG prevents only 5% of all deaths from TB and although it is efficacious in children and protects against tuberculous meningitis and disseminated disease, any immune protection conferred by BCG lasts for only 10-15 years and importantly, it fails to prevent pulmonary TB infection in adults [11]. Several immune-stimulatory *M. tuberculosis* proteins (antigens) are not expressed by BCG and the efficacy of vaccine may be affected by the diverse genetic background of human populations. Both the USA and Canada are among a number of developed countries where BCG is not part of a national immunization program, due to its uncertain effectiveness and the overall low prevalence of infection in the general Canadian born population, but BCG is still offered to all newborn infants in Nunavut.

Tests for latent TB. There are two types of test available to detect latent TB infection: the (purified protein derivative) PPD skin test and interferon gamma release assays (e.g. Quantiferon® - TB test) [8]. Testing and treatment of latent infection is recommended for persons at high risk for developing tuberculosis and persons whose activities place them at increased risk of exposure, such as health care workers. PPD itself can re-stimulate remote hypersensitivity. Most persons with >10mm induration to 10 tuberculin units (0.1ml) intradermal injection are infected with *M. tuberculosis*; lesser induration may be due to BCG vaccination, cross-reaction with other mycobacterial species or reflect latent TB in HIV positive individuals.

1.2 RATIONALE FOR THE STUDY

While there has been progress in the fight against tuberculosis, particularly with an improvement in the mortality rate, the global burden of pulmonary TB remains enormous.

Immunization represents the most cost-effective medical means to prevent and treat disease, and it is believed that even a mildly improved TB vaccination strategy over the current BCG vaccine will have an enormous socio-economic impact. Thus, in order to control and eventually eliminate TB, better vaccination initiatives, particularly those amenable to the respiratory route of vaccination, are needed and immunization programs developed for populations at increased risk of TB. Recent research has identified the suppressed macrophage activation and delayed T cell immunity at the respiratory mucosal surface in response to M.tb infection to be a major mechanism for unsatisfactory protection by parenteral BCG vaccination. Respiratory mucosal route of vaccination is the most potent in correcting this problem. Of note, most of the fourteen TB vaccine candidates currently in the global pipeline are being administered via a parenteral route. There is an urgent need to clinically evaluate suitable vaccine candidates following respiratory mucosal administration.

The general objectives of our TB vaccine research program are:

- ♦ To develop a safe and effective vaccine for persons who are at increased risk of contracting tuberculosis or reactivating latent tuberculosis.
- ♦ To develop a safe and effective booster vaccine for persons who have been previously vaccinated with BCG and are at increased risk of contracting tuberculosis or reactivating latent tuberculosis, and
- ♦ To identify safe and effective vaccines for TB patients who are infected with multi-drug resistant M. tuberculosis.
- ♦ To develop a safe and effective vaccine for HIV/TB co-infected patients
- ♦ To develop a safe and effective respiratory-mucosally deliverable TB vaccine

1.2.1 New TB vaccine candidates and immunization regimens

The Stop TB Partnership Working Group on New TB vaccines provides on their website a summary of TB vaccines under development and lists those that are currently being tested in clinical trials and preclinical studies with a description of the specific vaccine types [12].

Mycobacterial organism-based. Strategies have been taken to improve the immunogenicity of the current BCG vaccine by adding an immune adjuvant, either incorporating a cytokine transgene into the conventional BCG organism or administering a recombinant cytokine such as IL-12 to enhance the immunogenicity of BCG vaccine. Mutant strains of BCG have been developed without certain genes required for the biosynthesis of essential amino acids, so they are unable to replicate but remain immunogenic. While these strains are safer for immune compromised hosts, an immune-adjuvant would be needed to improve immunogenicity.

Protein/peptide-based. These vaccines consist of defined soluble mycobacterial proteins or peptides and have been developed primarily for parenteral administration. Safety is a prominent feature of these vaccines. However, major limitations to such vaccines include: 1) requirement for an adjuvant formulation and the administration of multiple doses; 2) poor stimulation of CD8 T cells by these vaccines; and 3) expensive production costs. Subunit vaccines, notwithstanding these drawbacks, are potential vaccine candidates for immune competent and immunocompromised hosts, if their immunogenicity could be improved and cost reduced.

Recombinant bacterial plasmid DNA-based. Bacterial plasmid DNA (naked DNA) has been engineered to express the gene coding for a selected *M. tuberculosis* antigen. This type of vaccine is non-living, the DNA backbone, itself rich in unmethylated CpG motifs, is a type 1 immune adjuvant, repeated intra-muscular vaccination results in activation of both anti-TB CD4 and CD8 T cells, and they could also be used as a therapeutic vaccine. DNA-based TB vaccines, similar to protein-based, do not convert the skin PPD test. Compared to viral-based vectors, however, DNA vectors elicit lower levels of antigen expression due to low gene transfer efficiency and their clinical application is limited.

Recombinant vaccinia viral-based. Vaccinia vector has been used to express *M. tuberculosis* antigen and has demonstrated immune-stimulatory effects in animal models. However, this form

of TB vaccine is unlikely to be used as a mainstay platform for TB vaccination because of low levels of antigen expressed, since this expression can only be driven by poxviral regulatory elements and not by common eukaryotic promoters such as CMV promoter. However, vaccinia TB vaccines such as recombinant modified vaccinia virus Ankara expressing *M. tuberculosis* antigen Ag85A (MVAAg85A) may be used as a robust booster after priming vaccination with BCG or DNA vaccine.

A recombinant, replication-deficient modified vaccinia Ankara (MVA) expressing mycobacterial mycolyl transferase Ag85A ($5x10^7$ plaque forming units) given intradermally to healthy human volunteers with or without a history of BCG vaccination was safe and well-tolerated and effective in activating T cells [13]. MVA85A was also administered to M.tb and HIV-infected persons in South Africa and shown to be safe and immunogenic and induced polyfunctional CD4 cells expressing IFN-gamma, TNF-alpha and IL-2. Responses were, however, lower in HIV-infected vaccinees [14].

In a recently published phase 2b randomized placebo controlled efficacy trial, a single intradermal dose of MVAAg85A administered to infants who had previously received BCG vaccination failed to significantly improve the protection against tuberculosis, although vaccination was well tolerated and induced modest cell-mediated immune responses [15]. More infants who received MVA85A had a least one local adverse event (89% vs 45%) but the number of systemic adverse events or serious adverse events did not differ between the groups. Intradermal MVAAg85A boosting failed to significantly improve protection over that by BCG priming in these infants, highlighting the need for its evaluation as a respiratory mucosal boosting vaccine.

White et al have shown that MVA85A is safe and immunogenic when administered by aerosol to macaques previously vaccinated with BCG [16] and recently this group completed a randomized, placebo controlled safety and immunogenicity study comparing a single dose of 1×10^7 pfu MVA85A administered by the aerosol route with the intradermal route in 22 healthy BCG-vaccinated subjects [17]. Subjects were randomized to be vaccinated by either the inhaled aerosol route or the intradermal route and the systemic and mucosal immunogenicity of the vaccine evaluated by comparing T cell responses in blood and bronchoalveolar lavage samples. Vaccine administration by aerosol was shown to be safe and immunogenic with spirometry remaining normal [18].

Recombinant adenovirus-based. Adenoviruses (Ad) are double stranded DNA viruses responsible for self-limiting respiratory illness in humans [19]. Adenoviral-related illness is common in the general population and adenovirus accounts for 5-10% of mild upper respiratory infection in children and infants. Approximately 40-70% of humans have antibodies to Ad5. Vaccination trials were safely completed in the 1950's using oral ingestion of virus types 4 and 7. While there have been isolated reports of severe respiratory infection associated with adenovirus serotype 14 with small clustered outbreaks being reported [20, 21], overall despite rare cases of severe infection, the vast majority of adenoviral infections are benign and self-limiting.

Adenovirus can be readily manipulated to incorporate large segments of foreign DNA, including those for *M. tuberculosis* antigens [22]. As a virus, adenovirus has the ability to infect cells, direct transcription and subsequent translation of viral proteins, including the newly inserted genes. Ad5 has widely been explored for gene therapy both in animals and humans. Recombinant adenoviral vectors, administered via the intramuscular, intranasal or oral route, have been developed as vaccines for several infectious diseases including HIV, hepatitis B virus, influenza, measles, rabies, Ebola, Angola Marburg virus, and hantavirus, with demonstrated immunogenicity and efficacy in animal models [22-30]. Intranasal administration of DEF201, a replication deficient human adenovirus type 5 vector that encodes human consensus interferon alpha, to hamsters was shown to be well-tolerated and protective against challenge with phlebovirus infection [31].

With expertise in adenoviral technology/gene transfer we have pioneered the development of adenoviral TB vaccine candidates and evaluated these vaccines in various experimental models (see below). Adenoviral vector, when used as a vaccine, rather than a gene therapy vector, is associated with several features: 1] ample safety data are available from human immunization programs; the live enteric-coated adenoviruses of serotypes 4 and 7 have been given orally to the US military recruits with proven safety and effectiveness [32]. 2] it is genetically rendered replicate-deficient; 3] of all vector systems, it derives the strongest transgene expression and gives rise to continuously raised levels of transgene protein in vivo for 2 to 3 weeks; 4] it has a broad range of target cells in vivo, which allows vaccination via any desired route; adenoviral backbone is a type 1 immune adjuvant; 5] in contrast to gene replacement therapy for genetic diseases that requires permanent transgene expression, adenoviral-derived strong, relatively transient transgene expression suits well the purpose of effective vaccination where the continuous presence of antigen is not required; and 6] gene expression is still feasible despite anti-adenoviral antibodies present in many human subjects.

The heterologous "prime-boost" concept for TB vaccination. While it is of importance to develop better TB vaccines suitable for immunizing newborns, it is equally important to develop vaccines that could be used to boost anti-TB immune responses in adolescents and adults who were previously BCG-vaccinated. As aforementioned, BCG vaccine given shortly after birth fails to protect from adult pulmonary TB but it does protect from severe forms of childhood TB and more than 85% of global populations have been BCG-vaccinated. Based on its proven safety and widespread use as an infant vaccination, it is likely that BCG will continue to be used in human immunization programs as a primary (prime) vaccine. However there is a need to boost the immune response/protection, particularly in the lung, in BCG-vaccinated individuals, but it remains unclear what to use as a safe and effective boost vaccine. BCG vaccine itself is not appropriate because the immune response to the initial BCG exposure will restrict the efficacy of subsequent BCG vaccination and of importance, both clinical and experimental studies indicate that repeated BCG administrations fail to boost immune responses and protection triggered by previous BCG prime immunization [33-35]. Recent evidence suggests that a viral-based booster vaccine may successfully improve the immune responses in BCG vaccinees (heterologous prime-boost vaccination regimen) [36].

1.3 EXPERIENCE WITH ADENOVIRUS VACCINES TO DATE

There have been or are on-going several clinical trials evaluating the safety and immunogenicity of recombinant replication-deficient human adenoviral vaccines.

1.3.1 Human trials evaluating recombinant adenoviral HIV vaccines

Recent HIV vaccine clinical trials using Ad5 vectors expressing three HIV proteins were stopped early because of futility [37-39] and in addition a transient increase in HIV infection was observed in the men with high pre-existing Ad5 antibody titers in the Step study, a double-blind, randomized, placebo-controlled trial in 3000 healthy uninfected volunteers [37]. The hazard ratio (HR) of HIV infection between vaccine and placebo recipients was higher in Ad5 positive men (HR 2.3 [95% CI 1.2 – 4.3] and uncircumcised men (3.8 [1.5 – 9.3], but was not increased in Ad5 seronegative men (1.0 [0.5 – 1.9]) or circumcised men (1.0 [0.6-1.7]) [37]. However, a similar relationship was not observed in the Phambili trial where the same Ad5 HIV vaccine was tested in Africa although long-term follow-up has suggested an increase in HIV incidence in the vaccinated group [40]. No increased incidence of HIV was observed in HVTN505, a phase IIb study of an investigational prime boost strategy that used a DNA priming vaccine administered three times over 8 weeks and an Ad5 boost at 24 weeks [38]. It has been argued that Ad5-induced activation of CD4+ T cells, the specific target for HIV, may play a role in increased HIV infection, and if this were the case, it may also apply to any HIV vaccine or other Ad vectors [41]. Although Ad5-based vaccines for HIV are no longer appropriate, the effort in developing Ad5-based vaccines against other infections such as TB needs to continue.

1.3.2 Other human trials using recombinant adenovirus vectors

***Healthy subjects*.**
When a replication-deficient Ad5 gene transfer vector was injected intradermally, no adverse events were observed except local induration and local inflammatory infiltrates comprising lymphocytes, macrophages and NK cells [42]. When the same Ad vector was sprayed to the respiratory tract of healthy subjects, no major adverse events related to the vector were observed in any subjects [43]. Transient fever occurring in a few subjects resolved spontaneously within 24 hours and viral cultures of blood, urine, stool and throat and radiologic tests were all negative. In another study, when Ad5 vector was given intradermally or intrabronchially to healthy subjects, mildly increased circulating interleukin-6 levels were found only in the intradermal subjects [44]. A summary of the safety profile of Ad5 trials since 1993 confirmed there was no evidence of appearance of replication-competent Ad in any samples examined [45].

An Ad5 influenza vaccine administered to 24 healthy adult volunteers either by a skin patch or intranasally was safe and well tolerated and induced a rise in serum hemagglutination-inhibition antibodies. The nasal vaccine was more potent than the epicutaneous route [46].

Two repeated immunizations, administered 28 days apart, of 10^8, 10^9 and 10^{10} viral particles (vp) administered intranasally were carried out in a recently completed Phase 1 study of the safety and immunogenicity of an adenovirus-vectored (Ad5) intranasal pandemic influenza vaccine in

healthy volunteers [47]. It was found safe and well tolerated even with the highest dose of vp [personal communication].

Four of fifteen human volunteers immunized by IM injection of two DNA plasmids and boosted with Ad5-vectored malaria vaccines were protected against live malaria sporozoite challenge following mosquito feeding. The vaccine regimen was safe and well tolerated with mostly mild adverse events at the site of injection [48].

A replication defective recombinant Ad5 vaccine expressing Ebola virus envelope glycoprotein administered IM was immunogenic in a recent NIH dose-escalation phase 1 study and subjects developed antigen specific humoral and cellular immune responses [49]. The highest dose used (2×10^{10} viral particles) was well-tolerated.

The safety and immunogenicity of an oral adenovirus serotype 4 vector vaccine expressing the haemagglutinin from an avian influenza A H5N1 has recently been reported. Abdominal pain, diarrhea and nasal congestion was more significant in vaccinees, but no serious treatment related events occurred [50]. Only 13 of 123 (11%) vaccinees seroconverted, however after IM boosting with H5N1, 100% of vaccinees in the higher dose cohort achieved seroprotection compared with 18% in the placebo group.

Subjects with medical conditions

Recombinant replication-deficient adenoviral vectors have been widely used in clinical trials for cancer patients ([51] not discussed here in detail), Ad5 gene transfer vectors encoding vascular endothelial growth factor (VEGF), fibroblast growth factor or CFTR have been evaluated in patients with cardiac conditions, cystic fibrosis or peripheral arterial disease. Ad5 vector expressing VEGF (4×10^8 to 4×10^{10} particles were injected to the muscle of 18 patients with claudication [52]. Local edema and rash were the most common adverse event. In a phase II study, Ad5VEGF (4×10^{10} particles) was intramuscularly injected into the lower extremities of 105 patients with disabling intermittent claudication [53] and it was found well-tolerated. In a different study, Ad5 expressing VEGF (2×10^{10} pfu) was injected into the ischemic lower-limb of 18 patients with chronic lower limb ischemia via a catheter [54]. No major gene transfer-related side effects were observed. An Ad5 vector expressing FGF (10^{10} particles) was injected via the coronary to 52 patients with stable angina [55]. The vector was found well-tolerated and did not cause any permanent adverse effects. Gene therapy studies in cystic fibrosis patients have confirmed that Ad5 is safe when administered by aerosol [56]

1.3.3. Our preclinical studies using recombinant replication-deficient adenoviral TB vaccine in rodents, guinea pigs, goats and cattle

Based on our experience developing and using recombinant replication-deficient, human type 5 adenoviral gene transfer vectors [57-98], we have developed and evaluated a recombinant replication-deficient adenoviral vector expressing an M. tuberculosis immunogenic antigen Ag85A (Ad5Ag85A) in different animal models [35, 92, 94, 97, 99, 100] (Ref.[30, 98, 101-104] for related review/chapter articles). Ag85A is highly conserved among all mycobacterial species and is immune-dominant both in M. tuberculosis-infected humans and experimental animals.

Previous reports have indicated that when expressed in plasmid DNA vectors, this antigen confers immune protection from M. tuberculosis challenge in experimental models.

In murine models, a range of Ad5Ag85A doses (10^7 to 10^8 pfu) were evaluated following intramuscular injections and intranasal administration and were found safe, well-tolerated and associated with protection when challenged with M. tb in both BCG naïve and BCG-vaccinated or DNAAg85A-vaccinated animals [92, 94, 105, 106].

Ad5Ag85A vaccine was evaluated in a bovine model through our collaboration with the British Veterinary Laboratories Agency. Following intramuscular injection of 10^9 pfu of Ad5Ag85A, the vaccine was safe and well tolerated in both BCG-naïve and BCG-vaccinated calves [97]. Ad5Ag85A immunization in calves does not result in the conversion of PPD skin test [97]. Intramuscular Ad5Ag85A immunization was found to activate both CD4 and CD8 T cells in both murine and bovine models. Similar results and enhanced protection from virulent *M. bovis* were also observed in a separate study where BCG-vaccinated calves were injected intradermally with $2x10^9$ pfu of Ad5Ag85A [107]. Using a prime-boost vaccination strategy, Ad5Ag85A delivered either intradermally or via the endobronchial route in cattle was safe and induced similar systemic and local interferon-gamma responses, with a delay in the response in BAL compared to systemic responses [108].

Ad5Ag85A has also been evaluated in a guinea pig model through our collaboration with scientists at Texas A&M University. The guinea pigs primed with BCG and subsequently boosted intranasally with Ad5Ag85A significantly outlived those only vaccinated with BCG or those vaccinated with BCG and intramuscularly boosted with Ad5Ag85A after pulmonary *M. tuberculosis* challenge [109].

In another study via our collaboration with the Spanish Animal Health Research Institute, goats primed with BCG and boosted with Ad5Ag85A were challenged with *M. caprae*. Vaccinated animals had reduced lung pathology and significant reductions in bacterial load and there were no adverse effects reported in the animals [100].

Ad5Ag85A was also recently evaluated in our humanized mouse model engrafted with human immune cells and was found, when delivered via the respiratory tract, to provide much enhanced protection against Mtb than by intramuscular delivery [110].

1.3.4 Our clinical experience in using recombinant replication-deficient adenoviral TB vaccine in healthy human volunteers

The pre-clinical safety and immunogenicity investigations formed the basis for our recently published Phase I clinical trial [36]. We have successfully evaluated the safety and immunogenicity of a single intramuscular injection of 10^8 pfu Ad5Ag85A in 12 BCG-naïve and 12 previously BCG-immunized healthy adults. Vaccination was found to be safe and well tolerated in both trial volunteer groups, with most side effects related to grade 1 injection site reactions. Ad5Ag85A was immunogenic in both trial volunteer groups and stimulated T cells capable of recognizing multiple Ag85A epitopes [111], but it more potently boosted both CD4

and CD8 T cell immunity in previously BCG-vaccinated volunteers. While we found low to medium levels of pre-existing anti-Ad5 humoral immunity in most of the trial volunteers, there was no evidence that pre-immunization anti-Ad5 immunity significantly dampened the potency of Ad5Ag85A vaccine.

The clinical studies carried out in non-human primates and human volunteers with MVAAg85A vaccine have shown that the T cells activated by parenteral and respiratory mucosal routes of vaccination are less compartmentalized than in rodents. In rodent studies, parenteral genetic vaccination does not induce detectable T cell responses within the respiratory tract, in contrast to the respiratory mucosal route of delivery. Although in primates and humans, intradermal delivery triggered detectable T cell responses within the respiratory tract, these studies in non-human primates and humans support that respiratory mucosal route of vaccination induces greater levels of local immune responses.

Comparing IM with respiratory mucosal route in our trial will allow us to examine whether the same is true with Ad5Ag85A TB vaccine, and it also provides us the opportunity to examine the mechanisms for differential T cell homing following parenteral and respiratory mucosal routes of vaccination.

Similar to the MVAAg85A aerosol trial [17], our trial will focus only on BCG+ human volunteers. Ad5Ag85A vaccine is being developed as a booster to be used following BCG prime vaccination in humans. Our completed phase 1 intramuscular trial found Ad5Ag85A vaccine to be equally safe in BCG+ human volunteers as in BCG- humans [36]. Furthermore, BCG+ humans had similar levels of pre-existing anti-Ad5 humoral immune responses as in BCG- humans.

1.3.5 Our clinical experience in using recombinant replication-deficient adenoviral vaccine in cancer patients

The McMaster Immunology Research Centre also has experience in planning and executing clinical trials involving the use of recombinant replication-deficient adenoviral gene vectors in cancer patients. A phase II trial evaluating the clinical efficacy of multiple injections of Ad5gp100-DC vaccinations in patients with stage IV metastatic melanoma has been completed. Phase I trials investigating the safety and comparative efficacy of a CD34+ DC vaccine transduced with an adenoviral vector expressing rat Her-2/neu [Ad5rHer-2] and the direct injection of Ad5rHer-2 in stage IV breast cancer have also been completed. There were no grade 3 or 4 adverse effects reported that were related to the vaccine administration and the safety and tolerability profile of these adenoviral gene vectors was good.

1.3.6. Potential side effects associated with the use of adenoviral vector for the purpose of vaccination

Adenovirus gene therapies have been studied in multiple human trials and have been found to be well tolerated and associated with minimal toxicity. In trials using doses as high as $1x10^{11}$pfu administered subcutaneously, the most common side effects have been mild fever, chills,

shivering, local pain and diarrhea [112]. A 2002 review of completed trials using adenovirus vectors concluded that intratumoral injections of up to $1x10^{12}$ particles is safe [112].

In 1999, adenovirus-based gene therapy underwent scrutiny after the death of an 18 year old patient with ornithine-transcarbamylase (OTC) deficiency, four days after the administration of an Ad vector carrying a functional OTC gene at the University of Pennsylvania. A very high dose ($3.8x10^{13}$ particles) of Ad vector was administered directly into the hepatic artery. The high virus load caused the activation of a systemic immune response that resulted in cytokine release leading to the intravascular coagulation and death.

Unlike adenoviral-mediated gene replacement therapy, which entails the use of large quantities of vector and its direct delivery into the target tissues, often vital organs as the lung and liver, adenoviral-mediated vaccination requires relatively small doses of vector and can be conveniently carried out at peripheral sites. Local tissue inflammatory response is the commonly expected side effect.

There is no evidence that Ad5 particles can induce inflammation in the brain if administered into the nose and respiratory tract. Animal experiments administering Ad5 vectors to the nasal cavity of mice have not shown any gene expression beyond the olfactory bulb and even if a small number of Ad5 particles did enter the brain, adenovirus is not known to cause encephalitis and the non-replicating vector is not expected to induce any local inflammation [100, 113]. There are no reports of any adverse neurological events from the human studies of Ad5 reported to date. When vaccine is delivered to the respiratory tract or when memory immune cells respond to infection, damage to the lung is a potential side-effect [114] but has not been demonstrated in animal models following immunization or challenge, nor in any human studies reported to date. An Ad5 influenza vaccine administered intranasally to 24 healthy adult volunteers was safe and well tolerated [46]. Two repeated immunizations, 28 days apart, of 10^8, 10^9 and 10^{10} particles (vp) of Ad5-vectored pandemic flu vaccine administered intranasally to healthy human volunteers in a recently completed Phase 1 study, were found to be safe and well tolerated even with the highest dose of vp [47].

1.3.7 Consideration of pre-existing anti-Ad5 immunity that may limit the use of Ad5 vectors

A high incidence of pre-existing anti-Ad5 immunity may limit the utility of Ad5 vectors for genetic immunization. However, the presence of neutralizing antibodies may not be a major limitation to Ad-mediated gene transfer and subsequent antigen-specific immune activation when Ad5 vector is inoculated into a discrete tissue site such as the respiratory tract, likely due to the limited number of antibodies present in the interstitial space [115]. Mucosal vaccination may allow an Ad5 vectored vaccine to bypass pre-existing Ad5 immunity without losing potency and there is evidence from a non-human primate study of an adenovirus-based Ebola virus vaccine that this occurs [115].

In our recently completed phase I study, while we found significant levels of pre-existing anti-Ad5 humoral immunity in many of the trial volunteers, there was no evidence that pre-immunization anti-Ad5 immunity significantly dampened the potency of Ad5Ag85A vaccine nor was the level of Ad5 antibody associated with increased adverse effects.

Subjects with any level of Ad5 antibody will be enrolled in this study.

1.4 RESPIRATORY DELIVERY OF VACCINES.

Respiratory delivery of vaccine is expected to provide the most effective protection from infection such as TB [22, 116-118]. It also addresses the 2nd Grand Challenge, identified by the Bill and Melinda Gates Foundation, of developing the needle-free delivery of vaccines. While much research on respiratory TB vaccination has been carried out in experimental models and in a non-human primate model, little has been realized in human studies except the recently completed trial to evaluate inhaled aerosol-delivered MVAAg85A vaccine [16, 119].

Ad5Ag85A has been evaluated in a non-human primate study. Our data suggest the intratracheal or aerosol delivery of a 10^9 pfu dose to BCG-primed rhesus monkeys to be safe and immune protective as judged by chest x-ray examination and improved survival rates [120].

Much can also be learnt from other vaccines. FluMist® and measles vaccines have been safely administered to humans by intranasal delivery or inhaled aerosol delivery via a mouth-piece [46, 121-124]. Measles vaccine delivered by the respiratory route was reported to be at least as immunogenic as subcutaneous administration [123], although these results were not replicated in a more recent randomized trial [124], and inconsistent results were reported with intranasal delivery [124]. Interest in respiratory delivery of measles vaccine is high and the WHO has established its Measles Aerosol Project with the aim of licensing an inhaled aerosol technology for measles vaccination [125].

For our study the aerosol route has been chosen, based on experimental evidence that mucosal immunization with Ad-based vaccine triggers the most effective immunity via inducing both innate immune memory [126] and T cell immunity in the lung [10] against mucosal infections such as M.tb. Furthermore, in cattle, intranasal inoculation of M. bovis induces disease mainly in the upper respiratory tract and associated lymph nodes, while endobronchial inoculation induces disease mainly in the lower respiratory tract and associated lymph nodes [127]. TB is primarily a lower respiratory tract infectious disease in humans. It is expected that delivering the vaccine by aerosol is more likely than the intranasal route to induce the desired immune responses and protection in the lungs. Furthermore, it has been previously shown that Ad5 vector when delivered intranasally to mice may travel retrograde to the olfactory bulb [113]. Whether this may happen in humans remains unclear although Ad5 vectored flu vaccine delivered intranasally in human trials was found to be safe. Thus, aerosol delivery of Ad5Ag85A vaccine which bypasses the nasal passage, is considered to be safer than intranasal delivery. Thus administration of the vaccine by aerosol into the lungs was chosen for our planned study.

1.4.1 Choice of nebulizer for the aerosol delivery of vaccine and evaluation of Aeroneb® Solo for delivering vaccine

When vaccine is administered as an aerosol, particles are deposited in the respiratory tract depending on particle size [128]. Small particles, less than 5 microns, are deposited throughout the respiratory tract reaching as far as the lower airways and are optimally exposed to the abundant intraepithelial dendritic cells and alveolar macrophages.

The Aeroneb® Solo Vibrating Mesh Nebulizer, a designed as a micropump nebulizer, generates aerosol particles with mass median aerodynamic diameter (MMAD) of 3.4µm ensuring effective lung deposition and is more efficient for drug delivery compared with jet or ultrasonic nebulizers [129, 130]. With the use of the Aeroneb® Solo Vibrating Mesh Nebulizer, the residual volumes are low, maximizing the amount of vaccine delivered to the lung. The Aeroneb® Solo is a single patient use device that has been used in adult and pediatric patients to deliver therapeutic drugs to the lung and is being used in clinical studies to deliver measles vaccine by the aerosol route. In a study evaluating measles vaccine potency in aerosol outputs from nebulizers, reliable laboratory methods were developed to assess vaccine potency and the vaccine remained robust during aerosolization with the Aeroneb® Solo Vibrating Mesh Nebulizer [131].

We performed an *in vitro* evaluation of the Aeroneb® Solo Vibrating Mesh Nebulizer for the delivery of Ad5Ag85A vaccine (see Appendix 8 for the image of the Aeroneb® Solo Mesh Nebulizer administration, details of the laboratory characterization of aerosol delivery of Ad5Ag85A and selected references). We established that a fill volume of 0.5ml was appropriate for nebulization and could be administered comfortably to subjects over a period of approximately 2 minutes. We estimated the amount of vaccine available at the mouth as approximately 50% of the loaded dose and confirmed the size characteristics of the aerosol particles, with 85% of droplets being <5.39 µm in size allowing vaccine deposition below the larynx and into peripheral airways. We estimated the rate of viable virus recovered from the aerosol droplets generated by the Aeroneb® Solo at approximately 20%. No significant aggregation of particles (< 1%) was observed.

2 OBJECTIVES

2.1 PRIMARY OBJECTIVE

1. To evaluate the safety of a single administration of one of two doses of a recombinant replication-deficient human adenoviral (Ad5) TB vaccine (Ad5Ag85A) delivered to the respiratory tract by aerosol in healthy human subjects with a history of BCG vaccination.

2.2 SECONDARY OBJECTIVES

1. To compare the safety and immunogenicity of inhaled Ad5Ag85A administered by aerosol with a single dose of 10^8 pfu administered by intramuscular injection in healthy subjects.
2. To evaluate the effect of pre-existing anti-adenoviral antibodies on the safety and immunogenicity of Ad5Ag85A.

3. STUDY DESCRIPTION

This is an open-labeled phase 1 single institution trial comparing a single administration of one of two doses of recombinant genetic TB vaccine Ad5Ag85A administered by aerosol with intramuscular injection of vaccine in healthy subjects with a history of BCG vaccination.

3.1 STUDY SITE

The study will be carried out at McMaster University Medical Centre, Hamilton, Ontario, Canada

4. STUDY POPULATION

4.1 NUMBER OF PATIENTS

Up to 36 healthy subjects.

All subjects will receive active vaccine. As each subject serves as his/her own control (before and after vaccination) for this Phase I safety and immunogenicity study there is no requirement for a placebo group. Any participant who drops out before receiving vaccination or completing at least one post-vaccination bronchoscopy will be replaced; additional participants will be enrolled to adjust for participants randomized to receive the higher dose vaccine by aerosol, but who do not complete the week 8 bronchoscopy. Those who receive vaccination but do not complete the post-vaccination bronchoscopies will be asked to continue in the study to contribute to the safety data.

4.2 SELECTION CRITERIA

Inclusion Criteria

Subjects who meet all of the following criteria are eligible for enrolment:

1. Healthy human subjects who are between 18 and 55 years of age with a history of BCG vaccination.
2. HIV antibody negative
3. Able to understand and comply with protocol requirements and instructions; able to attend scheduled study visits and complete required investigations.
4. For women, negative pregnancy test and for those women of child-bearing potential practising two acceptable forms of contraception for the duration of the study (barrier contraceptive, hormonal contraceptive (for at least one menstrual cycle before study entry), surgically sterile, post-menopausal 2yrs, abstinence). A barrier contraceptive is a compulsory second method of contraception for those female subjects who are of child-bearing potential but does not apply to those women who are surgically sterile or post-menopausal >2 years or practicing abstinence. Barrier contraception would not be considered an appropriate second method of contraception for women of child-bearing potential who are practicing abstinence. Subjects who report sexual abstinence will need to be using a second method of contraception (hormonal method or intrauterine device) but will also be counseled about the need for barrier methods of contraception should abstinence be no longer practiced.
5. For men, using barrier contraception for the duration of the study

Exclusion Criteria

Subjects who meet any of the following criteria are not eligible for admission to the study:

1. Pregnant or lactating women
2. Subjects who have any acute or chronic illnesses including active tuberculosis, any relevant findings on physical examination or are receiving any drug treatment in the opinion of the

investigator likely to affect the immune system (see Appendix 3 for list of drugs) including current use of inhaled or nasal steroids.

3. Subjects with a history of any bleeding disorder or receiving any drug treatment that in the opinion of the investigator may increase the risk of bleeding
4. Subjects with a history of respiratory disease, e.g. asthma, chronic bronchitis, COPD
5. Current cigarette smokers, current e-cigarette smokers and ex-smokers who have quit less than a year ago, as reported by the subject.
6. Subjects with clinically significant abnormality of baseline spirometry tests: FVC <90% predicted; FEV_1 <80% predicted; FEV_1/FVC ratio <80% or DLCO <70% predicted.
7. Any health-related condition for which study bronchoscopy is contraindicated
8. Subjects who have a history of active or latent TB infection or whose PBMCs are responsive to ESAT6/CFP10 stimulation using a commercial interferon gamma release assay for TB (Quantiferon®-TB GoldPlus Test) [consistent with latent TB infection].
9. Subjects whose baseline laboratory values are outside of the normal range, as shown in Appendix 1 unless the abnormality is considered not to be of clinical relevance by the investigator. A single repeat test is allowed during the screening period.
10. Subjects whose use of alcohol or drugs would, in the opinion of the investigator, interfere with adherence to the study protocol.
11. Subjects who are using, or have a history of using, inhaled cocaine, metamphetamine or other inhaled or smoked recreational drugs. Subjects who give a history of smoking marijuana more than a year ago may be enrolled as long as they agree not to smoke marijuana for the duration of the study.
12. Failure to provide written consent.
13. Known allergy to vaccine components
14. Previous vaccination with Ad5Ag85A or any other experimental TB vaccine
15. Known exposure to active TB within past 6 months or subjects whose occupation puts them at increased risk of TB exposure (based on Hamilton Health Science/St Joseph Healthcare list of high risk personnel)
16. Any abnormality on chest x-ray suggestive of active or remote tuberculosis infection or evidence on chest-x-ray of clinically significant respiratory disease.
17. PPD skin test within last 12 months

4.2.1 Indications for a delay in enrolment/vaccination

1. Subjects with symptoms suggestive of an upper respiratory tract infection (including cough, runny nose, or sore throat) within the past 4 weeks
2. Subjects who have received any blood product or immunoglobulin infusion within the past 3 months
3. Receipt of any vaccine within past 30 days or planned vaccination within 21 days after receiving the experimental TB vaccine. Intranasal administration of FluMist® is contraindicated for the duration of the study.

4.2.2 Participants previously screened and found ineligible because they did not meet the criteria of being able to attend scheduled study visits and complete required investigations, may be rescreened should their availability change.

5. STUDY PROCEDURES

5.1 STUDY OUTLINE

36 subjects will be enrolled as follows:

Aerosol doses (n=22) or IM administration (n=14)

The first cohort of 8 BCG positive subjects will receive 10^6 pfu Ad5Ag85 vaccine administered using the Aeroneb® Solo Vibrating Mesh Nebulizer. Assuming acceptable safety outcomes, the second cohort will consist of 28 subjects randomly allocated to receive $2x10^6$ pfu by aerosol (n=14) or 10^8 pfu by single IM administration (n=14).

With the lower aerosol dose, we will first vaccinate 2 subjects. Assuming no grade 3 or 4 adverse events or any serious adverse events at least possibly associated with vaccine in subjects who have been followed for 2 weeks after administration of vaccine, we will proceed to immunize the rest of the subjects in this dose group. Reports detailing all AEs and SAEs for 4 weeks post-dose will be reviewed by the Safety Monitoring Committee (SMC) following the first dose cohort. We will not move to the next dose group until the conditions are met as described in 6.4.1.

For the second cohort, a randomization list will be generated containing sequential codes linked to route of study vaccine assignment, either IM injection or aerosol. The codes will be assigned to subjects in the order in which they are enrolled. Once the subject has completed the screening examination and is eligible for vaccination, the research co-ordinator will access a password protected computer site for group allocation.

Additional participants will be enrolled into the second cohort to adjust for participants who do not complete the week 8 bronchoscopy, until at least 6 participants have received the higher dose of vaccine by aerosol and completed the 2 and 8 week bronchoscopies.

Enrolment into the IM cohort will be discontinued once at least 9 participants have been randomized to the IM route, received vaccine and completed at a minimum the week 2 bronchoscopy (see Amendment 3 and Appendix 9)

Following completion of enrolment in the higher dose aerosol cohort, an additional three participants (with additional participants enrolled to adjust for participants who do not complete the week 2 and 8 bronchoscopies) will be enrolled into the low dose aerosol cohort (See Amendment 3 and Appendix 9)

If the SMC recommends discontinuation of further aerosol vaccinations or no escalation to the next dose of aerosol vaccine, enrolled subjects who have received aerosol vaccine will continue to be followed, according to schedule.

Subjects will be followed for a total of 24 weeks after vaccine administration.

5.1.1 Justification for dosing regimen, route of administration and study design

Because of the limited data from animal models and the difficulty extrapolating the pre-clinical results to human respiratory vaccine delivery due to differences in anatomy, respiratory immunologic tissues and responses to infecting organisms in humans and animals, a cautious dose escalating approach in human subjects will be employed in this study.

The minimum dose chosen is two logs lower than the dose used in our Phase 1 study and a log lower than the dose of MVAAg85A administered to BCG vaccinated subjects using an Omron nebulizer in the recently completed aerosol study [17].

Based on the safety and immunogenicity seen with the lower dose, a dose of $2x10^6$ pfu has been selected for the aerosol dose of the second cohort and will allow further evaluation of vaccine safety and immunogenicity (see appendix 8 for preliminary safety and immunogenicity data). Much higher aerosol doses (10x and 100X) are unlikely to be associated with significant increases in immunogenicity, but may be associated with an increase in adverse events and immune cell exhaustion. Assuming no dose-limiting toxicities, the dose of $2x10^6$ pfu by aerosol will be compared with 10^8 pfu administered i.m, the dose in our completed phase 1 study of Ad5Ag85A that was shown to be safe and well-tolerated in BCG+ humans [36]. There were no vaccine-related serious adverse events. The most common adverse effect was mild to moderate local reaction at the injection site and mild systemic reactions e.g. headache and malaise, which resolved in 24 hours. This dose was robustly immunogenic in BCG+ healthy volunteers.

In our completed Phase 1 IM study, immunological responses were significantly higher in the BCG positive group, with no evidence of increased toxicity. In this study we will compare the safety and immunogenicity of vaccine by route of administration (IM or aerosol) and dose in BCG positive individuals. For the second cohort, subjects will be randomly allocated to IM injection or the aerosol route to avoid selection bias.

5.2 SUBJECT SELECTION

Volunteers will be recruited by advertising, approved by the local REB. A patient recruitment coordinator will be recruited to identify potential subjects, and will include visiting potential recruitment sites, conducting information sessions and preparing advertising.

Human volunteers who have consented to the study and signed the consent form will have screening hematologic and biochemistry tests, an interferon gamma release assay for TB, HIV testing and chest-x-ray done. A full medical history and physical examination will be completed and spirometry performed.

A history of BCG vaccination will be recorded (based on patient/parent recall, vaccination records, presence of scar or birth country where BCG vaccine is routinely administered).

Subjects will be considered as BCG(+) based on patient/parent recall, vaccination records, presence of scar or birth country where BCG vaccine is routinely administered. Absence of a

scar is unreliable as a significant proportion of individuals will not develop a scar following BCG[132]. For individuals born outside of Canada, The BCG World Atlas will be used to confirm whether BCG had been routinely administered at birth. Subjects whose BCG positive status cannot be determined will be excluded from the study.

Subjects whose PBMC's are responsive to ESAT6/CFP10 stimulation, using a commercial interferon gamma release assay for TB (Quantiferon®-TB Gold Plus test) will not be enrolled; the response as recommended by the manufacturer for a positive result will be used as we did in our completed trial [36].

Subjects excluded because of possible latent TB (reactive interferon gamma release assay for TB), abnormal chest x-ray, clinical history suggestive of active TB, abnormal spirometry or clinically significant laboratory abnormalities will be referred for medical evaluation.

Investigations		Timing (Prior to vaccination)
History, physical examination	History of TB and BCG vaccination. Past medical history, current medications.	Within 56 days
Hematology	CBC, automated differential, platelet count	Within 56 days
Spirometry and DLCO	FEV_1, FVC, FEV_1/FVC ratio; DLCO	Within 56 days
Chemistry	Na, K, Creatinine, AST, alkaline phosphatase (ALP), bilirubin,	Within 56 days
Blood pregnancy test		Within 56 days
Chest x-ray	Evidence of active or remote tuberculosis infection or clinically significant respiratory disease	Within 3 months
Viral serology	HIV antibody	Within 56 days
Test for latent TB	M tb antigen stimulation using commercial interferon gamma release assay (Quantiferon®-TB Gold Plus test)	Within 56 days

5.3 IMMUNOLOGIC EVALUATIONS PRIOR TO VACCINATION

Eligible subjects will have samples collected:

- ♦ To determine the titer of anti-human type 5 adenovirus neutralizing antibodies both in the BAL fluids and peripheral blood samples (sera). Subjects with any level of serum Ad5 antibody or who have no detectable Ad5 antibodies will be enrolled and the effect of Ad5 neutralizing titres on safety and immunogenicity will be analysed.
- ♦ To quantify the Ag85A-specific T cells in the peripheral blood and bronchial lavage fluid. Whole blood, PBMCs and bronchial lavage fluid will be obtained and subjected to *M.tb* antigen stimulation and cytokine production measurement and IFN-γ Elispot assay to determine the level and quantity of antigen-specific T cells [Table II–Baseline examination]. The relative number and the phenotypes of CD4 and CD8 T cells will be determined by FACS analysis. Macrophages will also be stimulated and their phenotype and gene expression examined. The *M.tb* antigens to be used include crude *M.tb* antigens (*M.tb*CF),

recombinant Ag85A protein (rAg85A), pooled Ag85A overlapping peptides. Unstimulated, mock-stimulated and polyclonal-stimulated samples will be set up in parallel as background and positive controls. Unused PBMC samples will be stored by cryopreservation in liquid nitrogen. 30-40 ml of blood will be taken from each subject.

- *Bronchoscopy* will be performed using a flexible bronchoscope, with the procedure performed in the research bronchoscopy facility at the Health Science Centre, McMaster University by a respirologist experienced in this technique 1 to 6 days before planned vaccination. Briefly, after the upper airway and nasal passage has been anesthetized, the bronchoscope is inserted nasally or orally and the airways inspected. To perform a modified bronchoalveolar lavage, the bronchoscope is advanced until it is wedged in the right middle bronchus and a total of 160 ml of sterile saline are instilled in 4x40 ml separate aliquots and then aspirated back. Should there be any anatomic variation that precludes using the right middle lobe, the bronchoscopist will choose an alternative site. All four aspirates are kept separately for analysis, aiming for a total volume of 80-100 ml. It is estimated that this volume will provide a total of 10-15 million cells. (See Appendix 6)
- Collected serum and lavage fluids will also be measured for anti-Ag85A IgG and IgA antibodies and levels of selected chemokines and cytokines.

5.4 VACCINE ADMINISTRATION

Subjects will be eligible for vaccination if their screening investigations are within the normal range and the HIV antibody test is negative. Women of child-bearing potential will not receive vaccination until it is known that the urine pregnancy test done on the day of vaccination is negative.

Eligible subjects will be randomly allocated to either IM or aerosol administration of vaccine as outlined in the details of study outline (5.1).

For each subject allocated to receive vaccine by aerosol, a single dose of Ad5Ag85A diluted in 0.5ml saline will be aerosolized using the AeroNeb Solo Vibrating Mesh Nebulizer and inhaled via mouthpiece using tidal breathing. For subjects allocated to the IM route, Ad5Ag85A diluted in 0.5ml saline will be injected to the deltoid muscle of the right arm contralateral to the arm of previous BCG vaccination where this is known. All subjects receive active vaccine and will be observed for 60 minutes after vaccine administration.
See Section 6 for details about the investigational product

5.5 FOLLOW UP PROCEDURES

Subjects will be asked to record their temperature twice a day daily at set times for 5 days and keep a diary and record any symptoms they may experience for 14 days after the administration of vaccine. Subjects will be specifically asked to record any symptoms of cough, wheeze, shortness of breath, itchy eyes, rash or dry skin, dry mouth, sore throat, headache or any gastrointestinal symptoms. Subjects who receive IM injections will be asked to record any injection site reactions.

Between 48 and 72 hours after the administration of the vaccine, or if they experience any symptoms, subjects will be contacted for follow-up, seen in the clinic if necessary and undergo a physical examination and any new symptoms identified and as necessary managed clinically.

Follow-up investigations will be performed as per Study Flow chart (See Appendix 2). Investigations may be performed within 3 days of the scheduled visit at 2 and 4 weeks, and within 7 days of the visit at 8, 12, 16 and 24 weeks.

- *Bronchoscopy:* Bronchoscopy will be repeated at 2 and 8 weeks after immunization. An interval of two to eight weeks between bronchoscopies is a safe interval at which we would have our volunteers undergo the repeat procedure.

- *Examination of immune responses:* Mononuclear cells will be isolated from the bronchial lavage fluid at baseline, 2 and 8 weeks post-vaccination and fresh PBMC's will be isolated from heparinized blood collected at baseline, 2, 4, 8 and 12 weeks post-vaccination. An interferon-γ Elispot assay will be performed to determine the level and quantity of antigen-specific T cells. Antigens used for stimulation of these cells will include an M.tbCF, recombinantAg85A protein and pools of 7-10 overlapping Ag85A peptides. Unstimulated, mock-stimulated and polyclonal-stimulated samples will be set up in parallel as background and positive controls.

  Cytokine production ELISA will be performed in supernatants collected from bronchial lavage fluids and overnight whole blood cultures with and without antigenic stimulation.

  Intracellular cytokine staining (ICS) will be performed in whole blood or PBMC and the mononuclear cells from the bronchial lavage. Following incubation in the presence of anti-CD28 and anti-CD48d antibodies, cells will be stimulated with M.tb CF, a pool of Ag85A peptides or PHA (positive control), then stained for T cell markers CD3, CD4, CD8 and other selected surface molecules and cytokines. The stained cells will be analyzed by flow cytometry. Gene expression will be examined by RNAseq with mononuclear cells from the bronchial lavage at baseline and week 8.

- *Measurement of adverse events:* Adverse events will be assessed according to the CTCAE Expanded Common Toxicity Criteria (Appendix I). Fever is defined as ≥37.9°C (non-axillary) and the intensity of fever will be categorized according to the NCI Expanded Common Toxicity Criteria.

5.6 SUBJECT DISCONTINUATION

Subjects have the right to withdraw from the study for any reason. The investigator has the right to remove patients from the study for non-adherence with study visits.

Subjects who received the vaccine but withdraw from the study or are withdrawn from the study will be invited to attend for an end-of-study visit and asked to continue to provide safety data. Physical examination and history will be performed and subjects asked to provide end-of-study laboratory specimens for safety and immunologic testing. Subjects who withdraw consent for any further study visits will be asked to consent to telephone contact from the study coordinator at 2, 4, 8, 12, 16 and 24 weeks post vaccination to assess for any adverse events and requested to contact the study investigator if any adverse events occur; this will be documented in the case record. Subjects who drop out before completing at the two week post-vaccination bronchoscopy or do not receive vaccine will be replaced. Additional participants will be enrolled to adjust for participants randomized to receive the higher dose vaccine by aerosol, but who do not complete the week 8 bronchoscopy.

Subjects whose screening tests at the screening visit are outside the normal range (after re-screening) will not proceed to receive vaccine, but will be withdrawn from the study and have no further study visits. Appropriate medical referral will be made to follow-up on any abnormalities detected at the screening visit.

6. INVESTIGATIONAL PRODUCT

6.1 VACCINE DOSING AND ADMINISTRATION

Clinical grade Ad5Ag85A will be provided by the Robert E Fitzhenry Vector Laboratory, McMaster Immunology Research Centre, McMaster University, Hamilton, Ontario, Canada. Ad5Ag85A is produced according to current Good Manufacturing Practices (cGMP) in the Vector Laboratory, and has been fully certified.

Ad5Ag85A is a recombinant type 5 human adenovirus vector which has been engineered to express Ag85A. The adenovirus construct is E1 and E3 deleted. Ad5Ag85A consists of the MCMV promoter (start: 1724, end: 2251); tPA signal sequence (start: 1573; end: 1676); Ag85A (start: 685; end: 1572) and SV polyA signal (start: 483, end: 641).

Two doses ranging $1x10^6$ plaque forming units and $2x10^6$ of Ad5Ag85A will be evaluated by aerosol administration. For aerosol administration, vaccine will be diluted in 0.5 ml saline and administered by aerosol using Aeroneb® Solo single patient use vibrating mesh nebulizer over approximately 2 minutes. Aerosol will be administered in a room with negative pressure ventilation or room with portable HEPA filtration. Staff administering the vaccine must wear gloves, gowns, eye protection and a fit-tested N95 respirator during the administration of the aerosol.

For IM injection, a single dose of 10^8 will be administered.

6.1.1 Packaging and labeling

Ad5Ag85A is supplied in cryovials, labeled (in both English and French) with the following information:

a) a statement indicating that Ad5Ag85A is for clinical trial use only
b) the name of the drug;
c) the recommended storage conditions for the drug;

d) the lot number of the drug
e) Protocol #
f) Expiry date
g) Name and address of sponsor

6.1.2 Storage, Dispensing and Reconciliation of Study Drug

The vector is to be stored at –70°C or below in a temperature monitored freezer with controlled access. Please see Investigators Brochure for handling and dosage preparation and drug reconstitution and administration instructions.

6.1.3 Dose modification

No dose modifications of the study drug are permitted.

6.2 SAFETY AND PRECAUTIONS

We have previously demonstrated that the IM administration of 10^8 pfu of Ad5Ag85A in BCG-negative and positive subjects is safe. We predict that aerosol-delivered Ad5Ag85A will be well-tolerated by BCG vaccinated healthy human volunteers, but we will monitor closely for any adverse events and increase to the higher dose carefully. With the higher aerosol dose, we will not proceed to complete enrolment in the second cohort until the first 2 subjects who received vaccine by aerosol have been followed for two weeks after administration of vaccine. Assuming no dose-limiting toxicities we will then proceed to randomize and immunize the rest of the subjects in the second cohort. Reports detailing all AEs and SAEs for 4 weeks post-dose will be reviewed by the SMC following each dose cohort. We will not move to the next dose group until the conditions are met as described in 6.4.1. Because some BCG-vaccinated humans may have a small number of mycobacterial antigen-reactive T cells in the lung [133] and a significant number of such cells in the peripheral compartments, aerosol Ad5Ag85A delivery may mobilize systemic BCG-specific T cells into the lung, potentially associated with a localized immune reaction and inflammatory response. Patients will be closely monitored for respiratory symptoms after vaccination.

There is no evidence from previous studies that shedding of replication deficient adenovirus will occur or that transmission of recombinant virus from the subject to family members or associates is a risk following administration (see additional information 6.5.

Bronchoscopy is a safe procedure with reported complication rates ranging from 0.8 to 1.08%. The most common complications include transient hypotension and syncope, bronchospasm, hypoxia, epistaxis, nausea and laryngospasm. Major complications are uncommon and usually associated with pre-existing cardiac or pulmonary disease. Repeat bronchoscopy after an interval of two to eight weeks is safe [134]. Respirologists from McMaster University Department of Medicine are experienced in and routinely perform these procedures on patients and research subjects.

Biohazard Precautions

All persons must wear masks, gloves, gowns and a fitted N95 respirator at the time of administration of aerosol vaccine and masks and gloves for the administration of IM vaccine. Hand washing must be performed after contact, even when gloves have been worn. All disposable materials will be discarded into biohazardous waste and disposed according to standard procedures. Any needles and syringes will be transported back to the level II facility where they will be bleached and discarded. Trial recruits will receive an information sheet describing any necessary precautions.

6.3 SAFETY MONTORING COMMITTEE

A 3-person Safety Monitoring Committee (SMC), independent from the investigators and the clinical trial site, consisting of experts in infectious diseases and respirology, will be established to review all adverse events. The SMC's primary responsibility will be to monitor volunteer safety. The SMC will at specified intervals and at least 4 weeks following the last subject enrolled at each dose review unblinded individual and cumulative data for evidence of study-related AE's and all SAE's. The SMC will recommend to the trial sponsor and collaborating parties moving to the next dose of vaccine or trial discontinuation (See Section 7).

The PI must provide the SMC with:

1. Prompt reports of any SAEs occurring during the study, to include a written report of events, outcomes of the SAE and relationship to the vaccine.
2. Regular reports of AEs, including cumulative reports 4 weeks after the last subject has been vaccinated for each dosing cohort.
3. Any protocol amendments, informed consent changes or revisions of other documents originally submitted for review.
4. Any new information that may affect adversely the safety of the subjects or the conduct of the study.

Within 14 days of discontinuation of further vaccinations because of dose limiting toxicities (Section 6.4.1), the SMC will be convened and include the PI and co-PI's and the study sponsors to review and discuss the safety data. The SMC should come to a consensus decision based on the majority vote to restart vaccine administration or to permanently discontinue further aerosol vaccinations and whether or not to continue to enroll the IM group at the maximum dose reached by the aerosol route.

6.4 SAFETY MONITORING PLAN

Reports detailing all AEs and SAEs for 4 weeks, post-dose will be reviewed by the SMC following each dose cohort. Approval to move to the next group will not proceed until:

1. All subjects in a given cohort have received the dose of vaccine and been followed for at least 4 weeks.
2. The SMC has reviewed the available safety data and recommends further dose escalation
3. No SAE's that are probably or definitely related to the vaccine have occurred
4. No dose limiting toxicities as outlined below have occurred

6.4.1 Dose limiting toxicities

The following dose limiting toxicities will preclude further dose escalation or additional doses within a dose level until reviewed by the SMC:

1. One or more volunteers have experienced a SAE possibly, probably or definitely related to the vaccine, or
2. In one or more volunteers within a dose level, occurrence of Grade 3 or greater AEs judged possibly, probably or definitely related to the study vaccine.

6.5 ADDITIONAL BACKGROUND INFORMATION OF ADENOVIRAL TB VACCINE

6.5.1 Name and Chemical Information

Type 5 human adenoviral vector, replication-deficient, encoding an *M. tuberculosis* antigen Ag85A. This vaccine was produced to clinical grade specifications in our GMP facility. See separate chemical and manufacturing sections provided in the Investigators Brochure.

6.5.2 Chemical Structure

Double stranded DNA virus.

6.5.3 Mechanisms of Action

The virus has been rendered replication deficient and cannot replicate in host cells. Clinical grade Ad5Ag85A has been modified by removing the E1 DNA sequences and replacing them with cDNA for *M. tuberculosis* Ag85A.

6.5.4 Experimental anti-TB Activity

We have evaluated Ad5Ag85A in both small and large animal models following BCG priming. In a murine model of TB, this vaccine demonstrated protective efficacy against pulmonary *M. tuberculosis* challenge. In a bovine model, this vaccine triggered immune activation and protected against pulmonary *M. bovis* challenge. In a goat model, this vaccine enhanced protection from *M. caprae* challenge. In a guinea pig model, this vaccine significantly prolonged the survival of *M. tuberculosis* infected guinea pigs.

6.5.5 Toxicology/Safety Issues

Risks Associated with Recombinant Wild-type or Competent Adenovirus (RCA)
Clinical grade adenoviral vector may contain a minute amount of RCA (<0.0000001%) that is capable of viral replication. The amount of RCA present in the clinical grade Ad5Ag85A produced for this study is ≤ 1 in 10^9 PFU (see Investigators Brochure and details of Safety tests) Although Ad5Ag85A is engineered to be replication deficient and incapable of propagation in the host, data from immune deficient mice suggest that extremely low levels of viral replication and protein expression may occur following injection of replication defective vector. In these instances, E1 proteins which are necessary for transactivation of other regions preceding viral DNA replication, could be provided by host target cells and hence complement the replication defect. In fact, E1 proteins have been detected in tissue samples of bronchial epithelium of

patients with chronic lung disease. There is also an unlikely possibility that vector could be inadvertently administered to a person with an ongoing adenoviral infection. Here, the wild-type virus could provide E1 proteins but again the infection would be mild.

Nevertheless: (1) adenoviral infections are common and except in severely immunosuppressed patients or rare reports associated with adenovirus 14, manifest as self-limited and clinically mild infections. Low levels of RCA or wild-type virus would be cleared by the immune system as is the case when a patient has a common community-acquired upper respiratory tract adenoviral infection. (2) in the situation where occasional host cells may be able to provide E1 proteins (from latent or residual adenovirus) and complement the replication defect, the extent of toxicity will be limited to E1 positive cells only. As soon as the vector transduces a cell that is E1-negative (the majority of host cells) no further propagation will occur.

Risk of a recombination event resulting in a wild-type vector expressing M. tuberculosis Ag85A
If a cell contained the Ad5Ag85A vector and wild type Adenovirus of the same subgroup, a crossover event could occur integrating E1 sequences into the vector or M. tuberculosis Ag85A sequences into the wild-type virus. The resultant viral DNA would be too large for packaging and thus could not be propagated. Only limited M. tuberculosis Ag85A or E1 sequences could be successfully integrated into a virus and these would be eliminated by the host immune system.

Risk of host genomic adenoviral integration
The risk of integration of adenoviral sequences into host genome is extremely low. Adenovectors remain episomal following entry into the nucleus. As they do not replicate, they are not present in cellular progeny. For the purpose of the trial, it is required that women of child-bearing age and men use a barrier method of birth control for a period of at least six months following administration of the Ad vaccine. A pregnancy test will be performed prior to vaccination and at wk 4 post vaccination for women of child-bearing potential. Women who become pregnant during the study will be followed for safety outcomes and asked to consent to be followed to determine pregnancy outcome, but will not have any further investigations (blood for immune responses, BAL and induced sputum) performed.

Risks associated with expression of M. tuberculosis Ag85A
Following inhalation to the lung of Ad5Ag85A, it is expected that epithelial cells of the respiratory tract will be virally transduced to produce Ag85A and to a lesser extent some immune cells in the lung will also be transduced to express Ag85A. Ag85A is a secreted mycobacterial protein which is foreign to humans and thus there will be an immune response generated towards the exposure to this protein. Such an immune response should not pose any risk to the vaccinated host. However, because some BCG-vaccinated humans may have a small number of mycobacterial antigen-reactive T cells in the lung [133] and a significant number of such cells in the peripheral compartments, aerosol Ad5Ag85A delivery may mobilize systemic BCG-activated T cells into the lung which may add to a viral vector-associated inflammatory response. As Ad5Ag85A is replication-deficient, such an inflammatory response is expected to be transient and mild.

The Gene Therapy Discussion Group (GTDG) of the ICH have summarized the principles to be considered to address virus and vector shedding [135] and guidelines have been published by the European Medicines Agency [136] . With respect to replication defective vectors and environmental risk, shedding of RCA has not been detected by PCR. In our previous clinical trials involving the use of Ad5 vectors in cancer patients we have never detected the shedding of vector. Because of the established safety profile, monitoring for viral shedding of the vector has not been required as part of routine monitoring of patients in recent clinical trials of replication defective adenovirus vectors. Detection of shedding of the viral vector by PCR will not be monitored in this study.

Results of Phase 1 safety and Immunogenicity study of intramuscularly injected Ad5Ag85A in humans
In our completed phase 1 IM study, the vaccine was found to be safe and well-tolerated. There were no vaccine-related serious adverse effects [36]. Most common adverse effects were local reactions at the site of injection including pain and redness which were judged mild to moderate and resolved within 24 hrs. One third to half volunteers experienced mild systemic reactions such as headache, fatigue or malaise. One subject reported fever and arthralgia. All systemic reactions were transient in nature and resolved within 24 hrs. There was one serious adverse event (hospitalization for pancreatitis) judged not related to the vaccination. One subject who had a history of food allergy developed grade 2 blood eosinophilia post-vaccination which resolved within 14 days. There were 6 upper respiratory tract infections reported that were judged not related to the vaccination (one was confirmed by PCR to be rhinoenterovirus, and one was probably H1N1 influenza virus infection).

Risks associated with administration of vaccine by aerosol to the respiratory route
In animal studies, no adverse effects have been observed following administration of vaccine via the respiratory route. In the study with calves when Ad5Ag85A was administered by endobronchial inoculation, there were no clinical adverse effects observed.

6.5.6 Pharmacokinetic Studies
No data available.

7. ADVERSE EVENTS AND SERIOUS ADVERSE EVENTS

7.1 ADVERSE EVENT (AE) DEFINITION

An **Adverse Event** is any untoward medical occurrence in a trial volunteer during their participation in an investigational study, whether or not related to vaccine. An AE can therefore be any unfavorable and unintended sign (including an abnormal laboratory finding), symptom, or disease temporally associated with any stage of the study. This includes an exacerbation of pre-existing conditions and intercurrent illnesses. Unchanged pre-existing conditions will not be included as an adverse event unless they represent an exacerbation in intensity or frequency.

7.2 ADVERSE EVENT REPORTING PERIOD

The AE reporting period begins at patient enrolment, after informed consent has been signed and ends after the last study visit. In addition, any known untoward event of any severity that occurs subsequent to the AE reporting period that the Investigator assesses as possibly related to the study therapy (i.e., the relationship cannot be ruled out) should also be reported as an AE.

7.3 ADVERSE EVENT DOCUMENTATION

During the course of this trial, the Investigator must closely monitor patients for the development of AEs. The clinical significance of AEs will be determined by the Investigator and medical intervention will be initiated if required. The following information should be recorded in the patient's medical record and on the AE Case Record Form for such events:

- ♦ Concise diagnosis of the event
- ♦ Onset date
- ♦ Resolution date
- ♦ Severity (worst toxicity grade according to the CTCAE expanded common toxicity criteria)
- ♦ Relation to study therapy

  All AEs will have their possible relationship to study vaccine assessed using the following terms:

  - Definite: Clear-cut temporal association, and no other possible cause.
  - Probable: Clear-cut temporal association and a potential alternative etiology are not apparent.
  - Possible: Less clear temporal association; other etiologies also possible.
  - Remote: Temporal association between the AE and the vaccine or the nature of the event is such that the vaccine is not likely to have had any reasonable association with the observed illness/event (cause and effect relationship improbable but not impossible).
  - Not Related: The AE is completely independent of vaccine administration; and/or evidence exists that the event is definitely related to another etiology

  All local (injection-site) reactions will be considered causally related to vaccination

- ♦ Seriousness (not serious, fatal, life threatening, leads to or prolongs hospitalization, results in persistent or significant disability/incapacity)
- ♦ SAE status (yes or no)

7.4 ADVERSE REACTION TO THERAPY DEFINITION

An **Adverse Reaction to Therapy** is a response to study therapy which is noxious and unintended. Responses to the study vaccine related to any dose should be considered an adverse reaction to study therapy. The phrase "responses to a study vaccine" means that a causal relationship between the study vaccine and the adverse events is at least a reasonable possibility, i.e., the relationship cannot be ruled out.

An **Unexpected Adverse Reaction to Therapy** is an AE, the nature and severity of which is not consistent with the product information contained in the current Investigator's Brochure for the

product. This includes an event that may be symptomatically or pathophysiologically related to the AE listed in the Investigator's Brochure, but differs from the event because of greater frequency, severity, or specificity.

An **Expected Adverse Reaction to Therapy** is an event that is listed in the current Investigator's Brochure. This may include AEs related to the product, but will also include events related to underlying disease or concurrent procedures.
All deaths are unexpected unless the possibility of a fatal outcome from the AE is stated in the Investigator's Brochure.

A **Serious Adverse Reaction to Therapy** is any Adverse Reaction to Therapy that meets the criteria for a Serious Adverse Event.

7.5 SERIOUS ADVERSE EVENT (SAE) DEFINITION

A **Serious Adverse Event** or reaction is any untoward medical occurrence that at any dose of study treatment:

- Results in death
- Is life-threatening
- Requires inpatient hospitalization or prolongation of existing hospitalization
- Results in persistent or significant disability/incapacity
- Is a congenital anomaly/birth defect

Medical and scientific judgment should be exercised in deciding whether expedited reporting is appropriate in other situations, such as important medical events that may not be immediately life-threatening or result in death or hospitalization but may jeopardize the patient or may require intervention to prevent one of the other outcomes listed in the definition above. These should also usually be considered serious.

Any death occurring while the patient is being followed on the study or within 30 days of the last study visit is considered an SAE. Death occurring more than 30 days after discontinuation or completion of study treatment is not an SAE.

7.6 REPORTING OF SERIOUS ADVERSE EVENTS

All SAEs, occurring during study treatment and up to 30 days after the last study visit, must be reported within 24 hours of occurrence. The SAE Form and accompanying source documentation should be submitted. The Research Ethics Board (REB) should be notified about all SAEs that are unexpected and possibly related to study treatment.

In the rare situation that the Investigator does not become aware of the occurrence of an SAE immediately, the Investigator should report the event within 24 hours after hearing of it and document his/her first awareness of the SAE.

Serious adverse events that are unexpected and related to study treatment will be reported to the regulatory authorities, as per ICH Guidelines and Health Canada Adverse Drug Reaction (ADR) Expedited Reporting Summary Form.

SERIOUS ADVERSE EVENTS CONTACTS:
Dr Fiona Smaill, Dr Marek Smieja or Dr Martin Kolb
Telephone 905-521-2100 Ext. 76332
After hours: 905-521-5070

7.7 SERIOUS ADVERSE EVENT FOLLOW-UP

All SAEs must be followed until resolved, became chronic, or stable. Resolution status of such event should be documented on the AE Case Record Form (CRF). In addition, the Investigator must report all SAEs and Unanticipated AEs that involve significant risk to humans promptly to his/her REB.

7.8 SERIOUS ADVERSE EVENT DOCUMENTATION

In addition to the AE CRF, for all events that satisfy criteria for SAEs the following information must be recorded in the patient's medical record and on the SAE Form:

- Patient demographics
- Details of SAE
- Concise description of the event and temporal relation to study therapy
- Treatment given for event and outcome
- Relevant concurrent disease
- Relation to study therapy
- Outcome at time of report
- Toxicity level per common toxicity criteria
- Relevant laboratory tests
- Action taken regarding study therapy
- Concomitant medications
- Concomitant illness

7.9 EXPECTED ADVERSE EVENTS

- Fever
- Chills
- Myalgia
- Cough (including following sputum induction and BAL)
- Wheezing
- Sneezing
- Shortness of breath
- Chest pain
- Headache
- Malaise
- Conjunctivitis
- Rhinitis

- Injection site reaction
- Syncope or light headedness
- Epistaxis

All of these AE's are expected to be no more than mild or moderate.

8. DATA MANAGEMENT AND STATISTICS

All protocol required information will be entered into a source file, reviewed and signed off by the principal investigator or delegate. Data to be collected will detail medical history and screening evaluation, records of physical examinations, vaccination details, concomitant medications, interim medical history taken at each visit and any solicited or unsolicited adverse events. Source documents such as laboratory results sheets and diary cards will be stored in paper files, in a locked filing cabinet.

Volunteers will receive a diary after vaccination, along with instructions on how to use it. These will be reviewed at the 48 hr follow-up visit and collected on day 14 after vaccination. The diary cards represent source documents and will be stored in paper files in a locked filing cabinet.

Source data will be entered into case report forms (CRFs) by clinical research staff. The names, positions, signatures and initials of authorized staff members are documented and will be filed in a signature log in the regulatory binder.

This is a Phase 1 study to establish the tolerability and safety of the administration of vaccine by the aerosol route, the feasibility of enrolling subjects and gathering preliminary immunological data to inform the design of a larger trial. After the first eight subjects have been enrolled and completed at least four weeks follow-up, there will be a preliminary analysis of data and a determination made whether there should be any amendment to the protocol to facilitate enrolment and retention of participants. The primary immunogenicity outcome will be the difference in immune responses between baseline and two weeks following vaccination in the BAL sample. Secondary outcomes will include the change in immune responses at various time points in samples collected by BAL and induced sputum and comparison of the responses from the BAL and the induced sputum samples.

Tabulations and descriptive statistics will be employed in the analysis of all safety and laboratory observations in this study. Toxicities will be tabulated by CTCAE expanded common toxicity criteria.

Wilcoxon matched pairs signed rank test will be used to compare the change of T cell or cytokine responses at various time points from the baseline values within the same vaccination groups. Mann-Whitney U test will be used for comparison of the difference in immunological responses between the doses of vaccine and routes of vaccination. For correlation analysis, Spearman Rank coefficient test will be used. A test significant level of 5% will be used.

8.1 MONITORING

An independent monitor will conduct an initiation visit before the inclusion of the first subject in

the study. The Monitor will verify and document that the investigational team has been properly informed about the trial and regulatory requirements and all regulatory documents are in order. The Monitor will carry out regular follow-up visits. The monitor will carry out a quality control of trial progress: including protocol and operating guidelines, data collection, signature of consent forms, completion of SAE reporting, vaccine management and sample collection and CRF completion. The Monitor will discuss any problem with the investigator and define the actions to be taken. A close-out visit will be performed at the end of the trial. Its goals are to make sure that the centre has all the documents necessary for archiving and all product has been accounted for.

9. ETHICAL, REGULATORY AND ADMINISTRATIVE ISSUES

The study will be conducted according to generally accepted principles of ICH-GCP (International Conference on Harmonization - Good Clinical Practice) guidelines. These guidelines provide assurance that the data and reported results are credible and accurate, and that all rights, integrity and privacy of subjects participating in this trial are protected.

The trial will also be performed in accordance with the recommendations guiding physicians in biomedical research involving human patients adopted by the 18th World Medical Assembly, Helsinki, Finland, 1964 and later revisions or the laws and regulations of the country, whichever provide the greater protection for the individual.

9.1 RESEARCH ETHICS BOARD

Prior to the commencement of the trial, the study protocol must be approved by the local Research Ethics Board (REB) and McMaster University Biosafety Committee. Written informed consent will be obtained from the subject.

9.2 INFORMED CONSENT

The consent form approved by the REB/Ethics Committee must include all elements required by HPB, provincial, and local regulations, as well as any additional elements, relevant to specific study situations, (including a statement that authorities have access to subject records) required by applicable regulations and guidelines.

It is the responsibility of the investigator or designate to give each trial volunteer prior to inclusion in the trial, full and adequate verbal and written information regarding the objective and procedures of the trial and the possible risks involved. The volunteers must be informed about their right to withdraw from the trial at any time. Refusal to participate will involve no penalty or loss of benefits to which the person is otherwise entitled, and will not prejudice medical treatment. It is the responsibility of the investigator or designate to obtain signed informed consent (or witnessed verbal consent according to applicable regulations) from all participants prior to inclusion in the trial.

The patient information and informed consent form to be used must be approved by the same IRB approving the conduct of this study. The following basic elements are included in the consent form:

- A statement that the study involves RESEARCH.
- The PURPOSE of the research.
- A description of PROCEDURES to be followed and identification of any that are experimental.
- Expected DURATION of the patient's involvement in the study.
- Reasonably foreseeable RISKS or discomforts associated with participation in the study.
- The BENEFITS the subject may reasonably expect from participation in the study, including the amount and timing of any payments for study participation.
- A statement that PARTICIPATION IS VOLUNTARY
- Whom to CONTACT if the subject has additional questions about the STUDY.
- Whom to CONTACT about an INJURY associated with the study.
- A statement that describes the extent to which CONFIDENTIALITY will be maintained for records that identify the patient and that notes the possibility that local regulatory agencies may inspect the study records.
- A COMPENSATION statement indicating any available payment for treatment related injury.
- When appropriate, ADDITIONAL INFORMATION relating to:
  - unforeseen risks,
  - additional costs to the patient,
  - how new findings on the experimental treatment will be reported to the subject,
  - the number of subjects in the study or who have previously received the experimental treatment,
  - the possibility of terminating the participation in the study without warning,
  - consequences of a subject's decision to withdraw from the study,
  - procedures for orderly termination of study participation,
  - identification of restrictions or inconveniences (e.g. restrictions on smoking or use of alcohol or over-the-counter medications, the need to avoid pregnancy, performance of pregnancy tests or drug screens, etc.).
- The language in the informed consent must be UNDERSTANDABLE and in no way coercive.

The Investigator must provide the subjects with a copy of the consent form.

9.3 CASE REPORT FORMS (CRFS)

For each subject, source documents will be used to collect data and complete the case report form. The following data will be reported:

- Patient identification
- Subject number
- History
- Physical examination
- Dose and lot number of drug
- Dates of visits and vaccine administration
- Laboratory investigations
- Spirometry
- Adverse events
- Immune tests

9.4 ESTIMATED DURATION OF THE STUDY

This study has an estimated duration of 30 months.

9.5 RETENTION OF SUBJECT RECORDS AND STUDY FILES

The Therapeutic Products Programme of the Health Protection Branch states in its Guidelines for Clinical Trials the following instructions regarding maintenance and retention of study records: "…(a) the sponsor shall record, handle and store all information in respect of a clinical trial in a way that allows its complete and accurate reporting as well as its interpretation and verification, (b) the sponsor shall maintain complete and accurate records to establish that the clinical trial is conducted in accordance with good clinical practices and these Regulations, (c) the sponsor shall maintain complete and accurate records in respect of the use of a drug in a clinical trial…(d) The sponsor shall maintain all records referred to in this Division for a period of 25 years.

9.6 CONFIDENTIALITY

This trial will be conducted in accordance with applicable regulatory requirements for the protection of the privacy and confidentiality of the study subjects.

9.7 NOTIFICATION OF PRIMARY CARE PHYSICIAN

The investigator will notify the patient's primary physician about the patient's participation in the study if the patient has a primary physician and if the patient agrees to the primary physician being informed.

9.8 DISCLOSURE AND PUBLICATIONS

- The results of this study will be published within a reasonable time of its completion.
- The first author will be the trial principal investigator.
- Additional authors will be those who have made a significant contribution to the overall success of the study.

9.9 PROTOCOL AMENDMENTS

All items in this protocol must be followed exactly. If an amendment is required, this must be enacted through a formal documented protocol amendment procedure and must receive approval from all the authorities that approved all protocol recipients with instructions to append then to the protocol, or a revised protocol will be issued.

INVESTIGATOR AGREEMENT

I have read the foregoing protocol "Title of protocol", and agree to conduct the study as described therein.

____
Investigator's name (block letters)

____ _______________________
Investigator's signature Date (dd/mm/yyyy)

10. REFERENCES

1. World Health Organization, *Global Tuberculosis Report 2016.*
2. Andrews, J.R., et al., *Risk of progression to active tuberculosis following reinfection with Mycobacterium tuberculosis.* Clin Infect Dis, 2012. **54**(6): p. 784-91.
3. Corbett, E.L., et al., *The growing burden of tuberculosis: global trends and interactions with the HIV epidemic.* Arch Intern Med, 2003. **163**(9): p. 1009-21.
4. Centers for Disease Control and Prevention. *Trends in Tuberculosis, 2017*. Available from: www.cdc.gov/tb/publications/factsheets/statistics/tbtrends.htm.
5. Vachon, J., V. Gallant, and W. Siu, *Tuberculosis in Canada 2016.* Can Commun Dis Rep, 2018. **44** (3/4): p. 75-81.
6. Menzies, D., et al., *Tuberculosis in health care workers: a multicentre Canadian prevalence survey: preliminary results. Canadian Collaborative Group in Nosocomial Transmission of Tuberculosis.* Int J Tuberc Lung Dis, 1998. **2**(9 Suppl 1): p. S98-102.
7. Brassard, P., et al., *Evaluation of a school-based tuberculosis-screening program and associate investigation targeting recently immigrated children in a low-burden country.* Pediatrics, 2006. **117**(2): p. e148-56.
8. Zumla, A., et al., *Tuberculosis.* N Engl J Med, 2013. **368**(8): p. 745-55.
9. Gallant, V., J. Vachon, and W. Siu, *Tuberculosis drug resistance in Canada 2006-2016.* Canadian Communicable Disease Report, 2017. **43**(11).
10. Jeyanathan, M., et al., *New Tuberculosis Vaccine Strategies: Taking Aim at Un-Natural Immunity.* Trends Immunol, 2018. **39**(5): p. 419-433.
11. Trunz, B.B., P. Fine, and C. Dye, *Effect of BCG vaccination on childhood tuberculous meningitis and miliary tuberculosis worldwide: a meta-analysis and assessment of cost-effectiveness.* Lancet, 2006. **367**(9517): p. 1173-80.
12. Global TB Vaccine Partnership. *Global Report on Tuberculosis Vaccines 2018*. Available from: www.tbvi.eu/wp-content/uploads/2018/02/Summary-SWRTV_Finalproof.pdf.
13. McShane, H., et al., *Recombinant modified vaccinia virus Ankara expressing antigen 85A boosts BCG-primed and naturally acquired antimycobacterial immunity in humans.* Nat Med, 2004. **10**(11): p. 1240-4.
14. Scriba, T.J., et al., *A phase IIa trial of the new tuberculosis vaccine, MVA85A, in HIV- and/or Mycobacterium tuberculosis-infected adults.* Am J Respir Crit Care Med, 2012. **185**(7): p. 769-78.
15. Tameris, M.D., et al., *Safety and efficacy of MVA85A, a new tuberculosis vaccine, in infants previously vaccinated with BCG: a randomised, placebo-controlled phase 2b trial.* Lancet, 2013.
16. White, A.D., et al., *Evaluation of the safety and immunogenicity of a candidate tuberculosis vaccine, MVA85A, delivered by aerosol to the lungs of macaques.* Clin Vaccine Immunol, 2013. **20**(5): p. 663-72.
17. Satti, I., et al., *Safety and immunogenicity of a candidate tuberculosis vaccine MVA85A delivered by aerosol in BCG-vaccinated healthy adults: a phase 1, double-blind, randomised controlled trial.* Lancet Infect Dis, 2014. **14**(10): p. 939-46.
18. Satti I, M.J., Harris SA, Manjaly Thomas ZR, Griffiths K, Antrobus RD, Rowland R, Ramon RL, Smith M, Sheehan S, Bettinson H, McShane H., *Safety and immunogenicity of a candidate tuberculosis vaccine MVA85A delivered by aerosol in BCG-vaccinated*

healthy adults: a phase 1, double-blind, randomised controlled trial. Lancet Infect Dis, 2014. **14**(10): p. 939-46.
19. Lynch, J.P., 3rd, M. Fishbein, and M. Echavarria, *Adenovirus.* Semin Respir Crit Care Med, 2011. **32**(4): p. 494-511.
20. Louie, J.K., et al., *Severe pneumonia due to adenovirus serotype 14: a new respiratory threat?* Clin Infect Dis, 2008. **46**(3): p. 421-5.
21. Centers for Disease Control and Prevention, *Acute respiratory disease associated with adenovirus serotype 14--four states, 2006-2007.* Morb Mortal Wkly Rep, 2007. **56**(45): p. 1181-4.
22. Zhang, J., et al., *Adenovirus-vectored drug-vaccine duo as a potential driver for conferring mass protection against infectious diseases.* Expert Rev Vaccines, 2011. **10**(11): p. 1539-52.
23. Fooks, A.R., et al., *High-level expression of the measles virus nucleocapsid protein by using a replication-deficient adenovirus vector: induction of an MHC-1-restricted CTL response and protection in a murine model.* Virology, 1995. **210**(2): p. 456-65.
24. Alexander, J., et al., *Pre-clinical evaluation of a replication-competent recombinant adenovirus serotype 4 vaccine expressing influenza H5 hemagglutinin.* PLoS One, 2012. **7**(2): p. e31177.
25. Geisbert, T.W., et al., *Recombinant adenovirus serotype 26 (Ad26) and Ad35 vaccine vectors bypass immunity to Ad5 and protect nonhuman primates against ebolavirus challenge.* J Virol, 2011. **85**(9): p. 4222-33.
26. Braucher, D.R., et al., *Intranasal vaccination with replication-defective adenovirus type 5 encoding influenza virus hemagglutinin elicits protective immunity to homologous challenge and partial protection to heterologous challenge in pigs.* Clin Vaccine Immunol, 2012. **19**(11): p. 1722-9.
27. Gomez-Roman, V.R. and M. Robert-Guroff, *Adenoviruses as vectors for HIV vaccines.* AIDS Rev, 2003. **5**(3): p. 178-85.
28. Lubeck, M.D., et al., *Immunogenicity and efficacy testing in chimpanzees of an oral hepatitis B vaccine based on live recombinant adenovirus.* Proc Natl Acad Sci U S A, 1989. **86**(17): p. 6763-7.
29. Prevec, L., et al., *A recombinant human adenovirus vaccine against rabies.* J Infect Dis, 1990. **161**(1): p. 27-30.
30. Santosuosso, M., S. McCormick, and Z. Xing, *Adenoviral vectors for mucosal vaccination against infectious diseases.* Viral Immunol, 2005. **18**(2): p. 283-91.
31. Gowen, B.B., et al., *Extended protection against phlebovirus infection conferred by recombinant adenovirus expressing consensus interferon (DEF201).* Antimicrob Agents Chemother, 2012. **56**(8): p. 4168-74.
32. Gray, G.C., et al., *Respiratory diseases among U.S. military personnel: countering emerging threats.* Emerg Infect Dis, 1999. **5**(3): p. 379-85.
33. Karonga Prevention Trial Group, *Randomised controlled trial of single BCG, repeated BCG, or combined BCG and killed Mycobacterium leprae vaccine for prevention of leprosy and tuberculosis in Malawi.* . Lancet, 1996. **348**(9019): p. 17-24.
34. Buddle, B.M., et al., *Revaccination of neonatal calves with Mycobacterium bovis BCG reduces the level of protection against bovine tuberculosis induced by a single vaccination.* Infect Immun, 2003. **71**(11): p. 6411-9.

35. Santosuosso, M., et al., *Intranasal boosting with an adenovirus-vectored vaccine markedly enhances protection by parenteral Mycobacterium bovis BCG immunization against pulmonary tuberculosis.* Infect Immun, 2006. **74**(8): p. 4634-43.
36. Smaill, F., et al., *A human type 5 adenovirus-based tuberculosis vaccine induces robust T cell responses in humans despite preexisting anti-adenovirus immunity.* Sci Transl Med, 2013. **5**(205): p. 205ra134.
37. Buchbinder, S.P., et al., *Efficacy assessment of a cell-mediated immunity HIV-1 vaccine (the Step Study): a double-blind, randomised, placebo-controlled, test-of-concept trial.* Lancet, 2008. **372**(9653): p. 1881-93.
38. Hammer, S.M., et al., *Efficacy trial of a DNA/rAd5 HIV-1 preventive vaccine.* N Engl J Med, 2013. **369**(22): p. 2083-92.
39. Gray, G.E., et al., *Safety and efficacy of the HVTN 503/Phambili study of a clade-B-based HIV-1 vaccine in South Africa: a double-blind, randomised, placebo-controlled test-of-concept phase 2b study.* Lancet Infect Dis, 2011. **11**(7): p. 507-15.
40. Gray, G.E., et al., *Recombinant adenovirus type 5 HIV gag/pol/nef vaccine in South Africa: unblinded, long-term follow-up of the phase 2b HVTN 503/Phambili study.* Lancet Infect Dis, 2014. **14**(5): p. 388-96.
41. Fauci, A.S., et al., *Immunology. Immune activation with HIV vaccines.* Science, 2014. **344**(6179): p. 49-51.
42. Harvey, B.G., et al., *Cellular immune responses of healthy individuals to intradermal administration of an E1-E3- adenovirus gene transfer vector.* Hum Gene Ther, 1999. **10**(17): p. 2823-37.
43. Harvey, B.G., et al., *Host responses and persistence of vector genome following intrabronchial administration of an E1(-)E3(-) adenovirus gene transfer vector to normal individuals.* Mol Ther, 2001. **3**(2): p. 206-15.
44. Ben-Gary, H., et al., *Systemic interleukin-6 responses following administration of adenovirus gene transfer vectors to humans by different routes.* Mol Ther, 2002. **6**(2): p. 287-97.
45. Harvey, B.G., et al., *Safety of local delivery of low- and intermediate-dose adenovirus gene transfer vectors to individuals with a spectrum of morbid conditions.* Hum Gene Ther, 2002. **13**(1): p. 15-63.
46. Van Kampen, K.R., et al., *Safety and immunogenicity of adenovirus-vectored nasal and epicutaneous influenza vaccines in humans.* Vaccine, 2005. **23**(8): p. 1029-36.
47. Vaxin Inc, *Safety and Immunogenicity study of adenovirus-vectored, intranasal pandemic influenza vaccine.* In: ClinicalTrials.gov Bethesda (MD): national Library of Medicine (US). 2000- [cited 15 March 2013]. Available from: http://clinicaltrials.gov/show/NCT00755703 NLM Identifier: NCT00755703.
48. Chuang, I., et al., *DNA Prime/Adenovirus Boost Malaria Vaccine Encoding P. falciparum CSP and AMA1 Induces Sterile Protection Associated with Cell-Mediated Immunity.* PLoS One, 2013. **8**(2): p. e55571.
49. Ledgerwood, J.E., et al., *A replication defective recombinant Ad5 vaccine expressing Ebola virus GP is safe and immunogenic in healthy adults.* Vaccine, 2010. **29**(2): p. 304-13.
50. Gurwith, M., et al., *Safety and immunogenicity of an oral, replicating adenovirus serotype 4 vector vaccine for H5N1 influenza: a randomised, double-blind, placebo-controlled, phase 1 study.* Lancet Infect Dis, 2013. **13**(3): p. 238-50.

51. St George, J.A., *Gene therapy progress and prospects: adenoviral vectors.* Gene Ther, 2003. **10**(14): p. 1135-41.
52. Rajagopalan, S., et al., *Phase I study of direct administration of a replication deficient adenovirus vector containing the vascular endothelial growth factor cDNA (CI-1023) to patients with claudication.* Am J Cardiol, 2002. **90**(5): p. 512-6.
53. Rajagopalan, S., et al., *Regional angiogenesis with vascular endothelial growth factor in peripheral arterial disease: a phase II randomized, double-blind, controlled study of adenoviral delivery of vascular endothelial growth factor 121 in patients with disabling intermittent claudication.* Circulation, 2003. **108**(16): p. 1933-8.
54. Makinen, K., et al., *Increased vascularity detected by digital subtraction angiography after VEGF gene transfer to human lower limb artery: a randomized, placebo-controlled, double-blinded phase II study.* Mol Ther, 2002. **6**(1): p. 127-33.
55. Grines, C.L., et al., *A randomized, double-blind, placebo-controlled trial of Ad5FGF-4 gene therapy and its effect on myocardial perfusion in patients with stable angina.* J Am Coll Cardiol, 2003. **42**(8): p. 1339-47.
56. Joseph, P.M., et al., *Aerosol and lobar administration of a recombinant adenovirus to individuals with cystic fibrosis. I. Methods, safety, and clinical implications.* Hum Gene Ther, 2001. **12**(11): p. 1369-82.
57. Xing, Z., et al., *Adenovirus-mediated cytokine gene transfer at tissue sites. Overexpression of IL-6 induces lymphocytic hyperplasia in the lung.* J Immunol, 1994. **153**(9): p. 4059-69.
58. Xing, Z., et al., *Transfer of granulocyte-macrophage colony-stimulating factor gene to rat lung induces eosinophilia, monocytosis, and fibrotic reactions.* J Clin Invest, 1996. **97**(4): p. 1102-10.
59. Foley, R., et al., *Adenoviral gene transfer of macrophage inflammatory protein-2 in rat lung.* Am J Pathol, 1996. **149**(4): p. 1395-403.
60. Braciak, T.A., et al., *Overexpression of RANTES using a recombinant adenovirus vector induces the tissue-directed recruitment of monocytes to the lung.* J Immunol, 1996. **157**(11): p. 5076-84.
61. Xing, Z., et al., *Adenoviral vector-mediated interleukin-10 expression in vivo: intramuscular gene transfer inhibits cytokine responses in endotoxemia.* Gene Ther, 1997. **4**(2): p. 140-9.
62. Sime, P.J., et al., *Adenovector-mediated gene transfer of active transforming growth factor-beta1 induces prolonged severe fibrosis in rat lung.* J Clin Invest, 1997. **100**(4): p. 768-76.
63. Xing, Z., et al., *Intradermal transgenic expression of granulocyte-macrophage colony-stimulating factor induces neutrophilia, epidermal hyperplasia, Langerhans' cell/macrophage accumulation, and dermal fibrosis.* Lab Invest, 1997. **77**(6): p. 615-22.
64. Sallenave, J.M., et al., *Adenovirus-mediated expression of an elastase-specific inhibitor (elafin): a comparison of different promoters.* Gene Ther, 1998. **5**(3): p. 352-60.
65. Lei, X.F., et al., *Disruption of antigen-induced inflammatory responses in CD40 ligand knockout mice.* J Clin Invest, 1998. **101**(6): p. 1342-53.
66. Boehler, A., et al., *Adenovirus-mediated interleukin-10 gene transfer inhibits post-transplant fibrous airway obliteration in an animal model of bronchiolitis obliterans.* Hum Gene Ther, 1998. **9**(4): p. 541-51.

67. Lei, X.F., et al., *Compartmentalized transgene expression of granulocyte-macrophage colony-stimulating factor (GM-CSF) in mouse lung enhances allergic airways inflammation.* Clin Exp Immunol, 1998. **113**(2): p. 157-65.
68. Stampfli, M.R., et al., *Adenoviral infection inhibits allergic airways inflammation in mice.* Clin Exp Allergy, 1998. **28**(12): p. 1581-90.
69. Sime, P.J., et al., *Transfer of tumor necrosis factor-alpha to rat lung induces severe pulmonary inflammation and patchy interstitial fibrogenesis with induction of transforming growth factor-beta1 and myofibroblasts.* Am J Pathol, 1998. **153**(3): p. 825-32.
70. Wang, J., et al., *Macrophages are a significant source of type 1 cytokines during mycobacterial infection.* J Clin Invest, 1999. **103**(7): p. 1023-9.
71. Xing, Z., et al., *Adenoviral-mediated gene transfer of interleukin-6 in rat lung enhances antiviral immunoglobulin A and G responses in distinct tissue compartments.* Biochem Biophys Res Commun, 1999. **258**(2): p. 332-5.
72. Stampfli, M.R., et al., *Regulation of allergic mucosal sensitization by interleukin-12 gene transfer to the airway.* Am J Respir Cell Mol Biol, 1999. **21**(3): p. 317-26.
73. Stampfli, M.R., et al., *Interleukin-10 gene transfer to the airway regulates allergic mucosal sensitization in mice.* Am J Respir Cell Mol Biol, 1999. **21**(5): p. 586-96.
74. Jiang, B., et al., *Replication-defective adenovirus infection reduces Helicobacter felis colonization in the mouse in a gamma interferon- and interleukin-12-dependent manner.* Infect Immun, 1999. **67**(9): p. 4539-44.
75. Barbara, G., et al., *Interleukin 10 gene transfer prevents experimental colitis in rats.* Gut, 2000. **46**(3): p. 344-9.
76. Wang, J., et al., *Transgenic expression of granulocyte-macrophage colony-stimulating factor induces the differentiation and activation of a novel dendritic cell population in the lung.* Blood, 2000. **95**(7): p. 2337-45.
77. Lubberts, E., et al., *Intra-articular IL-10 gene transfer regulates the expression of collagen-induced arthritis (CIA) in the knee and ipsilateral paw.* Clin Exp Immunol, 2000. **120**(2): p. 375-83.
78. Lee, W.C., et al., *Contrasting effects of myeloid dendritic cells transduced with an adenoviral vector encoding interleukin-10 on organ allograft and tumour rejection.* Immunology, 2000. **101**(2): p. 233-41.
79. Kolb, M., et al., *Transient transgene expression of decorin in the lung reduces the fibrotic response to bleomycin.* Am J Respir Crit Care Med, 2001. **163**(3 Pt 1): p. 770-7.
80. Xing, Z., et al., *Enhanced protection against fatal mycobacterial infection in SCID beige mice by reshaping innate immunity with IFN-gamma transgene.* J Immunol, 2001. **167**(1): p. 375-83.
81. Santosuosso, M., et al., *Reduced tissue macrophage population in the lung by anticancer agent cyclophosphamide: restoration by local granulocyte macrophage-colony-stimulating factor gene transfer.* Blood, 2002. **99**(4): p. 1246-52.
82. Suga, M., et al., *Transplant immunosuppression enhances efficiency of adenoviral-mediated gene retransfection: inhibition of interferon-gamma and immunoglobin G.* Ann Thorac Surg, 2002. **73**(4): p. 1092-7.
83. Emtage, P.C., et al., *Adenoviral-mediated gene transfer of lymphotactin to the lungs of mice and rats results in infiltration and direct accumulation of CD4+, CD8+, and NK cells.* J Interferon Cytokine Res, 2002. **22**(5): p. 573-82.

84. Wang, J., A. Zganiacz, and Z. Xing, *Enhanced immunogenicity of BCG vaccine by using a viral-based GM-CSF transgene adjuvant formulation.* Vaccine, 2002. **20**(23-24): p. 2887-98.
85. Miller, G., et al., *Endogenous granulocyte-macrophage colony-stimulating factor overexpression in vivo results in the long-term recruitment of a distinct dendritic cell population with enhanced immunostimulatory function.* J Immunol, 2002. **169**(6): p. 2875-85.
86. Chen, Z., et al., *Enhanced HER-2/neu-specific antitumor immunity by cotransduction of mouse dendritic cells with two genes encoding HER-2/neu and alpha tumor necrosis factor.* Cancer Gene Ther, 2002. **9**(9): p. 778-86.
87. Lu, H., Z. Xing, and R.C. Brunham, *GM-CSF transgene-based adjuvant allows the establishment of protective mucosal immunity following vaccination with inactivated Chlamydia trachomatis.* J Immunol, 2002. **169**(11): p. 6324-31.
88. Nochi, H., et al., *Modulation of hepatic granulomatous responses by transgene expression of DAP12 or TREM-1-Ig molecules.* Am J Pathol, 2003. **162**(4): p. 1191-201.
89. Alexander, S., et al., *Protection from endotoxemia by adenoviral-mediated gene transfer of human bactericidal/permeability-increasing protein.* Blood, 2004. **103**(1): p. 93-9.
90. Herold, J., et al., *Transplantation of monocytes: a novel strategy for in vivo augmentation of collateral vessel growth.* Hum Gene Ther, 2004. **15**(1): p. 1-12.
91. Henriksen, P.A., et al., *Adenoviral gene delivery of elafin and secretory leukocyte protease inhibitor attenuates NF-kappa B-dependent inflammatory responses of human endothelial cells and macrophages to atherogenic stimuli.* J Immunol, 2004. **172**(7): p. 4535-44.
92. Wang, J., et al., *Single mucosal, but not parenteral, immunization with recombinant adenoviral-based vaccine provides potent protection from pulmonary tuberculosis.* J Immunol, 2004. **173**(10): p. 6357-65.
93. Barbarin, V., et al., *Pulmonary overexpression of IL-10 augments lung fibrosis and Th2 responses induced by silica particles.* Am J Physiol Lung Cell Mol Physiol, 2005. **288**(5): p. L841-8.
94. Santosuosso, M., et al., *Mechanisms of mucosal and parenteral tuberculosis vaccinations: adenoviral-based mucosal immunization preferentially elicits sustained accumulation of immune protective CD4 and CD8 T cells within the airway lumen.* J Immunol, 2005. **174**(12): p. 7986-94.
95. Carpenter, K.J., et al., *Intrapulmonary, adenovirus-mediated overexpression of KARAP/DAP12 enhances fungal clearance during invasive aspergillosis.* Infect Immun, 2005. **73**(12): p. 8402-6.
96. Malowany, J.I., et al., *Development of cell-based tuberculosis vaccines: genetically modified dendritic cell vaccine is a much more potent activator of CD4 and CD8 T cells than peptide- or protein-loaded counterparts.* Mol Ther, 2006. **13**(4): p. 766-75.
97. Vordermeier, H.M., et al., *Immune responses induced in cattle by vaccination with a recombinant adenovirus expressing Mycobacterial antigen 85A and Mycobacterium bovis BCG.* Infect Immun, 2006. **74**(2): p. 1416-8.
98. Roghanian, A., et al., *The antimicrobial/elastase inhibitor elafin regulates lung dendritic cells and adaptive immunity.* Am J Respir Cell Mol Biol, 2006. **34**(5): p. 634-42.
99. Xing, Z., et al., *Recent advances in the development of adenovirus- and poxvirus-vectored tuberculosis vaccines.* Curr Gene Ther, 2005. **5**(5): p. 485-92.

100. Perez de Val, B., et al., *Goats primed with Mycobacterium bovis BCG and boosted with a recombinant adenovirus expressing Ag85A show enhanced protection against tuberculosis.* Clin Vaccine Immunol, 2012. **19**(9): p. 1339-47.
101. Wang, J. and Z. Xing, *Tuberculosis vaccines: the past, present and future.* Expert Rev Vaccines, 2002. **1**(3): p. 341-54.
102. Xing, Z., *The hunt for new tuberculosis vaccines: anti-TB immunity and rational design of vaccines.* Curr Pharm Des, 2001. **7**(11): p. 1015-37.
103. Santosuosso, M., J. Wang, and Z. Xing, *The prospects of mucosal vaccination against pulmonary tuberculosis*, in *Focus on Tuberculosis Research*, L.T. Smithe, Editor. 2005, Nova Science Publishers Inc: Hauppauge. p. 141-164.
104. McCormick, S., et al., *Manipulation of dendritic cells for host defence against intracellular infections.* Biochem Soc Trans, 2006. **34**(Pt 2): p. 283-6.
105. Feng, C.G., et al., *Priming by DNA immunization augments protective efficacy of Mycobacterium bovis Bacille Calmette-Guerin against tuberculosis.* Infect Immun, 2001. **69**(6): p. 4174-6.
106. Santosuosso, M., et al., *Mucosal luminal manipulation of T cell geography switches on protective efficacy by otherwise ineffective parenteral genetic immunization.* J Immunol, 2007. **178**(4): p. 2387-95.
107. Vordermeier, H.M., et al., *Viral booster vaccines improve Mycobacterium bovis BCG-induced protection against bovine tuberculosis.* Infect Immun, 2009. **77**(8): p. 3364-73.
108. Whelan, A., et al., *Immunogenicity comparison of the intradermal or endobronchial boosting of BCG vaccinates with Ad5-85A.* Vaccine, 2012. **30**(44): p. 6294-300.
109. Xing, Z., et al., *Intranasal mucosal boosting with an adenovirus-vectored vaccine markedly enhances the protection of BCG-primed guinea pigs against pulmonary tuberculosis.* PLoS One, 2009. **4**(6): p. e5856.
110. Yao, Y., et al., *Enhancement of Antituberculosis Immunity in a Humanized Model System by a Novel Virus-Vectored Respiratory Mucosal Vaccine.* J Infect Dis, 2017. **216**(1): p. 135-145.
111. Jeyanathan, M., et al., *Induction of an Immune-Protective T-Cell Repertoire With Diverse Genetic Coverage by a Novel Viral-Vectored Tuberculosis Vaccine in Humans.* J Infect Dis, 2016. **214**(12): p. 1996-2005.
112. Vorburger, S.A. and K.K. Hunt, *Adenoviral gene therapy.* Oncologist, 2002. **7**(1): p. 46-59.
113. Damjanovic, D., et al., *Organ distribution of transgene expression following intranasal mucosal delivery of recombinant replication-defective adenovirus gene transfer vector.* Genet Vaccines Ther, 2008. **6**: p. 5.
114. Hokey, D.A. and A. Misra, *Aerosol vaccines for tuberculosis: a fine line between protection and pathology.* Tuberculosis (Edinb), 2011. **91**(1): p. 82-5.
115. Richardson, J.S., et al., *Airway delivery of an adenovirus-based Ebola virus vaccine bypasses existing immunity to homologous adenovirus in nonhuman primates.* J Virol, 2013. **87**(7): p. 3668-77.
116. Holmgren, J. and C. Czerkinsky, *Mucosal immunity and vaccines.* Nat Med, 2005. **11**(4 Suppl): p. S45-53.
117. Rose, M.A., S. Zielen, and U. Baumann, *Mucosal immunity and nasal influenza vaccination.* Expert Rev Vaccines, 2012. **11**(5): p. 595-607.

118. Roy, C.J., et al., *Aerosolized adenovirus-vectored vaccine as an alternative vaccine delivery method.* Respir Res, 2011. **12**: p. 153.
119. *University of Oxford. Safety of tuberculosis vaccine, MVA85A, administered by the aerosol route and the intradermal route*. Available from: In: ClinicalTrials.gov. Bethesda:National Library of Medicine (US) 2000- [cited 2013 March 16]. Available from http://clinicaltrials.gov/show/NCT01497769 NLM Identifier:NCT01497769.
120. Jeyanathan, M., et al., *AdHu5Ag85A Respiratory Mucosal Boost Immunization Enhances Protection against Pulmonary Tuberculosis in BCG-Primed Non-Human Primates.* PLoS One, 2015. **10**(8): p. e0135009.
121. Amorij, J.P., et al., *Needle-free influenza vaccination.* Lancet Infect Dis, 2010. **10**(10): p. 699-711.
122. Omer, S.B., G.S. Hiremath, and N.A. Halsey, *Respiratory administration of measles vaccine.* Lancet, 2010. **375**(9716): p. 706-8.
123. Low, N., et al., *Immunogenicity and safety of aerosolized measles vaccine: systematic review and meta-analysis.* Vaccine, 2008. **26**(3): p. 383-98.
124. Simon, J.K., et al., *A clinical study to assess the safety and immunogenicity of attenuated measles vaccine administered intranasally to healthy adults.* Hum Vaccin, 2007. **3**(2): p. 54-8.
125. World Health Organization, *Measles Aerosol Project.*
126. Yao, Y., et al., *Induction of Autonomous Memory Alveolar Macrophages Requires T Cell Help and Is Critical to Trained Immunity.* Cell, 2018. **175**(6): p. 1634-1650 e17.
127. McCorry, T., et al., *Shedding of Mycobacterium bovis in the nasal mucus of cattle infected experimentally with tuberculosis by the intranasal and intratracheal routes.* Vet Rec, 2005. **157**(20): p. 613-8.
128. Dolovich, M.B. and D.L. Bailey, *Positron emission tomography (PET) for assessing aerosol deposition of orally inhaled drug products.* J Aerosol Med Pulm Drug Deliv, 2012. **25 Suppl 1**: p. S52-71.
129. Michotte, J.B., et al., *In Vitro Comparison of Five Nebulizers During Noninvasive Ventilation: Analysis of Inhaled and Lost Doses.* J Aerosol Med Pulm Drug Deliv, 2014.
130. *Aeroneb® Solo System Instruction Manual.* [cited 2014; Available from: http://www.aerogen.com/uploads/datasheets/30-354%20Rev%20M%20Solo%20System%20UK%20EN%20Web.pdf.
131. Cohen, B.J., et al., *Laboratory methods for assessing vaccine potency retained in aerosol outputs from nebulizers: application to World Health Organization measles aerosol project.* Vaccine, 2008. **26**(27-28): p. 3534-9.
132. Fjallbrant, H., M. Ridell, and L.O. Larsson, *BCG scar and tuberculin reactivity in children and adults.* Scand J Infect Dis, 2008. **40**(5): p. 387-92.
133. Silver, R.F., et al., *Recruitment of antigen-specific Th1-like responses to the human lung following bronchoscopic segmental challenge with purified protein derivative of Mycobacterium tuberculosis.* Am J Respir Cell Mol Biol, 2003. **29**(1): p. 117-23.
134. Busse, W.W., et al., *Investigative bronchoprovocation and bronchoscopy in airway diseases.* Am J Respir Crit Care Med, 2005. **172**(7): p. 807-16.
135. *International Conference on Harmonization (IHC). General Principles to Address Virus and Vector Shedding.* June 2009.
136. *European Medicines Agency. Guideline on quality, non-clinical and clinical aspects of live recombinant viral vectored vaccines.* 24 June 2010.

137. Public Health Agency of Canada. *Canadian Immunization Guide Part 3 Vaccination of Specific Populations*. Available from: http://www.phac-aspc.gc.ca/publicat/cig-gci/p03-eng.php.

11. APPENDICES

APPENDIX 1 - CLINICAL LABORATORY VALUES
APPENDIX 2 - STUDY FLOWCHART
APPENDIX 3 - LIST OF IMMUNOSUPPRESSIVE MEDICATIONS
APPENDIX 4 - DECLARATION OF HELSINKI
APPENDIX 5 - CRITERIA FOR TOXICITY AND ADVERSE EVENT REPORTING
APPENDIX 6 - BRONCHOSCOPY STANDARD OPERATING PROCEDURE
APPENDIX 7 - LABORATORY CHARACTERIZATION OF AEROSOL DELIVERY OF Ad5Ag85A USING AERONEB®SOLO
APPENDIX 8 - PRELIMINARY SAFETY, IMMUNOGENICITY AND RESULTS OF INDUCED SPUTUM COLLECTION

APPENDIX 1 - CLINICAL LABORATORY VALUES

Hamilton Regional Laboratory Medicine Program – Normal Laboratory Reference Intervals

Test Name	R.I.	Units	Comments
Leukocytes	4.0 - 11.0	$x10^9$/L	
Erythrocytes	4.50 - 6.50<br>3.80 - 5.80	$x10^{12}$/L<br>$x10^{12}$/L	Male<br>Female
Hemoglobin	130 - 180<br>115 - 165	g/L<br>g/L	Male<br>Female
Hematocrit	0.400 - 0.540<br>0.370 - 0.470	L/L<br>L/L	Male<br>Female
MCV	82 - 99	fL	
MCH	27 - 32	pg	
MCHC	300 - 350	g/L	
R.D.W.	11.0 - 15.0		
Platelets	150 - 400	$x10^9$/L	
M.P.V.	7.4 – 10.4	FL	
Absolute Neutrophils	2.0 - 7.5	$x10^9$/L	
Absolute Lymphocytes	1.5 – 4.0	$x10^9$/L	
Absolute Monocytes	0.2 – 0.8	$x10^9$/L	
Absolute Eosinophils	0.0 – 0.4	$x10^9$/L	
Absolute Basophils	0.0 – 0.1	$x10^9$/L	
Creatinine	60 - 115<br>50 - 100	umol/L<br>umol/L	Male<br>Female
Sodium	135 - 145	mmol/L	
Potassium	3.5 - 5.0	mmol/L	
Alkaline Phosphatase	40 - 120	U/L	
ALT	0 - 40	U/L	Male
	0 - 28	U/L	Female
Bilirubin, Total	2 - 18	umol/L	

APPENDIX 2 - STUDY FLOWCHART

Week of Treatment	Screen	Week -1	Wk 1[a]	48-72 hrs	Wk 2	Wk 4	Wk8	Wk 12	Wk16[g]	Wk24[g]
Informed Consent	X									
Inclusion/exclusion criteria	X		X							
Medical History	X		X							
Health update				X	X	X	X	X	X	X
Concomitant Medication Review	X		X	X	X	X	X	X	X	X
Physical examination[b]	X		X	X	X	X	X	X	X	X
Lab Tests										
CBC & differential, platelet count	X				X	X		X		
Na[c], K[c], AST, ALK, bilirubin, creatinine	X				X	X		X		
Serum pregnancy test	X									
Urine Pregnancy test[d]			X			X				
Serum anti-Ad5 antibody		X				X				
HIV antibody	X									
Spirometry	X				X	X	X	X		
DLCO	X									
Bronchoscopy and bronchoalveolar lavage for immunological assays[e]		X			X		X			
Chest X-ray[f]	X									
Adverse Event Evaluation	X		X	X	X	X	X	X	X	X
Interferon gamma release assay (Quantiferon Test)	X									
Blood Immunology Monitoring		X			X	X	X	X		
Ad5Ag85A vaccine administration			X							

Investigator may deviate ± 3 days from schedule dates for weeks 2 and 4 visits and ± 7 days for the remaining visits for follow-up evaluations

[a] Vaccination visit
[b] Symptom directed physical examination after screening
[c] Na and K at baseline only
[d] In women of child bearing potential; pregnancy test only repeated after 4 weeks if suspicion of pregnancy
[e] Baseline sample 1 - 6 days before vaccination; bronchoscopy at eight weeks for those participants in whom there is no contraindication
[f] Repeat chest x-ray performed if clinically indicated to evaluate respiratory symptoms

[g] The weeks 16 and 24 visits are a safety monitoring visit only; no further investigations are performed, unless clinically indicated, at this visit

APPENDIX 3 - List of immunosuppressive medications

Modified from Canadian Immunization Guide [137]

- 6-mercaptopurine - PurinetholL®
- Abatacept - Orencia™
- Adalimumab - Humira®
- Alemtuzumab - MabCampath®
- Anti-thymocyte globulin - Thymoglobulin® (
- Azathioprine - Imuran
- Basiliximab - Simulect
- Current or recent radiation
- Cyclophosphamide – Procytox, Cytoxan
- Cyclosporine - Neoralt™
- Etanercept - Enbrel®
- High-dose systemic corticosteroids (2 mg/kg per day for a child or 20 mg/day or more of prednisone or its equivalent for an adult) for 14 days or more
- Infliximab - Remicade®
- Leflunomide - Arava®
- Methotrexate
- Mitoxantrone
- Most cancer chemotherapies (except tamoxifen and hydroxyurea)
- Mycophenolatemofetil - CellCept®
- Rituximab - Rituxan®
- Sirolimus - Rapamune®
- Tacrolimus - Prograf®

APPENDIX 4 - DECLARATION OF HELSINKI

As per the Declaration of Helsinki, Ethical Principles for Medical Research Involving Human Subjects, Adopted by the 18th WMA General Assembly, Helsinki, Finland, June 1964, and amended by the
29th WMA General Assembly, Tokyo, Japan, October 1975
35th WMA General Assembly, Venice, Italy, October 1983
41st WMA General Assembly, Hong Kong, September 1989
48th WMA General Assembly, Somerset West, Republic of South Africa, October 1996, and the
52nd WMA General Assembly, Edinburgh, Scotland, October 2000

APPENDIX 5 - CRITERIA FOR TOXICITY AND ADVERSE EVENT REPORTING

This study will utilize the CTCAE Expanded Common Toxicity Criteria to report adverse events, available at:
http://evs.nci.nih.gov/ftp1/CTCAE/CTCAE_4.03_2010-06-14_QuickReference_5x7.pdf

APPENDIX 6 - BRONCHOSCOPY STANDARD OPERATING PROCEDURE

Staff:
The procedure is to be performed by qualified study personnel as approved by Site Investigator. Bronchoscopist must be a licensed physician and be skilled with obtaining good quality biopsies for research purposes. A trained assistant (nurse or laboratory technician) will be delegated to aid with preparation of bronchoscope and bronchoscopy suite, and will also be on hand to assist with monitoring the subject and specimens during the procedure. Additional research staff may be required to handle specimens as needed.

Safety:
This will be performed according to the recommendations of the U.S. National Institutes of Health as a day-case. Facilities for the management of medical emergencies and cardiopulmonary resuscitation will be available. Spirometry will be performed before and after the procedure.

Purpose:
To outline the procedure for bronchoscopy as required for each study protocol.

Equipment:
- 1%, 2% and 4% lidocaine hydrochloride solution
- 4% to 10% lidocaine hydrochloride aerosol
- Saline
- Salbutamol MDI and solution
- Nebulizer
- Atropine Sulphate
- Olympus or Pentax fibreoptic bronchoscope. The bronchoscope is cleaned using a STERIS system or other high level disinfectants for flexible bronchoscopes, within 24h of use (within 7 days if instrument is hung in a clean ventilated space) and again immediately after use. This is performed in the hospital facility by nurses/technicians trained in the proper sterilization procedure, and available to the research unit on a fee-per-use basis.
- Biopsy Forceps preferably single use. These can be serrated or non-serrated.
- Pulse oximeter
- Syringes
- 2-6 L oxygen delivered via a nasal cannula (titrated to maintain saturation above 96%)
- Midazolam **and/or** Fentanyl
- IV administration set - gravity set or pump set
- IV saline lock - for intermittent use if required per protocol.
- IV catheter - use smallest gauge to deliver required rate and/or solution as per protocol.
- Tourniquet
- Transparent dressing
- Disposable gloves
- Sharps disposal box

Pre Bronchoscopy Care/ Instructions for Patients:

1. Patients must fast for 8 hours
2. Patients will need somebody to accompany them home if sedative is administered. They should also not plan to drive, operate machinery or drink alcohol for 24 hours after having the sedative.

Procedure Preparation:

Place patient comfortably in a semi-recumbent position on the hospital bed. Position the monitor screen in direct line of vision from bronchoscopy working area. Using Intravenous Therapy SOP guidelines, insert cannula for administration of sedative, and for use in the event intravenous access is required for emergency. Select an insertion site based on the following criteria:

1. subject's age, size and general condition;
2. condition of the veins;
3. expected duration of procedure;
4. subject's preference, where possible and/or to allow easy access during the procedure;

Bronchoscopy Procedure:

1. Fibreoptic bronchoscopy is carried out according to a standardized protocol based on recent recommendation.
2. If required midazolam IV and/or Fentanyl IV is used for sedation and titrated to response by physician.
3. Either throat gargle with 4% lidocaine hydrochloride solution or lidocaine aerosol spray (10 mg/spray) administered to the posterior oropharynx
4. Pre-medication with salbutamol 200 mcg by metered dose inhaler (MDI)
5. An alternative to the lidocaine spray and salbutamol MDI is 2ml of 4% lidocaine with 2 ml salbutamol nebulizer solution administered by nebulizer to the subject.
6. Atropine 600 mcg subcutaneously (optional)
7. The fibreoptic bronchoscope is passed by the oral or nasal route with the patient in a semi-recumbent position. Topical anesthesia of the vocal cords is obtained with 1-4% lidocaine hydrochloride and below the vocal cords with 1-2% lidocaine hydrochloride (the total dose should not exceed 8 mg/kg in any subject). The total dose of lidocaine administered during the procedure is recorded.
8. After the bronchoscope is wedged in the right middle lobe bronchus, 4x 40 ml aliquots of normal saline will be introduced via the bronchoscope and then aspirated through the bronchoscope using a closed, sterile collecting system. The suction pressure will be low, and titrated for each procedure.
9. The return volume and quality of sample (colour and clarity) will be noted.
10. BAL will be processed per study protocol.
11. Every attempt is made to obtain the target volume, however this will depend on the comfort and willingness of the patient to proceed, and clinical and safety monitoring by the physician.

Patient will be allowed to recover and will be released when a study physician deems it is safe to do so. If the patient has received sedation they will be kept under observation for 1-4h or according to the bronchoscopist. They will be allowed to eat and drink 2-4 hours after the procedure or according to the bronchoscopist. Release time will be noted in the patient's charts.

Patients will be reminded of contact information of study physician should complications due to the procedure arise. Subjects should be instructed to return if they experience bleeding; if they develop fevers, sweats, chills or any other unexpected symptoms.

Approved Documentation Record:

- date and time of procedure
- volume and quality of BAL, and site obtained
- bronchoscopist's initials;
- any pertinent information regarding anaesthetic, sedative and complications during the procedure.

References:

1. National Institute of Health Workshop summary. Investigative use of bronchoscopy, lavage and bronchial biopsies in asthma and other airway diseases. *Eur Respir J* 1992;5:115-21. British Thoracic Society guidelines on diagnostic flexible bronchoscopy.
2. British Thoracic Society Bronchoscopy Guidelines Committee, a Subcommittee of the Standards of Care Committee of the British Thoracic Society. *Thorax* 2001;56 (Suppl 1):i1-i21
